# Greener Synthesis of 4‑Selanyl-Isocoumarins Mediated by TCCA: Electrochemical Insights and DNA Interaction Studies

**DOI:** 10.1021/acsomega.5c06150

**Published:** 2025-10-31

**Authors:** Luiz Eduardo Welter, Marcelo Baptista, Celso Rodrigo Nicoleti, Suzane Quintana Gomes, Fabio S. Miranda, Suelen Santos da Silva, Tiago Elias A. Frizon, Antonio Luiz Braga

**Affiliations:** † Department of Chemistry, Federal University of Santa Catarina (UFSC), 88040-970 Florianópolis, SC, Brazil; ‡ Departament of Energy and Sustainability, Federal University of Santa Catarina (UFSC), 88905-120 Araranguá, SC, Brazil; § Institute of Chemistry, Fluminense Federal University − UFF, 24220-141 Niterói, RJ, Brazil; ∥ Federal University of Santa Catarina (UFSC), 88905-120 Araranguá, SC, Brazil

## Abstract

We present a straightforward and sustainable approach for the synthesis of 4-selanyl-isocoumarins from 2-(alkynyl)-aryl or -heteroaryl and diorganoyl diselenides. This transformation employs trichloroisocyanuric acid (TCCA) as a low-cost, metal-free oxidant under ambient conditions, delivering the desired products in yields of up to 98%. Mechanistic investigations support an electrophilic activation of the alkyne followed by a 6-endo-dig cyclization. Electrochemical and DFT analyses confirm the redox activity and structural stability of the resulting compounds. Additionally, DNA binding studies, combining UV–vis spectroscopy and molecular docking, reveal dual interaction modes (intercalation and groove binding), underscoring their potential in DNA-targeted drug design.

## Graphical abstract

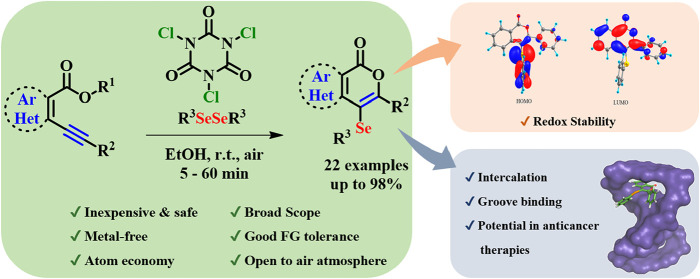

## Introduction

Isocoumarins are an important class of oxygen-containing heterocycles widely distributed in natural products^1,2^ and known for their antibacterial,^3^ anti-inflammatory,^4–6^ and anticancer properties.^7–9^ In the same way, organoselenium compounds exhibit notable biological activities,^10–13^ including antioxidant^14,15^ and antitumor effects,^16,17^ mainly due to the redox versatility of selenium and its critical role in modulating oxidative stress and enzymatic activity.^15,17–20^ The combination of these two pharmacophores in 4-selanyl-isocoumarins structures offers a promising route to structurally diverse molecules with enhanced biological activity and therapeutic potential (Figure 1).

**Figure 1:**
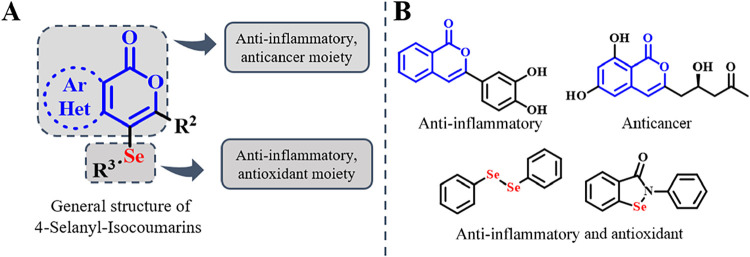
(A) General structure of 4-selanyl-isocoumarins. (B) Examples of bioactive isocoumarins and seleno compounds.

Consequently, different strategies were developed for the construction of 4-selanyl-isocoumarins from ortho-alkynyl esters, which allow electrophilic or radical cyclizations, resulting in the desired heterocyclic structure.^21–27^ Among these, an effective approach employs PhICl_2_-activated diaryldiselenides under metal-free conditions; however, the reagent is photosensitive, requires previous preparation, and demands careful manipulation.^23^ Another reported method utilizes AgNO_3_-catalyzed radical cyclization with elemental selenium and boronic acids.^24^ Although efficient, this strategy involves severe reaction conditions. Recent efforts toward more eco-friendly alternatives include methodologies based on ultrasound-assisted oxidations,^25^ electrochemical oxidation,^26^ and visible-light photoredox catalysis,^27^ which aim to reduce energy consumption and hazardous waste. These methods often face challenges related to the requirements for specialized equipment, the use of reaction additives, and the formation of undesired byproducts.

In this context, trichloroisocyanuric acid (TCCA) is a versatile and practical oxidant for activating diselenides.^28–33^ TCCA is a stable, nonhygroscopic, inexpensive, and commercially available white solid, commonly used in sanitation and disinfection.^34–36^ Its ease of manipulation, high oxidative potential, and compatibility with organic solvents have increased its use in synthetic organic chemistry.^36^ Specifically, it enables the *in situ* generation of electrophilic selenium species under mild conditions without needing metal catalysts or external activators.^29,30,32^ Despite these benefits, its use in synthesizing selanyl-isocoumarins remains unexplored.

Herein, we report a sustainable and metal-free method for the synthesis of 4-selanyl-isocoumarins using TCCA under mild conditions. Additionally, the biological potential of selected compounds was investigated through DNA binding studies using UV–vis spectroscopy and molecular docking, revealing dual interaction modes and highlighting their potential therapeutic relevance.

## Experimental Section

### Cyclic Voltammetry

The electrochemical behavior of some selected compounds was studied by cyclic voltammetry (CV) in the potential range from −2.5 to 2.5 V vs Ag/Ag^+^. All CV measurements were performed in a potentiostat/galvanostat PALMSENS EMSTAT4S LR, with a standard three-electrode glass electrochemical cell, using glassy carbon as the working electrode, a platinum wire as the auxiliary electrode, and Ag/Ag^+^ (AgNO_3_ 0.01 mol L^–1^ in acetonitrile) as the reference electrode. All measurements were carried out at 25 °C in anhydrous acetonitrile containing 0.1 mol L^–1^ of tetra-*n*-butylammonium hexafluorophosphate (TBAPF_6_) as the supporting electrolyte. The analyte solutions at around 1 mol L^–1^ were degassed with argon to remove dissolved oxygen. All voltammograms were referenced to the normal hydrogen electrode (NHE) using the value of 630 mV of the internal standard ferrocene/ferrocenium (Fc/Fc^+^) redox couple in acetonitrile.^37^ The electrochemical HOMO and LUMO energies were calculated using eqs 1 and 2:^38^

1 $$E_{HOMO}=-\left(E_{\left[onset,\;ox\;vs\;NHE\right]}+4.75\right)\;\left(eV\right)$$

2 $$E_{LUMO}=-\left(E_{\left[onset,\;red\;vs\;NHE\right]}+4.75\right)\;\left(eV\right)$$

### Computational Details

All calculations using density functional theory (DFT) and time-dependent DFT (TD-DFT) were performed using Orca 6.0.1 software.^39^ The geometries of all compounds were optimized at the PBE0/def2-TZVP level, with the solvent effects (acetonitrile) accounted by C-PCM Model.^40^ Dispersion correction was included with the D4 model (charge-dependent atom-pairwise dispersion correction).^41^ The conformation choice for guess geometries of all molecules was based on the geometry of the 3a, which was obtained from an ab initio molecular dynamic (AIMD) at PBE0-D3/def2-TZVP/COSMO in annealing mode in Turbomole 6.6 software.^42^ Harmonic frequency calculations were also carried out to ensure the geometries obtained were global minima. Once no negative or imaginary frequencies were observed, the stability of the geometries was confirmed. The 3D surfaces (canonical orbitals–MO) and spin densities were plotted using the orca_plot tool followed by visualization with Chemcraft 1.8 software.^43^

### UV–Visible Scanning Spectrophotometric Titration

The possible interaction of selected complexes 3 with calf thymus DNA (CT-DNA) was assessed through a UV–visible spectrophotometric titration. Increasing concentrations of the complexes (0–100.3 μmol L^–1^) were used for the absorption titration assay while the concentration of CT-DNA (150 μmol L^–1^) was kept constant. The stock solution of Ct-DNA in Milli-Q water yielded an absorbance ratio at 260 and 280 nm (A260/A280) of ≥1.8–1.9, indicating the solution was sufficiently free from protein contaminant residues.^44^ After each addition, the solution was mixed and incubated for 5 min before recording the absorption spectra. To obtain the spectra of the samples, a Hitachi U-2910 spectrophotometer was used, and UV–visible scanning was performed from 200 to 500 nm. The changes in CT-DNA absorbance, after incubation of the compounds, as well as the maximum absorption wavelength shift, were determined, and the experiments were repeated three times.^45^

### Molecular Docking

Molecular docking simulations were performed using AutoDockVina^46^ since this software is parametrized for selenium.^47–49^ DNA crystallographic structure was downloaded from Protein Data Bank (PDB ID: 1Z3F) and prepared using AutoDockTools^50^ by removing water, ligands, and cofactors and adding hydrogen atoms to the structure. Docking simulations were performed using a blind-docking procedure; i.e., the entire surface of the DNA was considered, so the ligand interacted with the one with the greatest affinity. A gridbox of 68 × 60 × 78 Å^3^ was used and centered in the coordinates *x* = 0.8, *y* = 15.0, *z* = 37.9. The pose with the highest binding energy (BE) had the chemical interactions analyzed using the Biovia Discovery Studio Visualizer software.^51^

### General Procedure for the Synthesis of 4-(Selanyl)-1H-isochromen-1-ones 3

To a glass tube were added TCCA (trichloroisocyanuric acid, 0.0875 mmol), diorganoyl diselenide (0.1875 mmol), and anhydrous ethanol (3.0 mL) at room temperature under stirring for 5 min. After, the starting material 2-(alkynyl)-aryl or -heteroaryl esters (0.25 mmol) were added. The progress of the reaction was monitored by TLC, and the formation of a white precipitate (product) was observed, which interrupted magnetic stirring. Thus, the reaction was immediately stopped, and the mixture was extracted with ethyl acetate (15 mL) and washed with water (3 × 15 mL). The organic phase was dried over MgSO_4_ and concentrated under reduced pressure. The final product was isolated through flash column chromatography using silica gel as the stationary phase and eluted with a mixture of hexane and ethyl acetate. Product characterization data can be found in the Supporting Information.

## Results and Discussion

### Greener Synthesis of 4-Selanyl-isocoumarins

The reaction between ortho-alkynylbenzoate **1a** and diphenyl diselenide **2a** was optimized by using TCCA as a chlorinating agent in anhydrous ethanol (Table 1). Initially, equimolar amounts of **1a** and **2a** with 0.125 mmol of TCCA in anhydrous EtOH produced product **3a** in 96% yield after 15 min (entry 1). During this reaction, the formation of a white precipitate (product) disrupted the magnetic stirring, prompting immediate extraction. When no precipitation occurred, the reaction was monitored for up to 60 min. In cases where no product was formed or the yield was very low, the starting material was recovered.

**Table 1:**
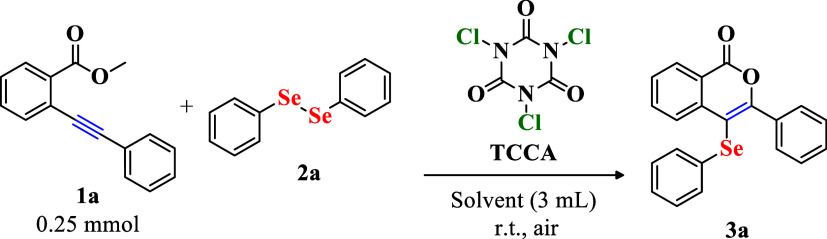
Optimization of Reaction Conditions for the Synthesis of 3-Phenyl-4-(phenylselanyl)-isocoumarin 3a^f^

entry	solvent	TCCA (mmol)	**2a** (mmol)	time (min)	yeld (%)^a^
1	EtOH^b^	0.125	0.250	15	96
2	EtOH^b^	0.100	0.250	15	93
3	EtOH^b^	0.088	0.250	5	98
4	EtOH^b^	0.088	0.138	15	55
5	EtOH^b^	0.088	0.163	5	87
6	EtOH^b^	0.088	0.188	5	97
7	EtOH 96%	0.088	0.188	30	76
8	MeOH^b^	0.088	0.188	5	89
9	H_2_O	0.088	0.188	60	trace
10	ethyl lactate	0.088	0.188	60	65
11	2-Me-THF^b^	0.088	0.188	60	30
12	PEG-400	0.088	0.188	60	68
13	glycerol	0.088	0.188	60	N.R.
14	cyrene	0.088	0.188	60	N.R.
15	EtOAc^b^	0.088	0.188	15	71
16	CH_2_Cl_2_ ^b^	0.088	0.188	15	64
17	EtOH^b^	0.088	0.188	5	91^c^
18	EtOH^b^	0.088	0.188	5	90^d^
19	EtOH^b^	0.088	0.188	5	93^e^

a Isolated yield by column chromatography.

b Anhydrous solvent.

c Temperature of 65 °C.

d Inert argon atmosphere.

e Positive pressure of atmospheric oxygen.

f Reaction conditions: **1a** (0.25 mmol), solvent (3 mL); r.t. = room temperature; N.R. = did not react, starting material recovered.

A further reduction in TCCA to 0.10 mmol provided a comparable yield (93%, entry 2), and an additional reduction to 0.088 mmol improved the yield to 98% in just 5 min (entry 3), establishing this as the optimal amount.

Variations in the quantities of diselenide **2a** indicated that substoichiometric amounts of this compound decreased the efficiency (55% with 0.138 mmol, entry 4), while 0.163 and 0.188 mmol restored high yields (87% and 97%, entries 5 and 6, respectively).

The effect of the solvent was also evaluated. Substituting anhydrous ethanol with 96% ethanol resulted in a reduced yield (76%, entry 7), highlighting the importance of strictly anhydrous conditions. In contrast, methanol was effective (89%, entry 8), whereas water led to only trace amounts of the desired product (entry 9). Ethyl lactate, PEG-400. and 2-Me-THF provided moderate yields (entries 10–12), whereas glycerol and cyrene were ineffective (entries 13 and 14). Common organic solvents such as ethyl acetate and CH_2_Cl_2_ provided lower yields of the desired product (71% and 64%, entries 15 and 16). Among the solvents tested, anhydrous ethanol emerged as particularly effective due to its superior ability to dissolve both the starting materials and the reaction intermediates. Additionally, it likely stabilizes ionic species, contributing to the overall reaction efficiency.

To further assess the influence of environmental factors on the optimized reaction, additional experiments were conducted under controlled temperature and atmosphere conditions (entries 17–19). The reaction carried out at 65 °C (entry 17) resulted in a modest yield of 91%, indicating that elevated temperatures do not increase the efficiency of the reaction. Carrying out the reaction in an argon atmosphere (entry 18, 90%) or a pure O_2_ atmosphere (entry 19, 93%) produced yields comparable to those obtained in ambient air. This result suggests that the process occurs efficiently, without the need for an external oxidant to convert substrates **1a** and **2a** into product **3a**. These findings demonstrate that employing 0.088 mmol of TCCA and 0.188 mmol of **2a** in anhydrous ethanol at room temperature, under ambient open-air conditions, affords excellent yields, highlighting the reaction’s efficiency under mild and environmentally friendly conditions.

Following the establishment of optimized conditions, the reaction scope was investigated through systematic variation of the substituents on substrate **1** (Scheme 1). Variation of the ester group (R = Me in **1a**, Bz in **1n**, and H in **1o**) led to the formation of compound **3a** in excellent yields of 97%, 98%, and 90%, respectively. For some substrates, the reaction time was adjusted to ensure complete consumption of the starting material, while all other optimized conditions were maintained. Even in the absence of precipitate formation, the reactions reached completion within 60 min, demonstrating both the efficiency and relevance of the protocol.

**Scheme 1:**
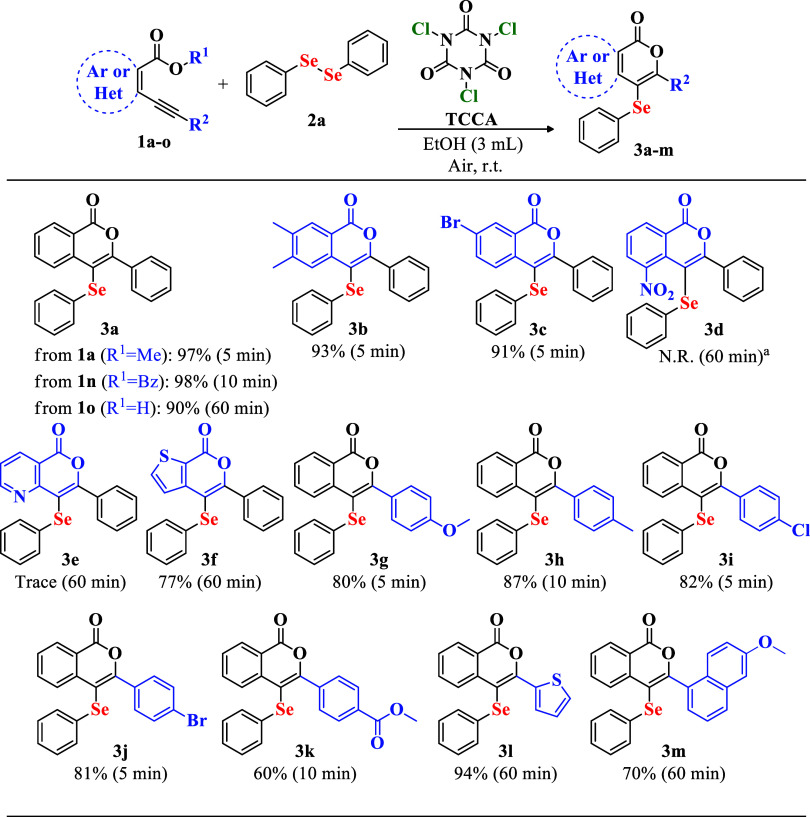
Scope of Reaction for the Preparation of 3-Aryl-4-(phenylselanyl)-isocoumarins **3** from Different 2-(Alkynyl) Esters **1** ^b^

a Despite the one-hour reaction period, no reaction (N.R.) persisted, and the substrate was subsequently recovered.
b Reaction conditions: substrate **1** (0.25 mmol), diphenyl diselenide **2a** (0.1875 mmol), TCCA (0.0875 mmol), EtOH (3 mL), r.t. and open-air. Isolated by column chromatography.

Thus, methyl-substituted substrate **1a** (R^1^ = Me) was chosen for subsequent studies. The position of the aromatic and heteroaromatic substituents relative to the alkyne moiety influenced the reactivity of the substrates. Substrates bearing substituents away from the *ortho* position to the alkyne, such as **3b** and **3c**, afforded the desired products in high yields (93% and 91%, respectively). In contrast, the nitro-substituted substrate **1d**, with the group in the *ortho* position with respect to the alkyne, was unreactive, indicating a marked decrease in triple-bond reactivity. For substrate **1e**, the presence of a pyridine ring in close spatial proximity could attenuate the electrophilicity of selenium (PhSe^+^) through a nonbonding interaction,^52^ thereby impeding the transformation and resulting in only trace amounts of **3e**. Furthermore, the thiophene-substituted product **3f** was obtained in 77% yield.

Substituents on the alkyne moiety (R^2^) were also evaluated. Electron-donating and electron-withdrawing groups in the *para* position of the benzene ring produced products **3g**–**3k** in yields ranging from 60% to 87%. The incorporation of thiophene and naphthalene groups resulted in the formation of products **3l** and **3m** in yields of 94% and 70%, respectively, highlighting the versatility of the methodology.

Subsequently, the use of various diorganoyl diselenides (**2**) was investigated using substrate **1a** (Scheme 2). Diselenides bearing electron-donating groups yielded compounds **3n**, **3o**, and **3p** in yields of 93%, 89%, and 95%, respectively. Electron-withdrawing substituents afforded products **3q**, **3r**, and **3s** in yields of 89%, 96%, and 92%, respectively. Diselenides containing benzyl and naphthalene groups led to the formation of **3t** and **3u** in yields of 93% and 76%, respectively, while the alkyl-substituted diselenide produced **3v** in a yield of 76%. These results confirm the method’s broad compatibility with a wide variety of diselenides.

**Scheme 2:**
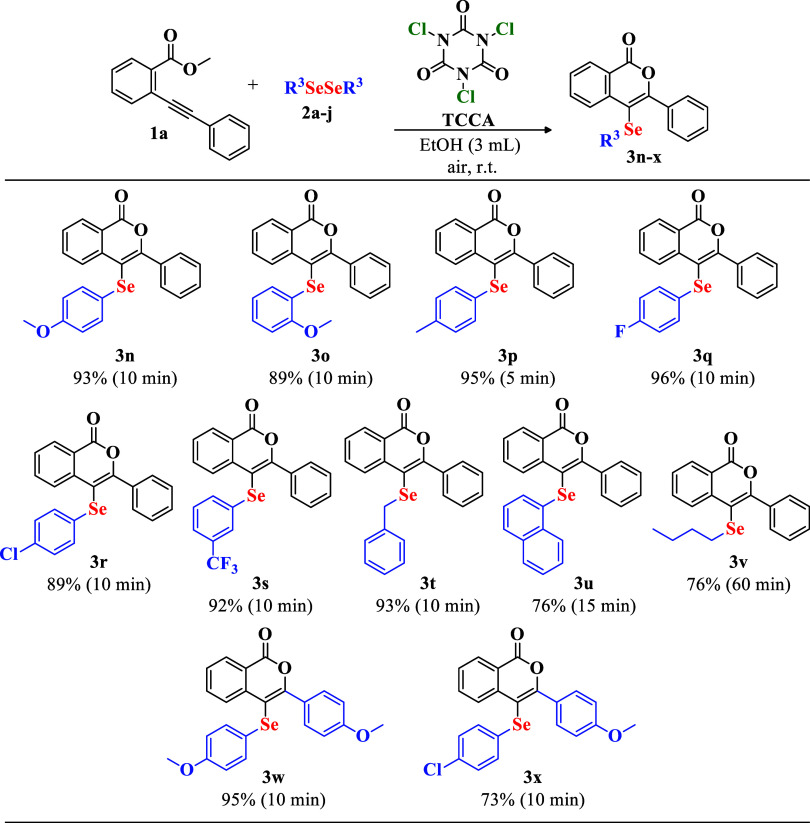
Scope of Reaction for the Preparation of 3-Phenyl-4-(selanyl)-isocoumarins **3** from Different Diorganoyl Diselenides **2** ^a^

a Reaction conditions: methyl 2-(phenylethynyl)­benzoate **1a** (0.25 mmol), diorganoyl diselenide **2** (0.1875 mmol), TCCA (0.0875 mmol), EtOH (3 mL), r.t., and open-air. Isolated by column chromatography.

To evaluate the scalability of the developed method, the intramolecular cyclization of methyl 2-(phenylethynyl)­benzoate (**1a**) with diphenyl diselenide (**2a**) in the presence of TCCA was performed on a gram scale (starting from 4.24 mmol of **1a**). This reaction yielded compound **3a** in 91% yield after only 7 min (Scheme 3). These results demonstrate that the developed methodology for the synthesis of 4-selanyl-isocoumarins is practical, cost-effective, and efficient for larger-scale applications.

**Scheme 3:**
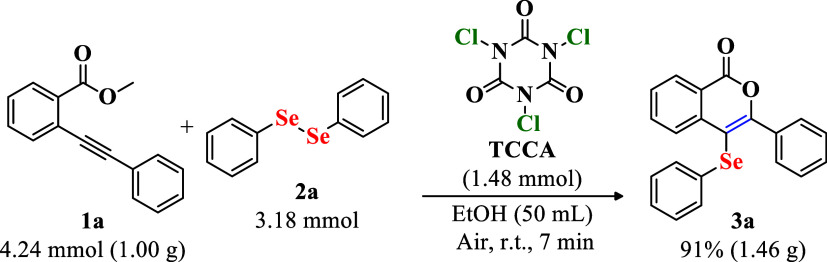
Gram-Scale Synthesis of 3-Phenyl-4­(phenylselanyl)-isocoumarin **3a** ^a^

a Reaction conditions: methyl 2-(phenylethynyl)­benzoate **1a** (4.24 mmol), diphenyl diselenide **2a** (3.18 mmol), TCCA (1.48 mmol), EtOH (50 mL), r.t., and air. Isolated by column chromatography.

Based on the literature^29–33^ and the control experiments using radical scavengers performed (Scheme S1), a plausible ionic mechanism for the formation of 4-selanyl-isocoumarins **3** is proposed in Scheme 4. The reaction begins with stirring diphenyl diselenide **2** and TCCA in ethanol for 5 min, which likely promotes the oxidative cleavage of the Se–Se bond and formation of electrophilic selenium intermediates **I** and/or **II**. These species are capable of generating reactive RSe^+^ cations *in situ*. Upon addition of substrate **1**, the electrophilic selenium species most likely adds regioselectively to the alkyne moiety, forming stabilized seleniranium intermediate **III**. This activation enhances the electrophilicity of the triple bond, enabling an intramolecular nucleophilic attack by the carbonyl oxygen of the ester, which proceeds through a 6-*endo-dig* cyclization pathway to afford intermediate **IV**. The final demethylation step occurs via nucleophilic substitution, where the chloride ion attacks the methyl group from the ester, affording target compound **3**.

**Scheme 4:**
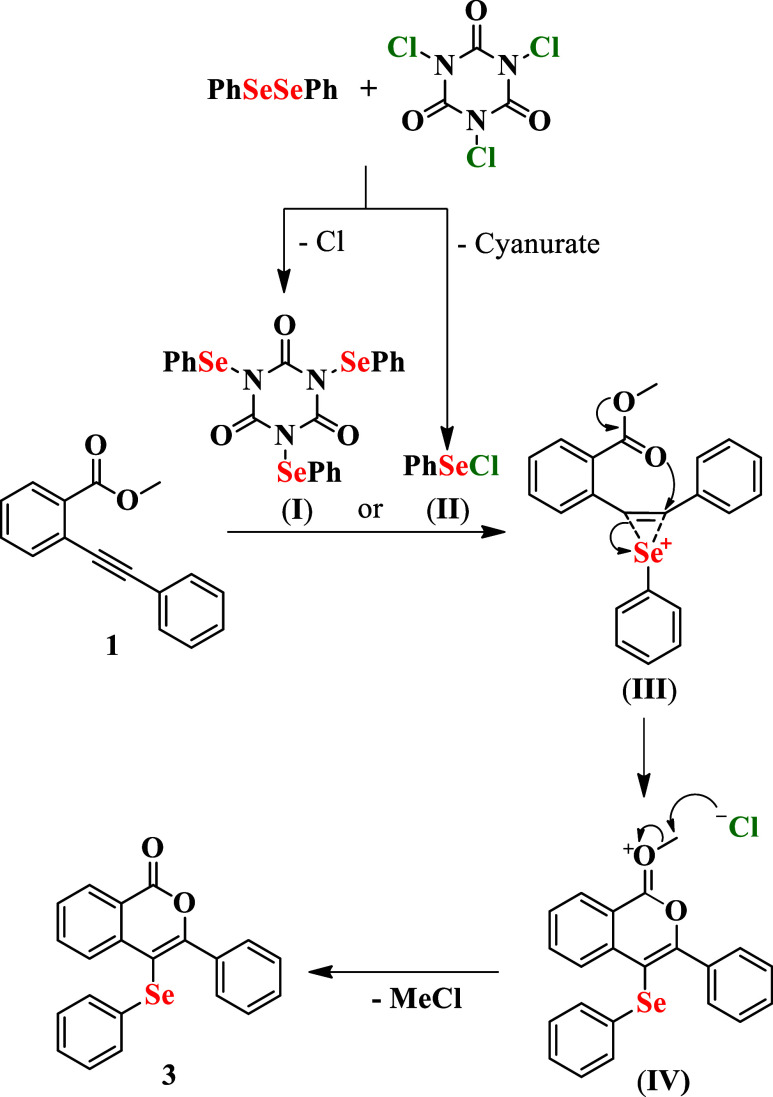
Plausible Mechanism of 4-(Phenylselanyl)-isocoumarins **3**

Furthermore, given the importance of selenoxide derivatives,^53^ the synthesis of 3-phenyl-4-(phenylseleninyl)-1H-isochromen-1-one (**4a**) was performed from compound **3a**, using meta-chloroperbenzoic acid in dichloromethane, as previously reported in the literature.^54^ The target compound was obtained in a 93% yield after 30 min of reaction (Scheme 5).

**Scheme 5:**
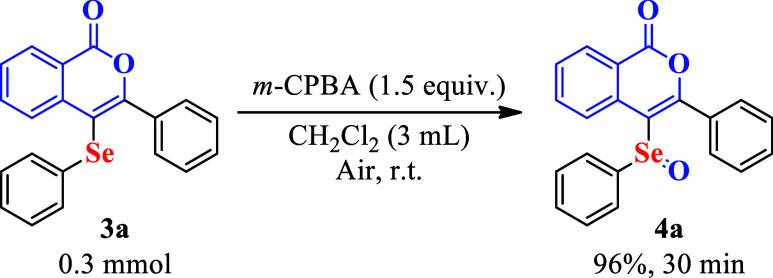
Functionalization of 3-Phenyl-4­(phenylselanyl)-isocoumarin **3a** ^a^

a Reaction conditions: **3a** (0.3 mmol), meta-chloroperoxybenzoic acid 77% (0.078 g, 1.5 equiv), DCM (5.0 mL), 0 °C, air. Isolated by column chromatography.

For the electrochemical characterization and DNA interaction, the selection of compounds was made based on the diversity of substituent groups, including both electron-donating and electron-withdrawing groups and their positional variations within the molecular structure.

### Electrochemical Characterization

Some selected compounds derived from **3a** were characterized by cyclic voltammetry. The electrochemical behavior of the series is dominated by irreversible peaks with adsorption on the surface, characterized by the linearity of the peak current vs scan rate (*i*_p_ vs υ).^55^ The irreversibility may be associated with the adsorption of the formed species (oxidized and reduced) on the electrode surface. Figure 2 shows the cyclic voltammogram of **3a**, the compound presents two irreversible oxidation peaks at 1.82 and 2.52 V, and a reduction peak at −1.70 V. Compounds **3c** and **3k** presented two reduced peaks. The only compound that does not present two oxidation waves was **3j**, probably due to the electronic effect of the bromine atom, which shifts the second oxidation potential to a high value. The voltammograms for all of the studied compounds are presented in the Supporting Information.

**Figure 2:**
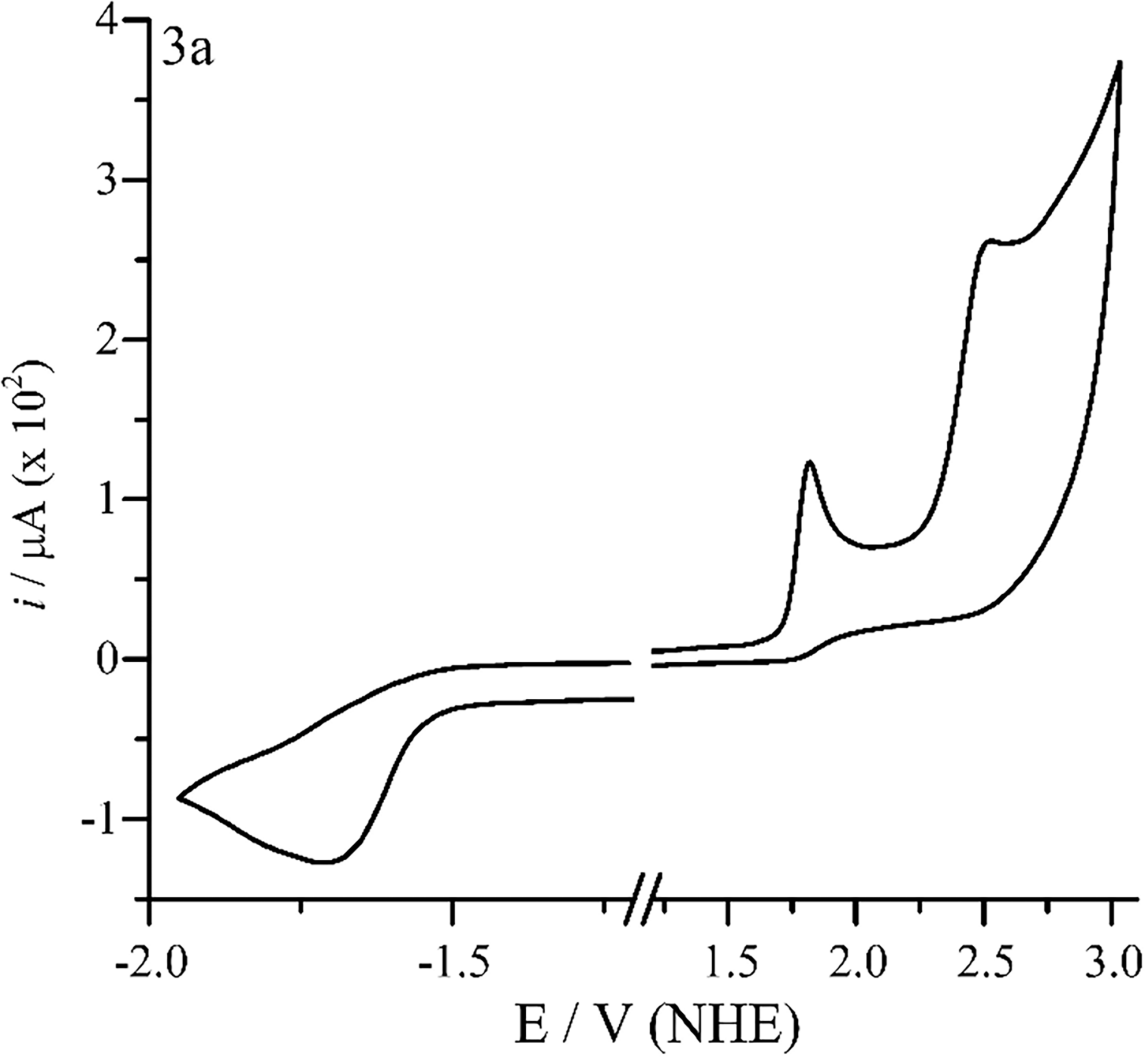
Cyclic voltammetry of **3a** in acetonitrile at 100 mV s^–1^.

Table 2 summarizes the electrochemical results. The potentials observed indicate that all compounds are stable to air, as all them are very resistant to oxidation and reduction. From the first oxidation and reduction peaks, it was possible to calculate the HOMO, LUMO, and electrochemical gap. The HOMOs range from −6.55 to −6.26 eV, it was observed that compound **3w** has the highest value (−6.26 eV) in agreement with the electronic effect of two methoxy groups in rings C and D, it was followed by compound **3g** (−6.36 eV), which contains one group methoxyl in ring C, these are the easiest compounds to oxidate among the selected studied compounds. On the other side is the compound **3s** (−6.55 eV) which contains the ring D substituted by the −CF_3_ group. It is followed by compound **3k** (−6.53 eV), which also contains a withdraw group, a −CO_2_Me in ring C. Compound **3s** is the hardest to oxidate followed by **3k**. The LUMOs range was −3.19 to −3.50 eV, compound **3k** (−3.50 eV) has the highest tendency to reduce, followed by **3c** (−3.42 eV), where the bromine atom in ring A shows a significant withdrawal effect in the coumarin ring. The smallest tendency to reduction occurs in the compounds with donor substitutes such as **3p** and **3w**. The gaps presented values from 3.03 to 3.24 with no direct correlation with the substituents.

**Table 2:** Summary of the Electrochemistry Results of Selected Compounds

	*E*_red1_	*E*_red2_	*E*_ox1_	*E*_ox2_	HOMO	LUMO	gap
**3a**	–1.70		1.82	2.52	–6.44	–3.21	3.23
**3c**	–1.44	–1.69	1.84	2.54	–6.48	–3.42	3.06
**3f**	–1.61		1.74	2.44	–6.36	–3.27	3.09
**3g**	–1.68		1.72	2.17	–6.36	–3.27	3.09
**3h**	–1.68		1.78	2.43	–6.43	–3.24	3.19
**3i**	–1.68		1.84	2.63	–6.47	–3.28	3.19
**3j**	–1.69		1.91		–6.51	–3.29	3.22
**3k**	–1.38	–1.66	1.91	2.11	–6.53	–3.50	3.03
**3p**	–1.69		1.78	2.54	–6.40	–3.19	3.21
**3s**	–1.65		1.92	2.60	–6.55	–3.31	3.24
**3w**	–1.76		1.62	2.15	–6.26	–3.20	3.06
**3x**	–1.72		1.74	2.22	–6.40	–3.24	3.16

To support the electrochemistry analysis, DFT calculations at the PBE0-D4/def2-TZVP level and solvent effects accounted by the C-PCM method (MeCN) were carried out in detail for **3a** and its first reduction species (anion radical) and first oxidate species (cation radical). Figure 3 shows the frontier orbitals of **3a** in neutral form and spin densities of the anion and cation radical. The HOMO of **3a** is localized in the Se-phenyl (ring D) point out for the oxidant site; this is corroborated by the spin density of the cation radical over the Se site. The LUMO of **3a** is localized mostly over the isocoumarin ring, while the spin density shows distribution over the rings B and C. This is a result of a decrease of the torsion angle (τ_2_ = 20.3°) between rings B and C, against a torsion angle of 50.2° for the neutral form and 46.6° for cation radical. In summary, the reduction increases the planarity between the isocoumarin moiety and the ring C.

**Figure 3:**
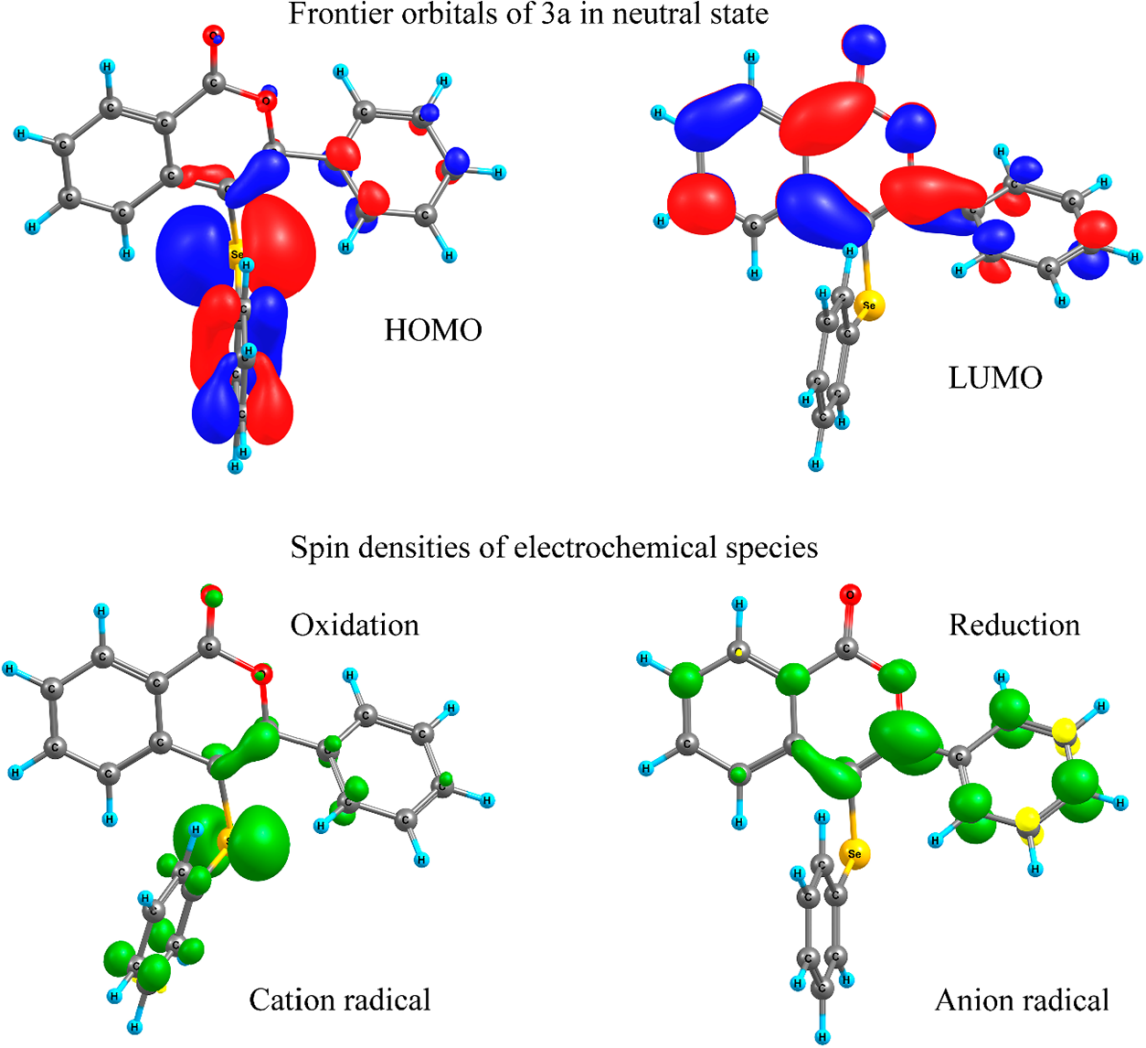
(Top) Frontier molecular orbitals for **3a**, at 0.04 au isosurface. (Bottom) Spin densities for the first oxidate species (cation radical) on the right, and first reduced species (Anion radical) on the left, both at 0.006 au.

Additionally, the ground state of all compounds **3a**-**3x** was calculated at the same level of theory as **3a**, and all geometries were based on the conformation of **3a**. The selected structural parameters for all compounds are listed in Table 3 and Scheme 6. The biggest changes in the structural parameters are observed for the electrochemical species of **3a**, under reduction and oxidation, the bond C–Se decreases more substantially in the oxidate form, while the Se–C bond presents different changes, decreases with oxidation, and increases with the reduction. The isocoumarin bonds are more sensitive to the reduction. In general, the molecules can be divided into different groups; with modifications in ring A (**3a**–**3f**), modifications in ring C (**3g**–**3m**), and modifications in ring D (**3n**–**3s**, **3u**), molecules **3t** and **3v** have the most different pattern with alkyl chain, molecules **3w** and **3x** have modifications in rings simultaneous in rings C and D. In summary, all compounds do not present coplanarity between the rings. The structural parameters have slight changes that are dependent on the position and substituent nature. The most significant changes are observed in the groups of **3a**–**3f**, where modifications of ring A cause structural changes in the isocoumarin ring, especially in the CC^cou^ bond.

**Scheme 6:**
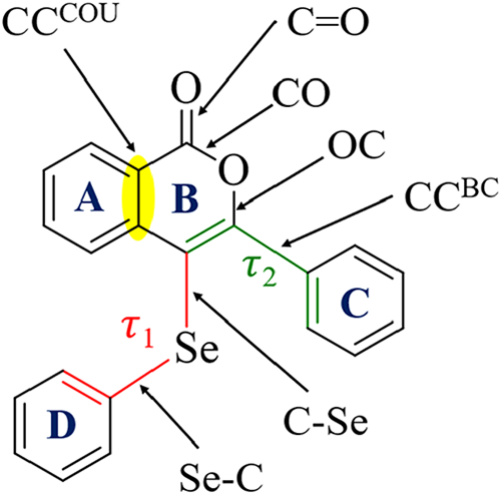
Structure Parameters

**Table 3:** Selected Structural Parameters from DFT Calculations (PBE0-D4/def2-TZVP/C-PCM)

	C–Se	Se–C	C–Se–C	CC^cou^	C=O	CO	OC	CC^BC^	τ_1_	τ_2_
**3a**	1.901	1.911	100.5	1.405	1.206	1.365	1.360	1.469	11.1	50.2
**3a**-ox	1.873	1.876	102.5	1.406	1.198	1.382	1.337	1.454	25.7	46.6
**3a**-red	1.895	1.914	102.7	1.425	1.219	1.348	1.401	1.421	0.8	20.3
**3b**	1.901	1.910	100.5	1.402	1.207	1.366	1.360	1.469	11.9	50.3
**3c**	1.901	1.911	100.4	1.403	1.205	1.363	1.361	1.468	11.8	49.8
**3d**	1.905	1.909	102.7	1.410	1.203	1.359	1.355	1.470	1.9	52.3
**3e**	1.898	1.908	100.8	1.402	1.204	1.365	1.359	1.467	8.8	49.0
**3f**	1.898	1.911	99.8	1.386	1.208	1.375	1.358	1.468	15.8	49.3
**3g**	1.901	1.910	100.8	1.405	1.206	1.364	1.362	1.463	9.3	45.9
**3h**	1.901	1.910	100.6	1.405	1.206	1.364	1.361	1.466	10.4	48.5
**3i**	1.902	1.911	100.4	1.405	1.205	1.365	1.360	1.468	11.4	49.2
**3j**	1.902	1.911	100.4	1.405	1.205	1.365	1.360	1.468	11.4	49.3
**3k**	1.901	1.911	100.3	1.405	1.205	1.366	1.359	1.470	13.4	51.0
**3l**	1.900	1.912	100.7	1.405	1.205	1.365	1.363	1.447	2.48	31.5
**3m**	1.899	1.912	99.8	1.404	1.205	1.367	1.360	1.475	16.6	80.6
**3n**	1.901	1.912	99.9	1.405	1.206	1.364	1.361	1.469	17.8	50.5
**3o**	1.904	1.909	99.3	1.405	1.206	1.365	1.360	1.468	3.87	49.3
**3p**	1.901	1.911	100.3	1.405	1.206	1.364	1.361	1.469	12.3	50.2
**3q**	1.901	1.911	100.1	1.405	1.206	1.365	1.360	1.469	15.7	50.7
**3r**	1.901	1.908	100.3	1.405	1.206	1.365	1.360	1.469	11.5	50.5
**3s**	1.901	1.908	100.3	1.405	1.205	1.366	1.359	1.469	9.9	50.2
**3t**	1.907	1.974	99.4	1.405	1.207	1.362	1.363	1.470	172.6	53.0
**3u**	1.901	1.913	100.0	1.405	1.206	1.365	1.360	1.468	7.6	49.3
**3v**	1.908	1.960	99.5	1.405	1.207	1.361	1.365	1.472	63.1	58.5
**3w**	1.901	1.912	100.3	1.405	1.207	1.363	1.362	1.463	13.7	45.9
**3x**	1.901	1.908	100.6	1.405	1.206	1.364	1.361	1.463	9.3	46.1

Table 4 presents the calculated electronic parameters as HOMO, LUMO, gap, electrophilicity index (ω),^56–60^ global electronegativity (χ),^56–60^ and dipole moment (μ). Since the proposal by Pal et al.^57^ of the electrophilicity index, it has been used as the parameter of reactivity and descriptor for biological activity. Due to the lack of information for all compounds, a correlation was carried out with the electrochemically studied compounds. A satisfactory linear correlation was found with the first reduction potential, as shown in Figure 4.

**Figure 4:**
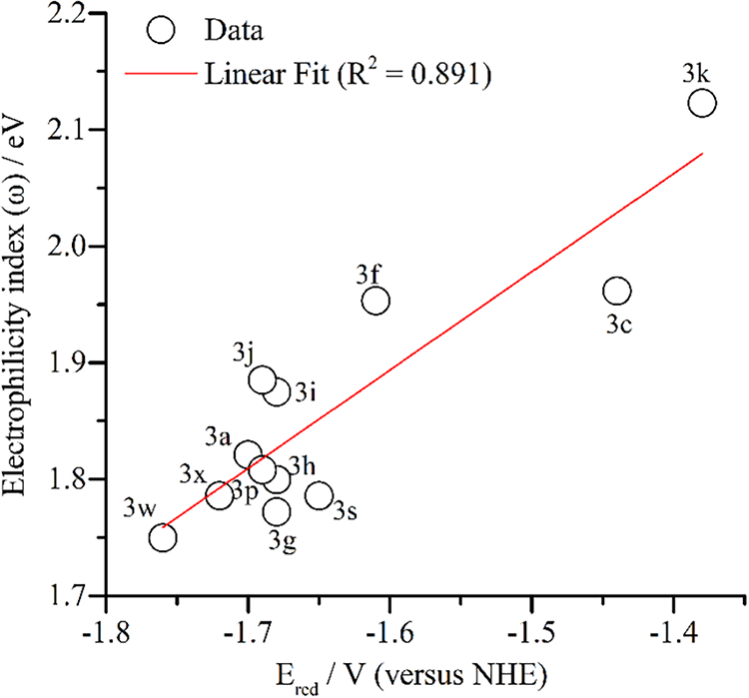
Linear correlation between *E*_red_ vs the electrophilicity index.

**Table 4:** Electronic Parameters Obtained from the DFT Calculations

	HOMO	LUMO	GAP	ω^a^	χ^b^	μ^c^
**3a**	–6.17	–1.80	4.37	1.82	3.99	5.38
**3b**	–6.14	–1.69	4.45	1.72	3.91	6.18
**3c**	–6.20	–1.96	4.24	1.96	4.08	6.22
**3d**	–6.31	–2.42	3.89	2.45	4.36	0.60
**3e**	–6.10	–1.96	4.14	1.96	4.03	2.68
**3f**	–6.18	–1.95	4.23	1.95	4.06	6.49
**3g**	–6.08	–1.75	4.33	1.77	3.92	4.09
**3h**	–6.14	–1.78	4.36	1.80	3.96	5.40
**3i**	–6.19	–1.86	4.33	1.87	4.03	5.96
**3j**	–6.20	–1.88	4.32	1.89	4.04	6.04
**3k**	–6.22	–2.12	4.10	2.12	4.17	7.95
**3l**	–6.18	–2.01	4.18	2.01	4.10	5.82
**3m**	–6.05	–1.75	4.30	1.77	3.90	5.04
**3n**	–5.87	–1.79	4.09	1.79	3.83	5.19
**3o**	–6.05	–1.80	4.26	1.81	3.93	7.45
**3p**	–6.06	–1.79	4.26	1.81	3.93	5.96
**3q**	–6.14	–1.81	4.34	1.82	3.98	4.38
**3r**	–6.18	–1.82	4.36	1.83	4.00	4.14
**3s**	–6.09	–1.77	4.33	1.79	3.93	1.74
**3t**	–6.30	–1.75	4.55	1.78	4.03	5.91
**3u**	–6.03	–1.82	4.22	1.83	3.92	5.60
**3v**	–6.20	–1.72	4.47	1.75	3.96	6.30
**3w**	–5.83	–1.74	4.09	1.75	3.78	3.54
**3x**	–6.09	–1.77	4.33	1.79	3.93	3.64

a Electrophilicity index: $\omega =\frac{\chi^{2}}{2\eta }$ , where η = *E*_LUMO_ – *E*_HOMO_ is the global hardness.

b $\chi =\mathrm{1/2}\left(E_{\mathrm{HOMO}}+E_{\mathrm{HOMO}}\right)$ is the global electronegativity.

c μ is the dipole moment of the molecule.^56–60^

The highest value of electrophilicity was found for **3d** (–NO_2_ derivative), followed by **3k** and **3l**, which have electron-withdrawing substituents. While the smallest values were observed for **3b** < **3w** < **3v**, which agrees with the donor effect of the substituents. The calculated global electronegativity is almost linear with the electrophilicity index; however, it has some changes due to the mathematical formalism. The dipole moment is a good property to analyze how the structural changes affect the polarity of the molecule. The most polar compounds are **3k** > **3o** > **3f** > **3v**, and the less polar are **3d** < **3s** < **3e**.

### Spectrophotometrically DNA-Complex Interaction Measurements

The spectrophotometric measurements of the DNA-compound interaction are presented in Figure 5 as graphs showing the results of the titration of the compounds at the UV absorbance peak of the DNA (260 nm). Compounds **3a**, **3b**, **3f**, **3h**, **3k**, **3p**, **3w**, and **3x** showed a strong hypochromic effect, characteristic of intercalation with DNA. Intercalation occurs when planar, aromatic molecules insert between adjacent base pairs in the DNA double helix.^61^ This interaction disrupts the electronic environment of the nucleobases, leading to enhanced base stacking and compaction of the helical structure. As a result, the absorbance at 260 nm decreases due to reduced π–π* transitions within the aromatic systems of the DNA bases. The observed hypochromic behavior highlights the ability of these compounds to stabilize the DNA helix by altering its structural and electronic properties. Such interactions are commonly associated with mechanisms that inhibit transcription and replication by interfering with the DNA’s accessibility.^61^

**Figure 5:**
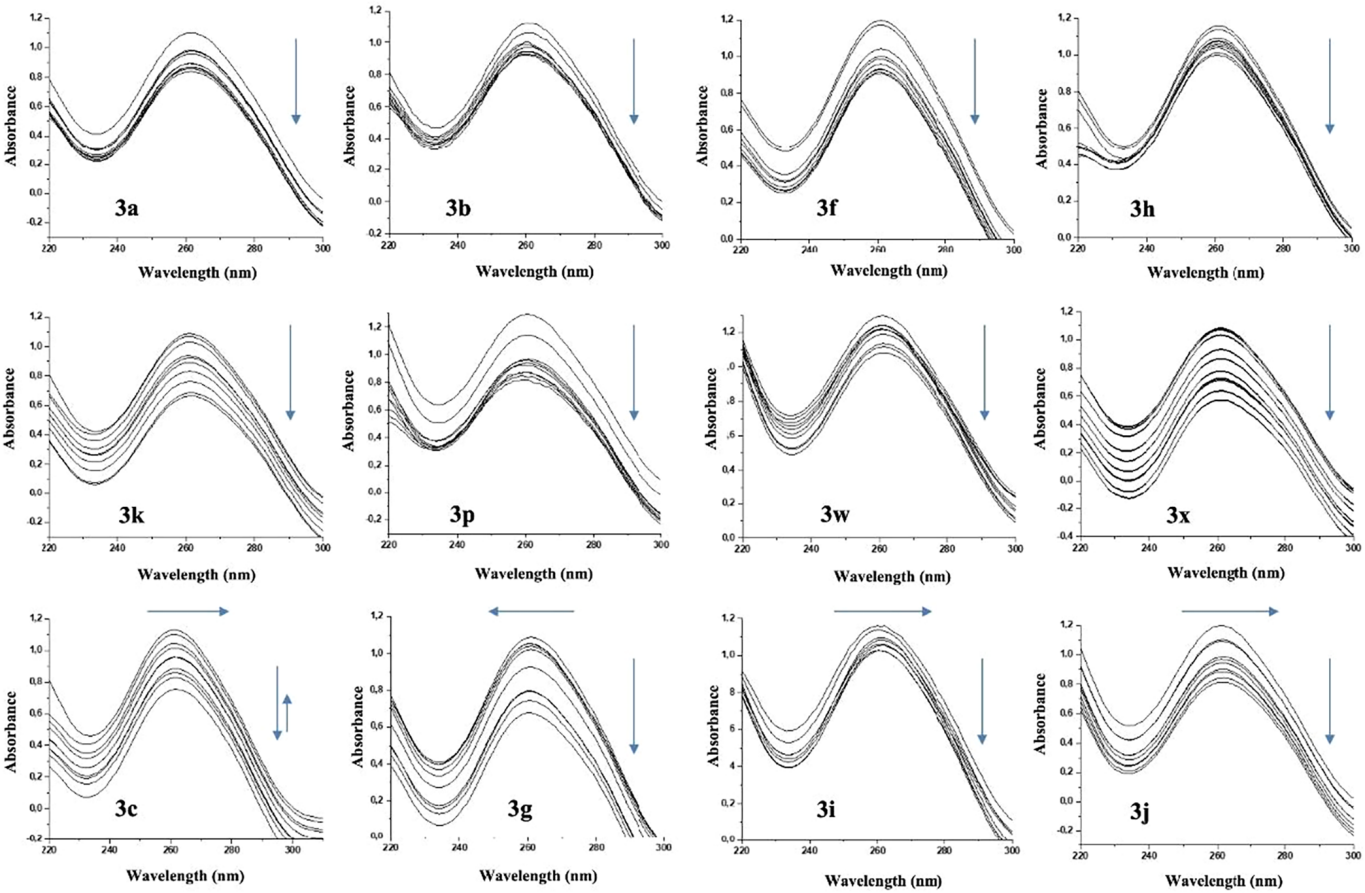
Graphic representation of compound-DNA interactions observed by UV spectrophotometric analysis.

The interactions of compounds **3c**, **3g**, **3i**, and **3j** with DNA reveal distinct binding mechanisms, as evidenced by their spectrophotometric behavior. In the case of compound **3c**, its interaction with DNA varies depending on the concentration. At lower concentrations, **3c** shows hypochromism, characteristic of intercalative binding, where the compound inserts between DNA base pairs. This intercalation stabilizes the DNA helix by enhancing π-π-stacking interactions between the bases. However, at higher concentrations, **3c** transitions to a hyperchromic effect accompanied by a red shift, indicating a change in its binding mode to groove binding. This mode of interaction involves the compound associating with the minor or major grooves of DNA, disrupting the local DNA structure and increasing absorbance, with the red shift reflecting changes in the DNA’s electronic transitions.^62^

Compound **3g** exhibits a pronounced hypochromic effect accompanied by a blue shift, suggesting that its interaction with DNA is primarily mediated by electrostatic forces involving its methoxy group. This interaction likely induces conformational changes in the DNA structure, which can alter the conjugation and rigidity of the molecular system. The hypsochromic shift reflects these structural modifications, leading to a tighter and more compact DNA arrangement.^45,62^

Compound **3j**, on the other hand, shows a strong hypochromic effect coupled with a red shift, indicative of intercalation between DNA base pairs. This interaction stabilizes the DNA helix while perturbing the electronic environment of the bases, as evidenced by bathochromic displacement. Such intercalation results in significant changes to the spectral properties and conformation of DNA, underscoring the compound’s strong binding affinity.

In summary, the spectrophotometric analysis highlights the diverse interactions between the compounds and DNA, ranging from intercalation to electrostatic and groove binding mechanisms. These interactions stabilize the DNA helix and alter its structural and electronic properties, revealing their potential as molecular tools for DNA modulation and as promising candidates for therapeutic applications such as anticancer or gene-targeting agents.

### Molecular Docking Simulation of DNA-Complex Interaction

Molecular docking simulations provided valuable insights into the binding interactions between compound **3** and DNA. The top-ranked docking pose obtained for **3a** (Figure 6A) with BE = −8.0 kcal/mol indicates that such a ligand has the potential to interact via intercalation with DNA. DNA base guanine and cytosine from helix A can form a π-stacking interaction with ligand, from helix B guanine, can form a π–π-stacking and π-anion interaction with ligand, while cytosine just forms a π–anion interaction. Finally, adenine from helix B can interact with the ligand via van der Waals forces (Figure 6B). The surface representation of DNA and **3a** intercalations is shown in Figure 6C.

**Figure 6:**
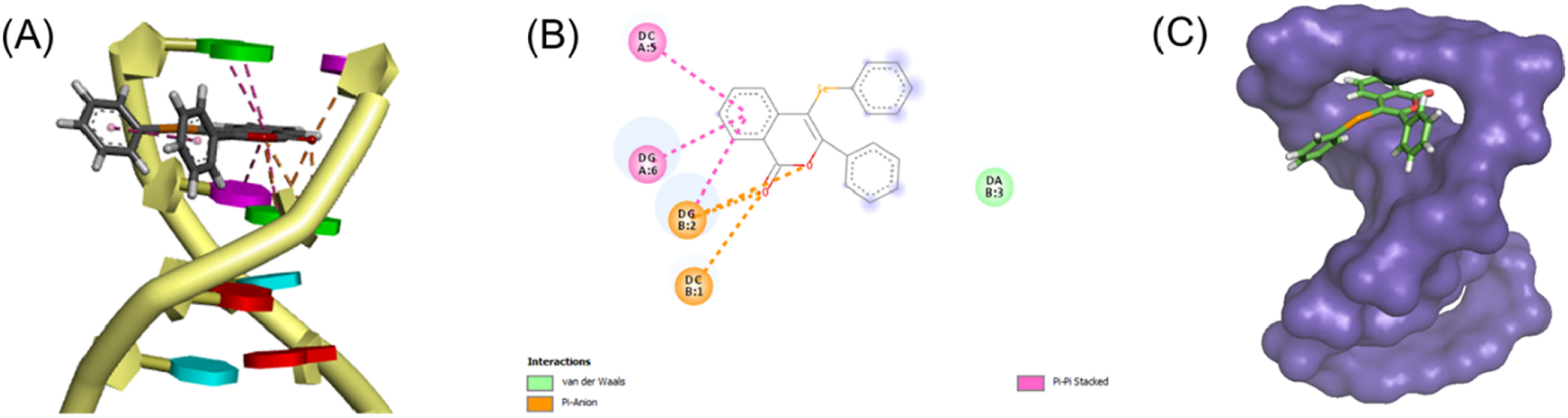
Representation of docking results obtained for **3a** with DNA. (A) 3D (stick) of the best docking pose. (B) 2D (line) of intermolecular interactions. (C) Surface representation of interaction via intercalation (image produced in Pymol).

Docking simulation was applied for all compounds used in the experimental DNA-complex interaction. Compounds **3a**, **3b, 3c**, **3f**, **3g**, **3p**, and **3x** showed the same tendency of **3a** compound to interact via intercalation with DNA, resulting in a binding energy range from −9.0 to −6.9 kcal/mol (Table 5). These predictions corroborate the experimental results for DNA-complex interaction measurements (Figure 5). On the other hand, docking simulations with compounds **3h**, **3i, 3j**, **3k**, and **3w** showed a tendency to interact with DNA through groove binding. It is worth noting that **3h** and **3w** showed a weak hypochromic effect, while **3i** and **3j** showed a strong hypochromic effect coupled with a red shift (Figure 5), which can explain the difficulty of the program in predicting the correct pose of interaction with DNA. Furthermore, compound **3k** was expected to tend to interact via intercalation with DNA—according to the experimental compound-DNA interaction graph—which was not observed in the simulations, probably because the ester group is sterically hindered. Finally, compounds **3h**, **3i, 3j**, **3k**, and **3w** showed the lowest binding energy of the series, varying between −6.4 and −6.3 kcal/mol.

**Table 5:** Binding Energies (kcal/mol) of Best Docking Pose for **3a**, **3b**, **3c**, **3f**, **3g**, **3h**, **3i, 3j**, **3k**, **3p**, **3w**, and **3x**

compound	**3a**	**3b**	**3c**	**3f**	**3g**	**3h**	**3i**	**3j**	**3k**	**3p**	**3w**	**3x**
binding energy (kcal/mol)	–8.0	–6.9	–7.6	–7.8	–8.3	–6.3	–6,4	–6.4	–6.3	–9.0	–6.3	–7.5

## Conclusion

In this study, we established a sustainable and highly selective approach for the synthesis of 4-(phenylselanyl)-isocoumarins, employing trichloroisocyanuric acid (TCCA) as an environmentally benign oxidant. The reaction proceeds under mild conditions, with excellent yields (up to 98%) and no detectable byproducts, underscoring its high selectivity. The protocol showed broad tolerance to both electron-donating and electron-withdrawing substituents, and the synthesis was successfully reproduced on a gram scale, demonstrating its practical applicability. Electrochemical and DFT analyses confirmed the redox stability of the compounds and provided insights into their electronic behavior. DNA interaction studies revealed dual binding modes with enhanced affinity for derivatives bearing electron-withdrawing groups. Overall, this work combines operational simplicity, environmental compatibility, and promising biological potential, offering a valuable approach for the synthesis of relevant selenium-containing heterocycles.

## Supplementary Material



## Notes

Control experiments, general experimental information and procedures, characterization data for all products, cyclic voltammetry studies, and NMR spectra of the synthesized compounds (PDF)

## Abbreviations

BHT: butylated hydroxytoluene
CV: cyclic voltammetry
DFT: density functional theory
DNA: DNA
HOMO: highest occupied molecular orbital
LUMO: lowest unoccupied molecular orbital
TCCA: trichloroisocyanuric acid
TEMPO: 2,2,6,6-tetramethyl-1-piperidinyloxy
TLC: thin-layer chromatography
