# Supplementary material for: Greener Synthesis of 4‑Selanyl-Isocoumarins Mediated by TCCA: Electrochemical Insights and DNA Interaction Studies
Source: ACS Omega. 2025 Oct 31;10(44):52621–33. doi: 10.1021/acsomega.5c06150 (PMC12612971; doi:10.1021/acsomega.5c06150)
Supplement: Supplementary file 1 [file ao5c06150_si_001.pdf]

# Supporting Information

## Greener synthesis of 4-Selanyl-Isocoumarins mediated by TCCA: Electrochemical insights and DNA interaction studies

*Luiz Eduardo Welter,*† Marcelo Baptista,† Celso Rodrigo Nicoleti,† Suzane Quintana Gomes,‡ Fabio S. Miranda,⁑ Suelen Santos da Silva,⁂ Tiago Elias A. Frizon,*‡ and Antonio Luiz Braga*†*

† Department of Chemistry, Federal University of Santa Catarina (UFSC), 88040-970 Florianópolis, SC, Brazil.
‡ Departament of Energy and Sustainability, Federal University of Santa Catarina (UFSC), 88905-120, Araranguá, SC, Brazil.
⁑ Institute of Chemistry, Fluminense Federal University – UFF, 24220-141 Niterói, RJ, Brazil.
⁂ Federal University of Santa Catarina (UFSC), 88905-120, Araranguá, SC, Brazil.

E-mail: luiz.welter@posgrad.ufsc.br, braga.antonio@ufsc.br

**Table of Contents**

Control Experiments ........ 2
General information ........ 2
General Procedures ........ 3
Products Characterization Data ........ 4
Cyclic Voltammetry ........ 13
NMR Spectra ........ 18
References ........ 54

## Control Experiments

To propose a plausible mechanism, control experiments were carried out as shown in the scheme S1.

**Scheme S1**. Control experiments.

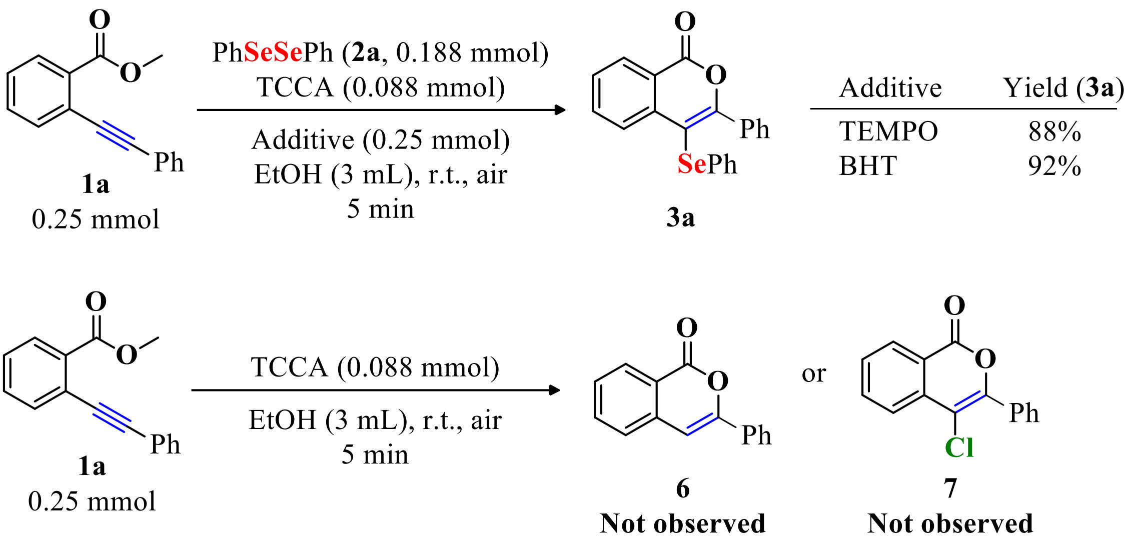


We conducted control experiments using radical scavengers to evaluate the possibility of a radical pathway. When the reaction was carried out in the presence of excess TEMPO or BHT, product **3a** was obtained in 88% and 92% yield, respectively. These results suggest that a radical mechanism is unlikely, and support the involvement of an ionic pathway. Additionally, when the reaction was performed in the absence of diselenide **2a**, no formation of the possible products was observed. Specifically, neither product **6** (the hydrogenated cyclized compound) nor product **7** (the chlorinated cyclized compound), which could result from an alternative cyclization in the absence of selenium, were detected by TLC. These findings reinforce the role of diselenide activation by TCCA in generating the electrophilic selenium species and support a mechanistic sequence in which selenium incorporation precedes cyclization.

## General information

$^{1}$H and $^{13}$C NMR spectra were recorded on Bruker 400 and Bruker AC 200 spectrometer, with the samples dissolved in $CDCl_3$ or *d6*-DMSO. Chemical shifts are reported in in ppm downfield from the signal of TMS, used as internal standard, and the coupling constants (*J*) are expressed in Hertz (Hz). For the $^{77}$Se NMR spectra, the internal standard used was

diphenyl diselenide. Following abbreviations were reported for multiplicity of signal: s (singlet), d (doublet), t (triplet), q (quartet), quint (quintet), sext (sextet) and m (multiplet). High resolution mass spectroscopy was record on Xevo G2-S QTOF (Waterson $ESI^+$ and $ESI^-$ mode). The reactions were monitored by thin layer chromatography (TLC), Macherey-Nagel Silica Gel 818333, 0.20 mm thickness were used. For visualization, UV fluorescence, iodine chamber and acidic methanolic vanillin solution (5% in 10% $H_2SO_4$). Aldrich technical grade silica gel (pore size 60 Å, 230–400 mesh) was used for flash chromatography.

## General Procedures

**General procedure for the synthesis of 2-(alquinil)ésteres 1:**

The starting materials were synthesized using methodologies already described in the literature for classic Sonogashira-type cross coupling reactions[1,2].

**Scheme S2**. Synthesis of substrates **1** by Sonogashira coupling.

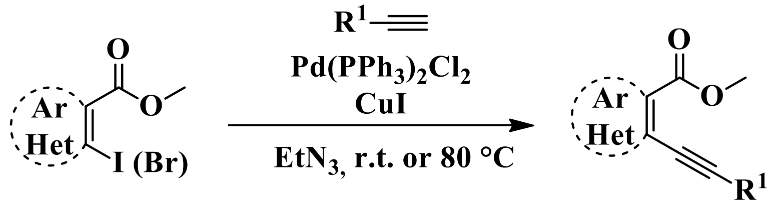


**1a-1o**

Only one of the synthesized starting materials was not previously described: compound **1m** (methyl 2-((6-methoxynaphthalen-1-yl)ethynyl)benzoate).

**General procedure for the synthesis of 4-(selanyl)-1H-isochromen-1-ones 3:** To a glass tube were added TCCA (trichloroisocyanuric acid, 0.0875 mmol), diorganoyl diselenide (0.1875 mmol), and anhydrous ethanol (3.0 mL) at room temperature under stirring for 5 min. After, the starting material 2-(alkynyl)-aryl or –heteroaryl esters (0.25 mmol) were added. The progress of the reaction was monitored by TLC, and the formation of a white precipitate (product) was observed, which interrupted the magnetic stirring. Thus, the reaction was immediately stopped, and the mixture was extracted with ethyl acetate (15 mL) and washed with water (3×15 mL). The organic phase was dried over $MgSO_4$ and concentrated under reduced pressure. The final product was isolated through flash column chromatography using silica gel as the stationary phase and eluted with a mixture of hexane and ethyl acetate.

**Scheme S3**. Synthesis of 4-(selanyl)-1H-isochromen-1-ones **3**.

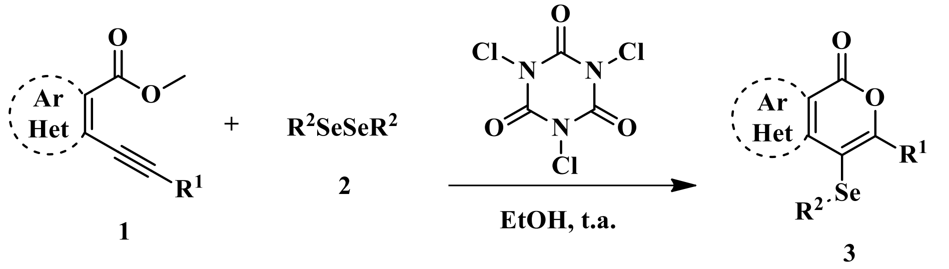


**General procedure for the synthesis of 3-phenyl-4-(phenylseleninyl)-1H-isochromen-1-one 4a:** compound 3a (0.3 mmol), meta-chloroperoxybenzoic acid (0.078 g, 1.5 equivalents) and dichloromethane (5.0 mL) were added to a glass tube at 0 °C under an air atmosphere. The reaction mixture was stirred for 30 minutes. After completion (monitored by TLC), the mixture was extracted with ethyl acetate (15 mL), washed with water (3×15 mL), dried over $MgSO_4$ and concentrated under vacuum. The residue was purified by flash column chromatography on silica gel using a mixture of hexane and ethyl acetate as eluent (50:50), obtaining compound 4a (3-phenyl-4-(phenylseleninyl)-1H-isochromen-1-one) in 93% yield.

**Scheme S4**. Functionalization reaction of 3-phenyl-4-(phenylselanyl)-isocoumarin.

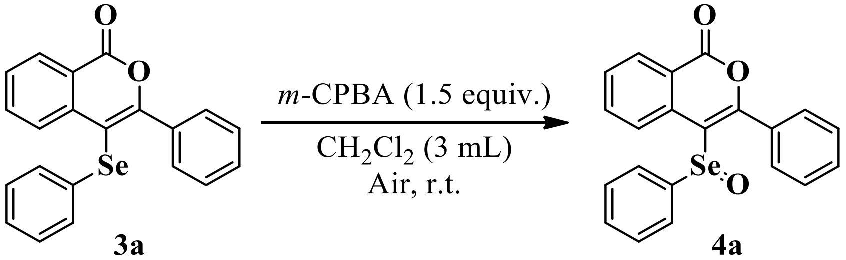


**3a**
0.3 mmol

**4a**
96%, 30 min

**Products Characterization Data**

**methyl 2-((6-methoxynaphthalen-1-yl)ethynyl)benzoate (1m)**

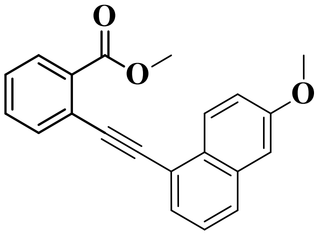


Purified by column chromatography with hexane/ethyl acetate (90:10) as eluents. $R_f$ 0.38 (10% EtOAc in Hex). Yield: 84%. Brown solid, m.p. 81-84 °C. $^1$H NMR ($CDCl_3$, 400 MHz): δ (ppm) 8.02 (s, 1H), 7.99 (dd, $J$ = 7.9, 1.4 Hz, 1H), 7.73 – 7.66 (m, 3H), 7.59 (dd, $J$ = 8.5, 1.7 Hz, 1H), 7.49 (td, $J$ = 7.6, 1.4 Hz, 1H), 7.37 (td, $J$ = 7.7, 1.4 Hz, 1H), 7.16 (dd, $J$ = 8.9, 2.5 Hz, 1H),

7.10 (d, $J$ = 2.5 Hz, 1H), 3.99 (s, 3H), 3.91 (s, 3H). $^{13}$C NMR (101 MHz, $CDCl_3$) δ 166.9, 158.5, 134.4, 134.1, 131.8, 131.8, 131.6, 130.6, 129.5, 129.2, 128.6, 127.8, 127.0, 124.1, 119.5, 118.3, 106.0, 95.2, 88.1, 55.4, 52.3. HRMS (ESI-QToF) calculated for $[M + H]^+$: 317.1172; found: 317.1167.

**3-phenyl-4-(phenylselanyl)-1H-isochromen-1-one (3a)**[3]

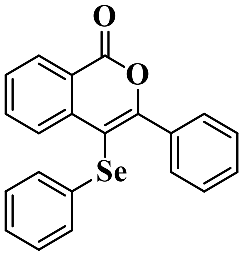


Purified by column chromatography with hexane/ethyl acetate (95:5) as eluents. $R_f$ 0.4 (10% EtOAc in Hex). Yield: 92 mg (97%). White solid, m.p. 125-128 °C (lit. 134−136 °C)[3]. $^1$H NMR ($CDCl_3$, 200 MHz): δ (ppm) 8.36 (dd, $J$ = 7.9, 1.4 Hz, 1H), 8.04 (d, $J$ = 7.8 Hz, 1H), 7.70 (dt, $J$ = 7.9, 1.4 Hz, 1H), 7.66 (dd, $J$ = 8.1, 1.7 Hz, 2H), 7.53 (td, $J$ = 7.7, 1.2 Hz, 1H), 7.47 – 7.35 (m, 3H), 7.24 – 7.11 (m, 5H). $^{13}$C NMR ($CDCl_3$, 101 MHz): δ (ppm) 161.8, 159.7, 138.6, 135.5, 134.2, 132.0, 130.3, 129.8, 129.6, 129.0, 128.8, 128.4, 127.9, 126.9, 126.0, 126.0, 104.9. $^{77}$Se NMR ($CDCl_3$, 76 MHz): δ (ppm) 296.9.

**6,7-dimethyl-3-phenyl-4-(phenylselanyl)-1H-isochromen-1-one (3b)**

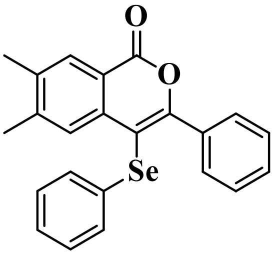


Purified by column chromatography with hexane/ethyl acetate (92:8) as eluents. $R_f$ 0.47 (10% EtOAc in Hex). Yield: 85 mg (93%). White solid, m.p. 202-205 °C. $^1$H NMR ($CDCl_3$, 400 MHz): δ (ppm) 8.11 (s, 1H), 7.80 (s, 1H), 7.66 – 7.61 (m, 2H), 7.43 – 7.35 (m, 3H), 7.22 – 7.14 (m, 5H), 2.38 (s, 3H), 2.33 (s, 3H). $^{13}$C NMR ($CDCl_3$, 101 MHz): δ (ppm) 162.1, 159.0, 145.9, 138.5, 136.6, 134.3, 132.3, 130.1, 130.0, 129.8, 129.6, 128.9, 128.9, 127.8, 126.5, 118.6, 104.7, 20.8, 19.7. $^{77}$Se NMR ($CDCl_3$, 76 MHz): δ (ppm) 294.7. HRMS (ESI-QToF) calculated for $[M + Na]^+$: 429.0364; found: 429.0363.

**7-bromo-3-phenyl-4-(phenylselanyl)-1H-isochromen-1-one (3c)**

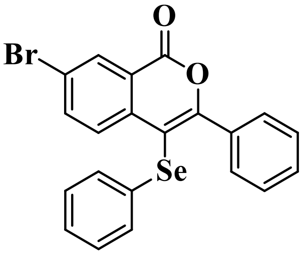


Purified by column chromatography with hexane/ethyl acetate (95:5) as eluents. $R_f$ 0.62 (10% EtOAc in Hex). Yield: 104 mg (91%). White solid, m.p. 143-146 °C. $^1$H NMR ($CDCl_3$, 400 MHz): δ (ppm) 8.48 (d, $J$ = 2.1 Hz, 1H), 7.91 (d, $J$ = 8.7 Hz, 1H), 7.77 (dd, $J$ = 8.7, 2.2 Hz, 1H), 7.68 – 7.64 (m, 2H), 7.48

– 7.38 (m, 3H), 7.22 – 7.15 (m, 5H). $^{13}C$ NMR ($CDCl_3$, 101 MHz): δ (ppm) 160.6, 159.9, 138.5, 137.5, 133.8, 132.3, 131.5, 130.5, 130.3, 129.8, 129.7, 129.0, 128.0, 126.9, 122.7, 122.3, 104.4. $^{77}Se$ NMR ($CDCl_3$, 76 MHz): δ (ppm) 298.9. HRMS (ESI-QToF) calculated for $[M + H]^+$: 478.9156; found: 478.9157.

**5-phenyl-4-(phenylselanyl)-7H-thieno[2,3-c]pyran-7-one (3f)**

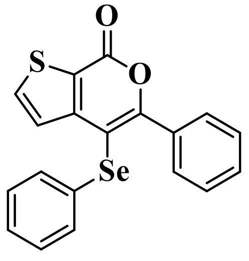

Purified by column chromatography with hexane/ethyl acetate (95:5) as eluents. $R_f$ 0.30 (10% EtOAc in Hex). Yield: 74 mg (77%). White solid, m.p. 140-143 °C. $^1H$ NMR (DMSO-$d_6$, 400 MHz): δ (ppm) 8.48 (d, $J$ = 2.1 Hz, 1H), 7.91 (d, $J$ = 8.7 Hz, 1H), 7.77 (dd, $J$ = 8.7, 2.2 Hz, 1H), 7.68 – 7.64 (m, 2H), 7.48 – 7.38 (m, 3H), 7.22 – 7.15 (m, 5H). $^{13}C$ NMR (DMSO-$d_6$, 101 MHz): δ (ppm) 160.6, 157.0, 150.1, 150.0, 138.5, 133.0, 131.1, 130.3, 129.7, 129.6, 129.0, 128.0, 127.2, 126.7, 122.0, 101.0. $^{77}Se$ NMR ($CDCl_3$, 76 MHz): δ (ppm) 324.5. HRMS (ESI-QToF) calculated for $[M + Na]^+$: 406.9615; found: 406.9613.

**3-(4-methoxyphenyl)-4-(phenylselanyl)-1H-isochromen-1-one (3g)**[3]

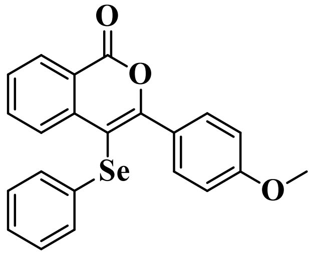

Purified by column chromatography with hexane/ethyl acetate (93:7) as eluents. $R_f$ 0.37 (10% EtOAc in Hex). Yield: 82 mg (80%). White solid, m.p. 149-152 °C (lit. 155−158 °C)[3]. $^1H$ NMR ($CDCl_3$, 400 MHz): δ (ppm) 8.35 (dd, J = 7.9, 1.3 Hz, 1H), 8.02 (d, J = 7.9 Hz, 1H), 7.69 (td, $J$ = 8.0, 1.3 Hz, 1H), 7.65 (d, $J$ = 8.9 Hz, 2H), 7.51 (t, J = 7.9 Hz, 1H), 7.22 – 7.14 (m, 5H), 6.91 (d, J = 8.9 Hz, 2H), 3.84 (s, 3H). $^{13}C$ NMR ($CDCl_3$, 101 MHz): δ (ppm) 161.8, 161.0, 159.5, 138.7, 135.3, 132.1, 131.4, 129.6, 129.5, 128.7, 128.4, 128.2, 126.4, 126.4, 120.7, 113.2, 103.8, 55.4. $^{77}Se$ NMR ($CDCl_3$, 76 MHz): δ (ppm) 298.8.

**4-(phenylselanyl)-3-(p-tolyl)-1H-isochromen-1-one (3h)**[4]

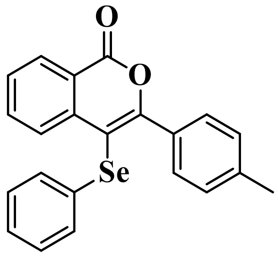

Purified by column chromatography with hexane/ethyl acetate (95:5) as eluents. $R_f$ 0.40 (10% EtOAc in Hex). Yield: 88 mg (90%). White solid, m.p. 112-115 °C[4]. $^1H$ NMR ($CDCl_3$, 400 MHz): δ (ppm) 8.35 (dd, $J$ = 7.9, 1.5 Hz, 1H), 8.02 (dd, $J$ = 8.1, 1.0 Hz, 1H), 7.68 (td, $J$ = 8.2, 7.7, 1.5 Hz, 1H), 7.57 (d, $J$ = 8.2 Hz, 2H), 7.51 (td, $J$ = 7.6, 1.1 Hz, 1H), 7.22 – 7.14 (m, 8H), 2.39 (s, 3H). $^{13}C$ NMR

(CDCl3, 101 MHz): δ (ppm) 161.9, 159.9, 140.6, 138.7, 135.4, 132.1, 131.3, 129.8, 129.8, 129.6, 128.8, 128.7, 128.6, 128.3, 126.5, 120.9, 104.4, 21.6. $^{77}$Se NMR (CDCl3, 76 MHz): δ (ppm) 297.7.

**3-(4-chlorophenyl)-4-(phenylselanyl)-1H-isochromen-1-one (3i)**[3]

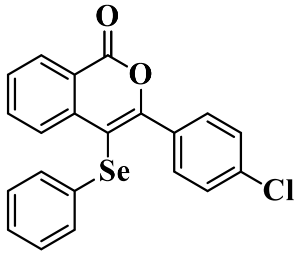


Purified by column chromatography with hexane/ethyl acetate (95:5) as eluents. $R_f$ 0.48 (10% EtOAc in Hex). Yield: 85 mg (82%). White solid, m.p. 110-113 °C (lit.105−108 °C)[3]. $^1$H NMR (CDCl3, 400 MHz): δ (ppm) 8.34 (dd, J = 7.9, 1.4Hz, 1H), 8.04 (d, J = 8.0 Hz, 1H), 7.70 (td, J = 7.9, 1.3 Hz, 1H), 7.60 (d, J = 8.6 Hz, 2H), 7.53 (td, J = 7.6, 1.3 Hz, 1H), 7.35 (d, J = 8.6 Hz, 1H), 7.20-7.15 (m, 5H). $^{13}$C NMR (CDCl3, 101 MHz): δ (ppm) 161.5, 158.4, 138.3, 136.3, 135.5, 132.5, 131.7, 131.1, 129.8, 129.7, 129.0, 128.9, 128.4, 128.2, 126.7, 120.9, 105.2. $^{77}$Se NMR (CDCl3, 76 MHz): δ (ppm) 296.5.

**4-(phenylselanyl)-3-(p-tolyl)-1H-isochromen-1-one (3j)**[5]

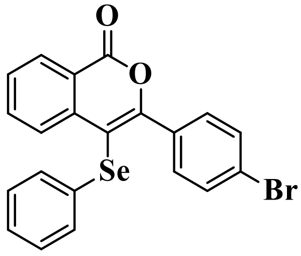


Purified by column chromatography with hexane/ethyl acetate (95:5) as eluents. $R_f$ 0.42 (10% EtOAc in Hex). Yield: 88 mg (90%). White solid, m.p. 139-142 °C[5]. $^1$H NMR (CDCl3, 400 MHz): δ (ppm) 8.36 (dd, *J* = 7.9, 1.4 Hz, 1H), 8.05 (d, *J* = 8.6 Hz, 1H), 7.72 (ddd, *J* = 8.4, 7.3, 1.5 Hz, 1H), 7.57 – 7.52 (m, 6H), 7.22 – 7.16 (m, 6H). $^{13}$C NMR (CDCl3, 101 MHz): δ (ppm) 161.9, 159.9, 140.6, 138.7, 135.4, 132.1, 131.3, 129.8, 129.8, 129.6, 128.8, 128.7, 128.6, 128.3, 126.5, 120.9, 104.4, 21.6. $^{77}$Se NMR (CDCl3, 76 MHz): δ (ppm) 296.6.

**methyl 4-(1-oxo-4-(phenylselanyl)-1H-isochromen-3-yl)benzoate (3k)**

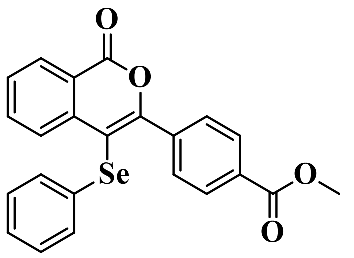


Purified by column chromatography with hexane/ethyl acetate (90:10) as eluents. $R_f$ 0.20 (10% EtOAc in Hex). Yield: 66 mg (60%). White solid, m.p. 157-160 °C. $^1$H NMR (CDCl3, 400 MHz): δ (ppm) 8.37 (dd, *J* = 7.9, 1.5 Hz, 1H), 8.11 – 8.02 (m, 3H), 7.78 – 7.69 (m, 3H), 7.61 – 7.52 (m, 1H), 7.22 – 7.15 (m, 5H), 3.93 (s, 3H). $^{13}$C NMR (CDCl3, 101 MHz): δ (ppm) 166.5, 161.5, 158.5, 138.3, 138.2, 135.6, 131.6, 131.5, 129.9, 129.9, 129.7, 129.2, 129.1, 129.0,

128.5, 126.8, 121.0, 105.9, 52.4. $^{77}$Se NMR ($CDCl_3$, 76 MHz): δ (ppm) 295.8. HRMS (ESI-QToF) calculated for $[M + H]^+$: 437.0287; found: 437.0285.

**4-(phenylselanyl)-3-(thiophen-2-yl)-1H-isochromen-1-one (3l)**[5]

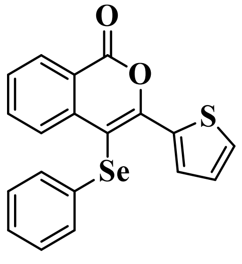

Purified by column chromatography with hexane/ethyl acetate (92:8) as eluents. $R_f$ 0.40 (10% EtOAc in Hex). Yield: 90 mg (94%). White solid, m.p. 141-144 °C[5]. $^1$H NMR ($CDCl_3$, 400 MHz): δ (ppm) 8.34 (dd, $J$ = 7.9, 1.4 Hz, 1H), 8.08 (dd, $J$ = 8.3, 1.0 Hz, 1H), 8.05 – 7.99 (m, 1H), 7.75 – 7.64 (m, 2H), 7.52 (td, $J$ = 7.6, 1.1 Hz, 1H), 7.33 – 7.31 (m, 1H), 7.27 – 7.17 (m, 5H). $^{13}$C NMR ($CDCl_3$, 101 MHz): δ (ppm) 161.7, 154.9, 139.0, 135.5, 134.5, 131.7, 129.8, 129.6, 128.8, 128.7, 128.4, 126.7, 125.0, 120.8, 103.7. $^{77}$Se NMR ($CDCl_3$, 76 MHz): δ (ppm) 291.5.

**3-(6-methoxynaphthalen-1-yl)-4-(phenylselanyl)-1H-isochromen-1-one (3m)**

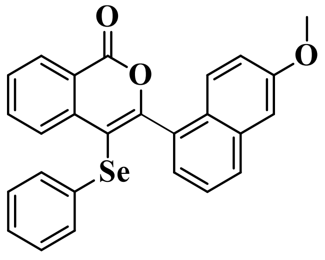

Purified by column chromatography with hexane/ethyl acetate (90:10) as eluents. $R_f$ 0.30 (10% EtOAc in Hex). Yield: 81 mg (70%). White solid, m.p. 162-165 °C. $^1$H NMR ($CDCl_3$, 400 MHz): δ (ppm) 8.38 (dd, $J$ = 7.8, 1.4 Hz, 1H), 8.13 – 8.04 (m, 2H), 7.77 – 7.67 (m, 4H), 7.54 (ddd, $J$ = 8.2, 6.1, 1.1 Hz, 1H), 7.24 – 7.13 (m, 7H), 3.93 (s, 3H). $^{13}$C NMR ($CDCl_3$, 101 MHz): δ (ppm) 162.0, 159.9, 159.0, 138.8, 135.5, 135.4, 132.2, 130.4, 130.1, 129.8, 129.7, 129.2, 129.0, 128.7, 128.4, 127.9, 127.2, 126.6, 126.2, 120.9, 119.6, 105.8, 104.7, 55.5. $^{77}$Se NMR ($CDCl_3$, 76 MHz): δ (ppm) 295.4. HRMS (ESI-QToF) calculated for $[M + Na]^+$: 481.0313; found: 481.0315.

**4-((4-methoxyphenyl)selanyl)-3-phenyl-1H-isochromen-1-one (3n)**[3]

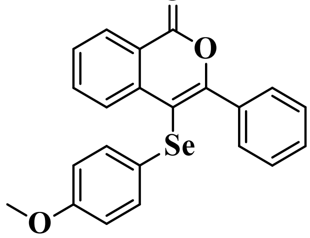

Purified by column chromatography with hexane/ethyl acetate (93:7) as eluents. $R_f$ 0.26 (10% EtOAc in Hex). Yield: 95 mg (93%). White solid, m.p. 93-96 °C (lit. 94–97°C)[3]. $^1$H NMR ($CDCl_3$, 400 MHz): δ (ppm) 8.33 (d, J = 7.8 Hz, 1H), 8.11 (d, J = 8.2 Hz, 1H), 7.72 – 7.65 (m, 3H), 7.53 – 7.38 (m, 4H), 7.12 (d, J = 8.8 Hz, 2H), 6.71 (d, J = 8.8 Hz, 2H), 3.70 (s, 3H). $^{13}$C NMR ($CDCl_3$, 101 MHz): δ (ppm) 161.8, 159.1, 159.0, 138.6, 135.3, 134.9, 131.6, 130.1, 129.9, 129.7, 128.7, 128.4, 127.9, 121.5, 120.9, 115.3, 106.1, 55.3. $^{77}$Se NMR ($CDCl_3$, 76 MHz): δ (ppm) 281.2.

**4-((2-methoxyphenyl)selanyl)-3-phenyl-1H-isochromen-1-one (3o)**

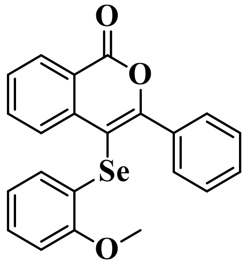

Purified by column chromatography with hexane/ethyl acetate (90:10) as eluents. $R_f$ 0.27 (10% EtOAc in Hex). Yield: 91 mg (89%). White solid, m.p. 179-182 °C. $^1$H NMR ($CDCl_3$, 400 MHz): δ (ppm) 8.37 (dd, $J$ = 7.9, 1.4 Hz, 1H), 7.99 – 7.95 (m, 1H), 7.71 – 7.64 (m, 3H), 7.53 (ddd, $J$ = 8.3, 7.3, 1.2 Hz, 1H), 7.43 – 7.33 (m, 3H), 7.16 (ddd, $J$ = 8.2, 7.3, 1.7 Hz, 1H), 6.86 – 6.82 (m, 2H), 6.76 (td, $J$ = 7.5, 1.2 Hz, 1H), 3.88 (s, 3H). $^{13}$C NMR ($CDCl_3$, 101 MHz): δ (ppm) 162.0, 159.9, 156.3, 138.7, 135.5, 134.1, 130.2, 129.7, 129.6, 128.8, 128.5, 128.1, 127.9, 127.2, 122.1, 121.4, 120.9, 110.5, 102.9, 56.0. $^{77}$Se NMR ($CDCl_3$, 76 MHz): δ (ppm) 264.5. HRMS (ESI-QToF) calculated for $[M + Na]^+$: 431.0157; found: 431.0156.

**3-phenyl-4-(p-tolylselanyl)-1H-isochromen-1-one (3p)**[3]

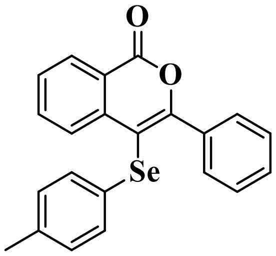

Purified by column chromatography with hexane/ethyl acetate (95:5) as eluents. $R_f$ 0.51 (10% EtOAc in Hex). Yield: 93 mg (95%). White solid, m.p. 162-165 °C (lit. 170−173 °C)[3]. $^1$H NMR ($CDCl_3$, 400 MHz): δ (ppm) 8.36 (dd, $J$ = 7.9, 1.4 Hz, 1H), 8.07 (d, $J$ = 8.6 Hz, 1H), 7.71 (ddd, $J$ = 8.4, 7.3, 1.4 Hz, 1H), 7.67 (dd, $J$ = 8.0, 1.7 Hz, 2H), 7.53 (td, $J$ = 7.6, 1.2 Hz, 1H), 7.45 – 7.38 (m, 3H), 7.09 (d, $J$ = 8.1 Hz, 2H), 7.00 (d, $J$ = 7.9 Hz, 2H), 2.26 (3H). $^{13}$C NMR ($CDCl_3$, 101 MHz): δ (ppm) 159.5, 138.7, 137.4, 136.6, 135.5, 134.2, 130.4, 130.2, 129.9, 129.8, 129.3, 128.8, 128.5, 128.1, 127.9, 121.0, 105.2, 77.2, 21.1. $^{77}$Se NMR ($CDCl_3$, 76 MHz): δ (ppm) 289.9.

**4-((4-fluorophenyl)selanyl)-3-phenyl-1H-isochromen-1-one (3q)**[3]

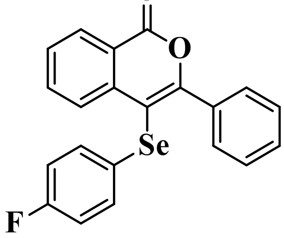

Purified by column chromatography with hexane/ethyl acetate (95:5) as eluents. $R_f$ 0.52 (10% EtOAc in Hex). Yield: 95 mg (96%). White solid, m.p. 134-137 °C (lit. 137−140 °C)[3]. $^1$H NMR ($CDCl_3$, 400 MHz): δ (ppm) 8.36 (dd, $J$ = 8.0, 1.4 Hz, 1H), 8.05 (d, $J$ = 7.7 Hz, 1H), 7.73 (td, $J$ = 7.8, 1.4 Hz, 1H), 7.64 (dd, $J$ = 8.1, 1.4 Hz, 2H), 7.55 (td, $J$ = 7.7, 1.1 Hz, 1H), 7.50 – 7.38 (m, 3H), 7.21 – 7.11 (m, 2H), 6.94 – 6.85 (m, 2H). $^{13}$C NMR ($CDCl_3$, 101 MHz): δ (ppm) 162.0 (d, $J$ = 247.3 Hz), 161.7, 159.6, 138.4, 135.5, 134.1, 131.4 (d, $J$ = 7.7 Hz), 130.3, 130.0, 129.9, 128.9, 128.2,

128.0, 126.1 (d, $J$ = 3.3 Hz), 121.0, 116.8 (d, $J$ = 21.9 Hz), 105.5. $^{77}$Se NMR ($CDCl_3$, 76 MHz): δ (ppm) 291.1. $^{19}$F NMR (376 MHz, $CDCl_3$) δ -115.2.

**4-((4-chlorophenyl)selanyl)-3-phenyl-1H-isochromen-1-one (3r)**[3]

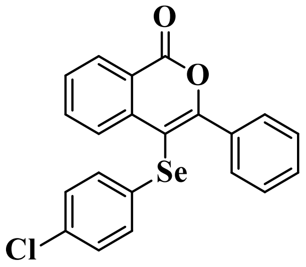


Purified by column chromatography with hexane/ethyl acetate (95:5) as eluents. $R_f$ 0.51 (10% EtOAc in Hex). Yield: 92 mg (89%). White solid, m.p. 137-140 °C (lit. 139−142 °C)[3]. $^1$H NMR ($CDCl_3$, 400 MHz): δ (ppm) 8.36 (dd, J = 7.9, 1.4 Hz, 1H), 7.99 (d, J = 9.0 Hz, 1H), 7.72 (td, J = 7.9, 1.4 Hz, 1H), 7.63 (dd, J = 8.2, 1.4Hz, 2H), 7.55 (td, J = 7.6, 1.1 Hz, 1H), 7.47 – 7.38 (m, 3H), 7.13 (q, J = 8.7 Hz, 4H). $^{13}$C NMR ($CDCl_3$, 101 MHz): δ (ppm) 161.6, 159.9, 138.2, 135.6, 133.9, 132.8, 130.4, 130.3, 130.1, 130.0, 129.8, 129.0, 128.1, 128.0, 121.0, 104.7. $^{77}$Se NMR ($CDCl_3$, 76 MHz): δ (ppm) 297.5.

**3-phenyl-4-((3-(trifluoromethyl)phenyl)selanyl)-1H-isochromen-1-one (3s)**[3]

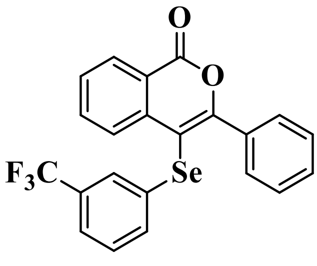


Purified by column chromatography with hexane/ethyl acetate (95:5) as eluents. $R_f$ 0.46 (10% EtOAc in Hex). Yield: 102 mg (92%). White solid, m.p. 84-87 °C (lit. 81−83 °C)[3]. $^1$H NMR ($CDCl_3$, 400 MHz): δ (ppm) 8.39 (dd, $J$ = 8.0, 1.4 Hz, 1H), 8.00 (d, $J$ = 8.1 Hz, 1H), 7.75 (td, $J$ = 7.8, 1.5 Hz, 1H), 7.65 – 7.56 (m, 2H), 7.47 – 7.39 (m, 5H), 7.31 – 7.28 (m, 3H). $^{13}$C NMR ($CDCl_3$, 101 MHz): δ (ppm) 161.6, 160.3, 138.1, 135.7, 133.9, 133.1, 132.2, 131.8 ($J$ = 33.7 Hz), 130.5, 130.1, 130.0, 129.7, 129.1, 128.1, 128.0, 125.8 ($J$ = 4.2 Hz), 123.7 ($J$ = 273.1 Hz), 123.6 ($J$ = 3.6 Hz), 121.0, 104.5. $^{77}$Se NMR ($CDCl_3$, 76 MHz): δ (ppm) 306.5. $^{19}$F NMR (376 MHz, $CDCl_3$) δ -62.8.

**4-(benzylselanyl)-3-phenyl-1H-isochromen-1-one (3t)**

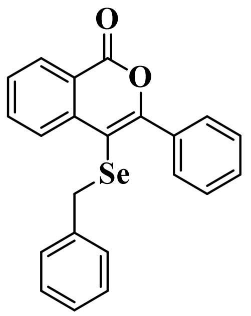


Purified by column chromatography with hexane/ethyl acetate (95:5) as eluents. $R_f$ 0.48 (10% EtOAc in Hex). Yiel d: 91 mg (93%). White solid, m.p. 132-135 °C. $^1$H NMR ($CDCl_3$, 400 MHz): δ (ppm) 8.35 (dd, $J$ = 7.8, 1.4 Hz, 1H), 8.20 (d, $J$ = 8.6 Hz, 1H), 7.82 (ddd, $J$ = 8.4, 7.3, 1.5 Hz, 1H), 7.57 (td, $J$ = 8.1, 1.1 Hz, 1H), 7.43 – 7.30 (m, 5H), 7.15 – 7.04 (m, 3H), 6.81 (dd, $J$ = 7.7, 1.8 Hz, 2H), 3.75 (s, 1H). $^{13}$C NMR ($CDCl_3$, 101 MHz): δ (ppm) 161.9, 159.3, 138.9, 137.6,

135.3, 134.2, 130.2, 130.0, 129.8, 128.9, 128.6, 128.5, 128.2, 127.6, 127.1, 120.8, 105.0, 32.1. $^{77}$Se NMR (CDCl$_3$, 76 MHz): δ (ppm) 255.3 (t, $J$ = 15.2 Hz). HRMS (ESI-QToF) calculated for [M + Na]$^+$: 415.0208; found: 415.0209.

**4-(naphthalen-1-ylselanyl)-3-phenyl-1H-isochromen-1-one (3u)**

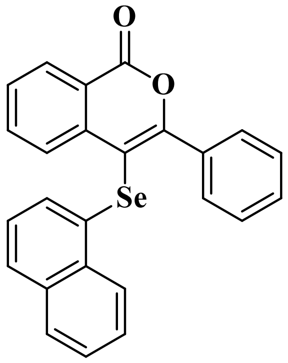

Purified by column chromatography with hexane/ethyl acetate (90:10) as eluents. R$_f$ 0.47 (10% EtOAc in Hex). Yield: 81 mg (90%). White solid, m.p. 198-201. $^1$H NMR (CDCl$_3$, 400 MHz): δ (ppm) 8.37 (dd, $J$ = 7.9, 1.4 Hz, 1H), 8.06 – 7.92 (m, 2H), 7.92 – 7.80 (m, 1H), 7.76 – 7.66 (m, 3H), 7.63 (td, $J$ = 8.8, 8.2, 1.4 Hz, 1H), 7.58 – 7.45 (m, 3H), 7.48 – 7.29 (m, 3H), 7.23 (d, $J$ = 14.5 Hz, 2H). $^{13}$C NMR (CDCl$_3$, 101 MHz): δ (ppm) 161.9, 159.9, 138.5, 135.6, 134.2, 134.0, 132.2, 130.8, 130.4, 129.9, 129.7, 128.9, 128.8, 128.4, 128.0, 127.1, 126.7, 126.7, 126.6, 126.4, 125.5, 121.0, 104.2. $^{77}$Se NMR (CDCl$_3$, 76 MHz): δ (ppm) 266.4. HRMS (ESI-QToF) calculated for [M + Na]$^+$: 451.0208; found: 451.0207.

**4-(butylselanyl)-3-phenyl-1H-isochromen-1-one (3v)[3]**

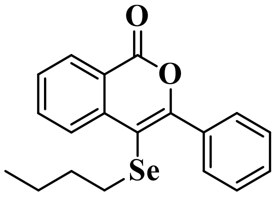

Purified by column chromatography with hexane/ethyl acetate (95:5) as eluents. R$_f$ 0.72 (10% EtOAc in Hex). Yield: 68 mg (76%). White solid, m.p. 59-62 °C (lit. 62−64 °C)[3]. $^1$H NMR (CDCl$_3$, 400 MHz): δ (ppm) 8.36 (dd, $J$ = 7.8, 1.4 Hz, 1H), 8.26 (dd, $J$ = 8.2, 1.0 Hz, 1H), 7.84 (td, $J$ = 8.2, 7.8, 1.5 Hz, 1H), 7.74 – 7.66 (m, 2H), 7.57 (td, $J$ = 7.6, 1.1 Hz, 1H), 7.50 – 7.41 (m, 3H), 2.55 (t, $J$ = 7.3 Hz, 2H), 1.40 (p, $J$ = 7.4 Hz, 2H), 1.17 (h, $J$ = 14.5, 7.2 Hz, 2H), 0.74 (t, $J$ = 7.3 Hz, 3H). $^{13}$C NMR (CDCl$_3$, 101 MHz): δ (ppm) 162.1, 158.1, 139.1, 135.3, 134.5, 130.3, 129.9, 129.9, 128.6, 128.3, 127.8, 120.9, 105.2, 31.7, 28.9, 22.7, 13.5. $^{77}$Se NMR (CDCl$_3$, 76 MHz): δ (ppm) 160.4 (p, $J$ = 21.9, 11.4 Hz).

**3-(4-methoxyphenyl)-4-((4-methoxyphenyl)selanyl)-1H-isochromen-1-one (3w)**

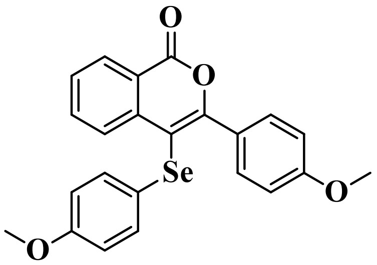


Purified by column chromatography with hexane/ethyl acetate (90:10) as eluents. $R_f$ 0.23 (10% EtOAc in Hex). Yield: 104 mg (95%). White solid, m.p. 136-139 °C. $^1H$ NMR ($CDCl_3$, 400 MHz): δ (ppm) 8.32 (dd, *J* = 7.9, 1.4 Hz, 1H), 8.09 (d, *J* = 9.3 Hz, 1H), 7.70 (td, *J* = 8.2, 7.8, 1.4 Hz, 1H), 7.66 (d, *J* = 8.8 Hz, 2H), 7.50 (td, *J* = 7.6, 1.1 Hz, 1H), 7.14 (d, *J* = 8.8 Hz, 2H), 6.93 (d, *J* = 8.8 Hz, 2H), 6.73 (d, *J* = 8.7 Hz, 2H), 3.85 (s, 3H), 3.73 (s, 3H). $^{13}C$ NMR ($CDCl_3$, 101 MHz): δ (ppm) 162.1, 161.1, 159.1, 159.0, 138.9, 135.3, 131.7, 131.4, 129.7, 128.4, 128.4, 126.6, 121.8, 120.9, 115.3, 113.3, 105.1, 55.5, 55.4. $^{77}Se$ NMR ($CDCl_3$, 76 MHz): δ (ppm) 282.5. HRMS (ESI-QToF) calculated for $[M + Na]^+$: 461.0263; found: 461.0265.

**4-((4-chlorophenyl)selanyl)-3-(4-methoxyphenyl)-1H-isochromen-1-one (3x)**

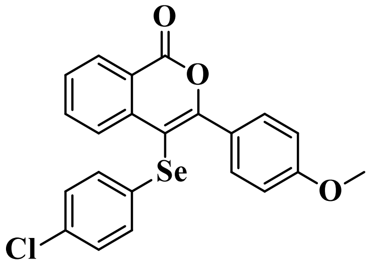


Purified by column chromatography with hexane/ethyl acetate (90:10) as eluents. $R_f$ 0.37 (10% EtOAc in Hex). Yield: 81 mg (73%). White solid, m.p. 185-188 °C. $^1H$ NMR ($CDCl_3$, 400 MHz): δ (ppm) 8.35 (dd, *J* = 7.9, 1.4 Hz, 1H), 7.97 (d, *J* = 8.1 Hz, 1H), 7.71 (ddd, *J* = 8.2, 7.3, 1.5 Hz, 1H), 7.62 (d, *J* = 8.8 Hz, 2H), 7.53 (ddd, *J* = 8.2, 7.3, 1.1 Hz, 1H), 7.14 (qd, *J* = 8.7, 0.2 Hz, 4H), 6.92 (d, *J* = 8.8 Hz, 2H), 3.85 (s, 3H). $^{13}C$ NMR ($CDCl_3$, 101 MHz): δ (ppm) 161.9, 161.3, 159.9, 138.6, 135.6, 132.7, 131.5, 130.3, 130.8, 129.9, 129.8, 128.7, 128.1, 126.3, 120.8, 113.4, 103.7, 55.5. $^{77}Se$ NMR ($CDCl_3$, 76 MHz): δ (ppm) 299.3. HRMS (ESI-QToF) calculated for $[M + Na]^+$: 464.9767; found: 464.9767.

**3-phenyl-4-(phenylseleninyl)-1H-isochromen-1-one (4a)**

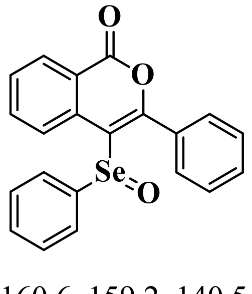


Purified by column chromatography with hexane/ethyl acetate (50:50) as eluents. $R_f$ 0.22 (100% EtOAc). Yield: 113 mg (93%). White solid, m.p. 99-102 °C. $^1H$ NMR ($CDCl_3$, 400 MHz): δ (ppm) 8.25 (d, *J* = 8.1 Hz, 2H), 7.71 (t, *J* = 6.2, 5.7 Hz, 4H), 7.58 – 7.50 (m, 4H), 7.48 – 7.41 (m, 4H). $^{13}C$ NMR ($CDCl_3$, 101 MHz): δ (ppm) 160.6, 159.2, 140.5, 135.1, 134.1, 131.7, 131.2, 130.1, 129.9, 129.9, 129.3, 129.1, 126.7, 125.0, 121.2, 116.2, 77.4. $^{77}Se$ NMR ($CDCl_3$, 76 MHz): δ (ppm) 902.5. HRMS (ESI-QToF) calculated for $[M + Na]^+$: 417.0000; found: 417.0003.

## Cyclic Voltammetry

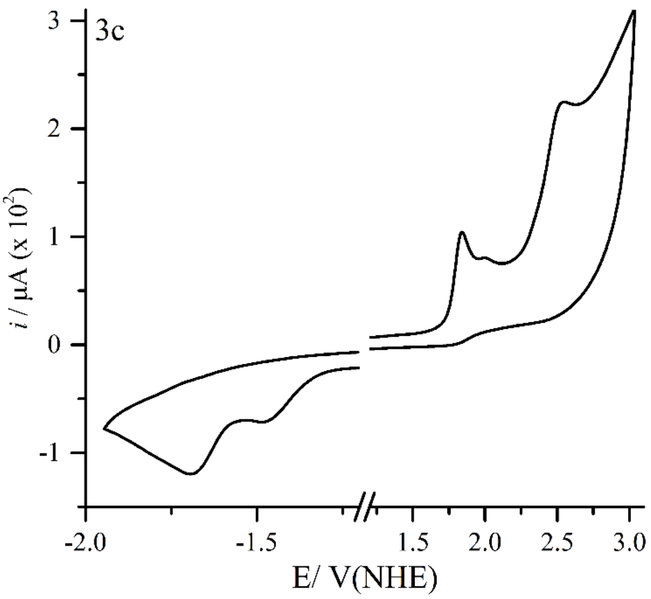


**Figure S1.** Cyclic voltammetry of **3c** in acetonitrile at 100 mV $s^{-1}$.

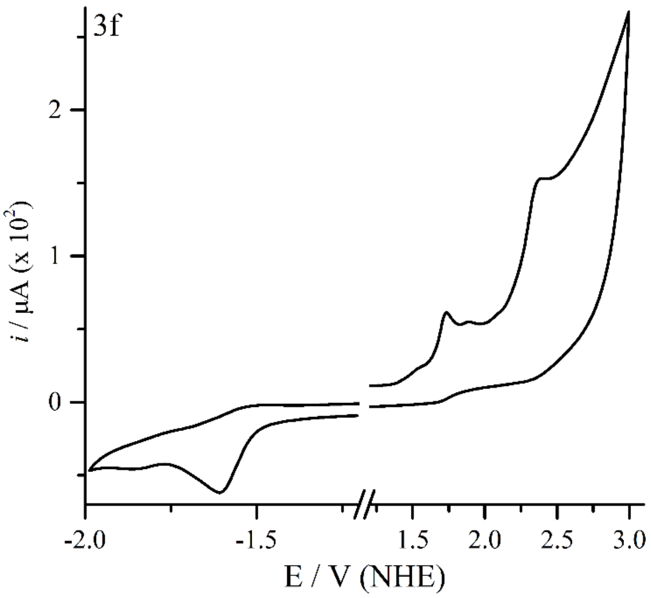


**Figure S2.** Cyclic voltammetry of **3f** in acetonitrile at 100 mV $s^{-1}$.

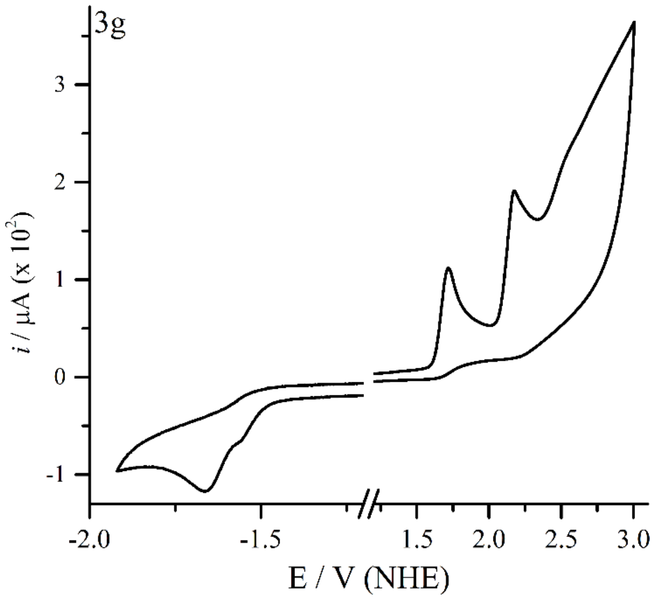


**Figure S3.** Cyclic voltammetry of **3g** in acetonitrile at 100 mV s$^{-1}$.

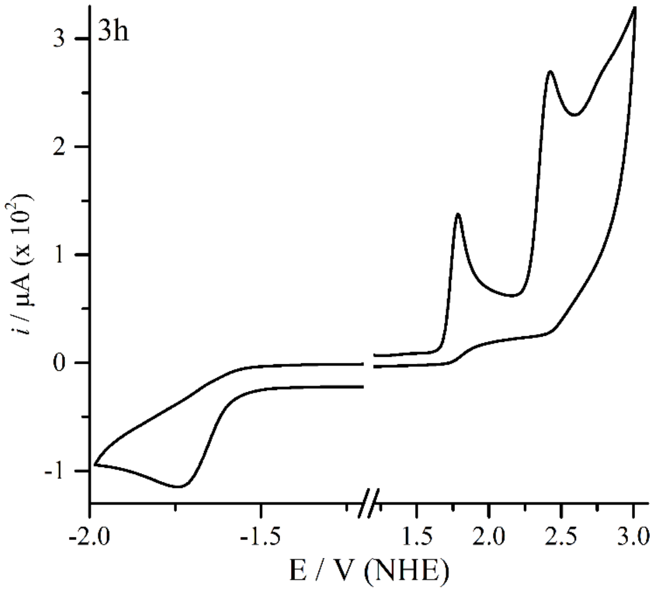


**Figure S4.** Cyclic voltammetry of **3h** in acetonitrile at 100 mV s$^{-1}$.

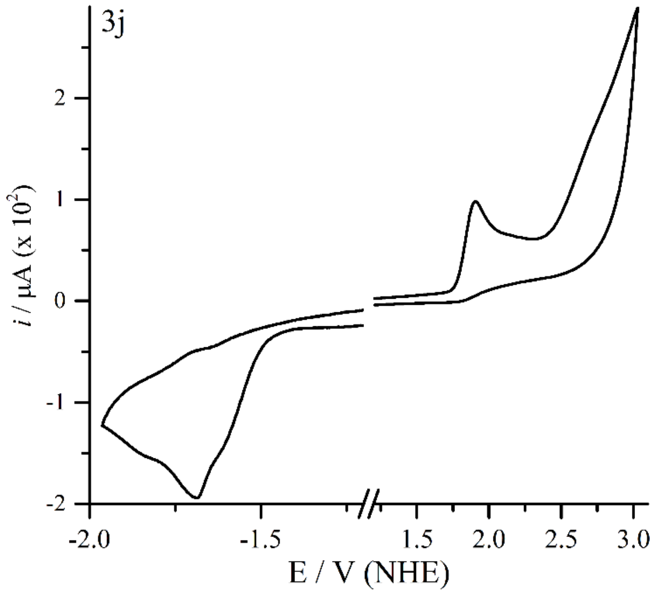


**Figure S5.** Cyclic voltammetry of **3j** in acetonitrile at 100 mV $s^{-1}$.

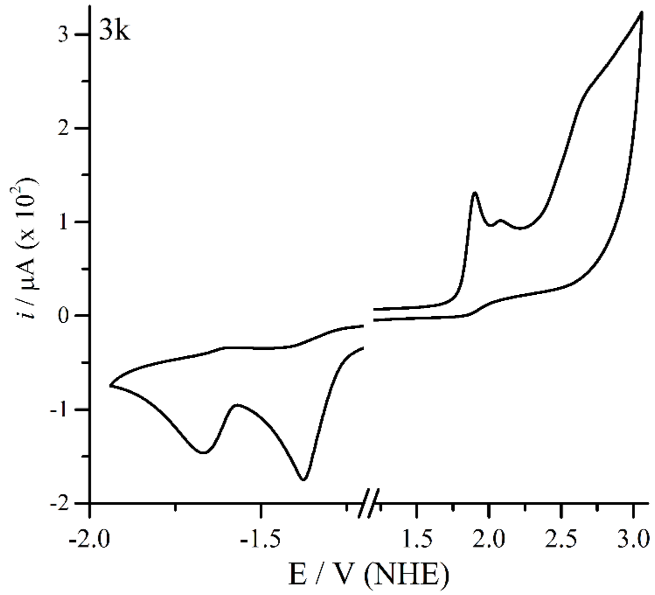


**Figure S6.** Cyclic voltammetry of **3k** in acetonitrile at 100 mV $s^{-1}$.

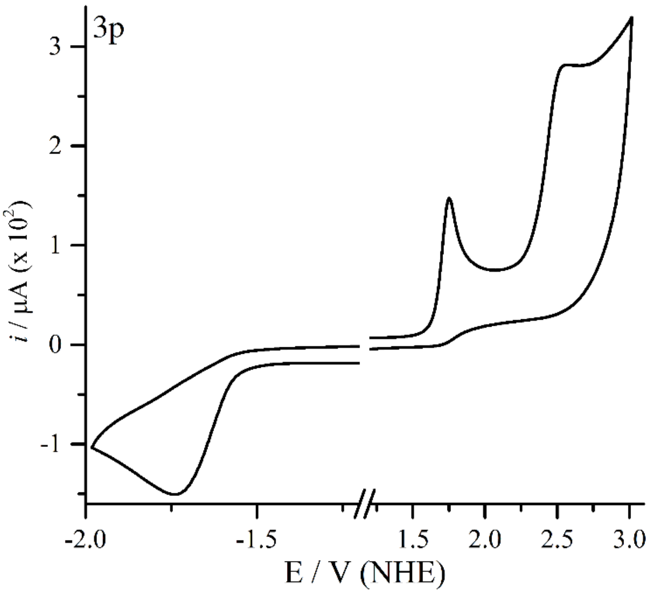


**Figure S7.** Cyclic voltammetry of **3p** in acetonitrile at 100 mV $s^{-1}$.

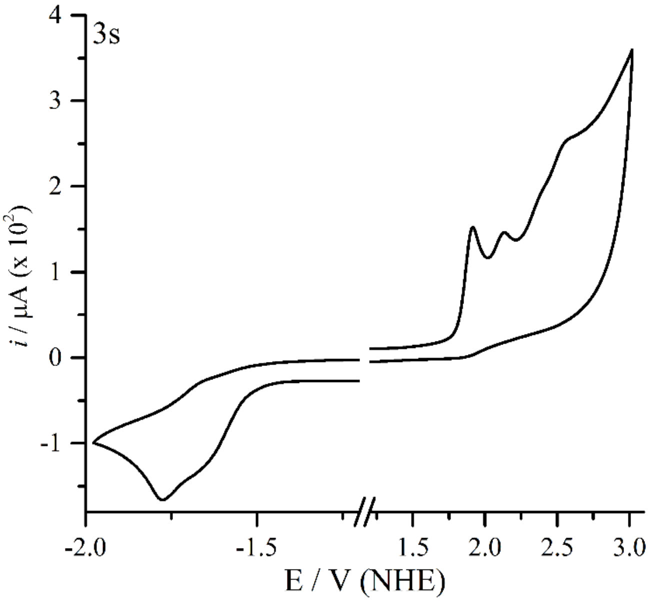


**Figure S8.** Cyclic voltammetry of **3s** in acetonitrile at 100 mV $s^{-1}$.

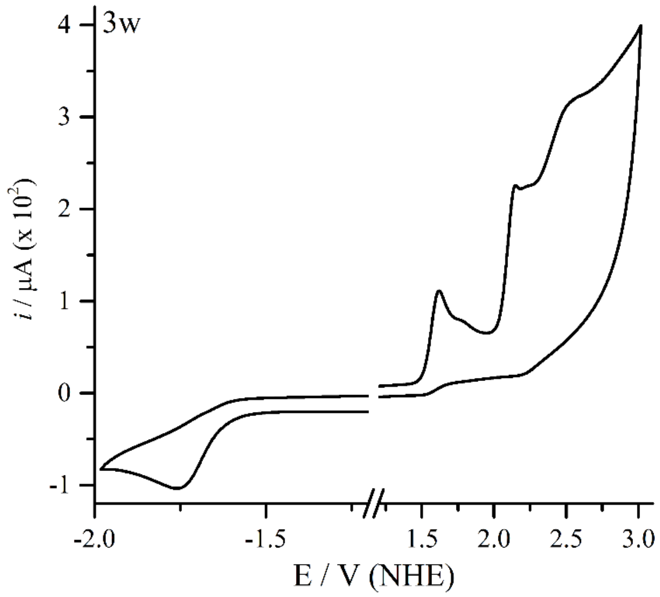


**Figure S9.** Cyclic voltammetry of **3w** in acetonitrile at 100 mV s$^{-1}$.

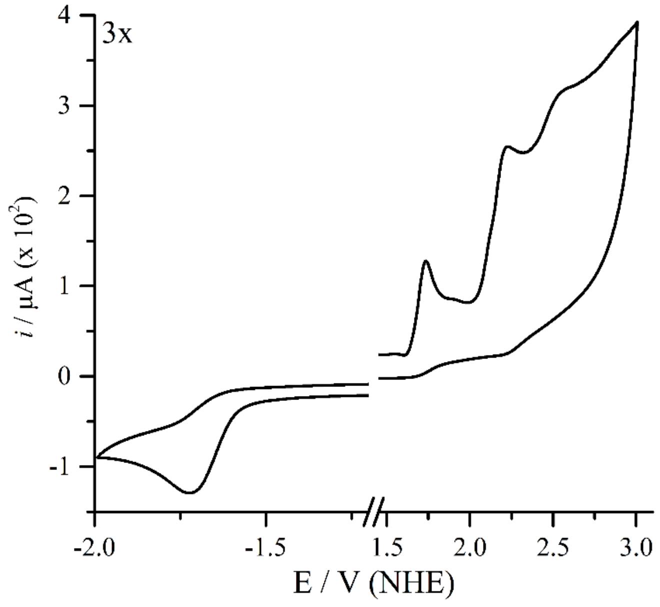


**Figure S10.** Cyclic voltammetry of **3x** in acetonitrile at 100 mV s$^{-1}$.

## NMR Spectra

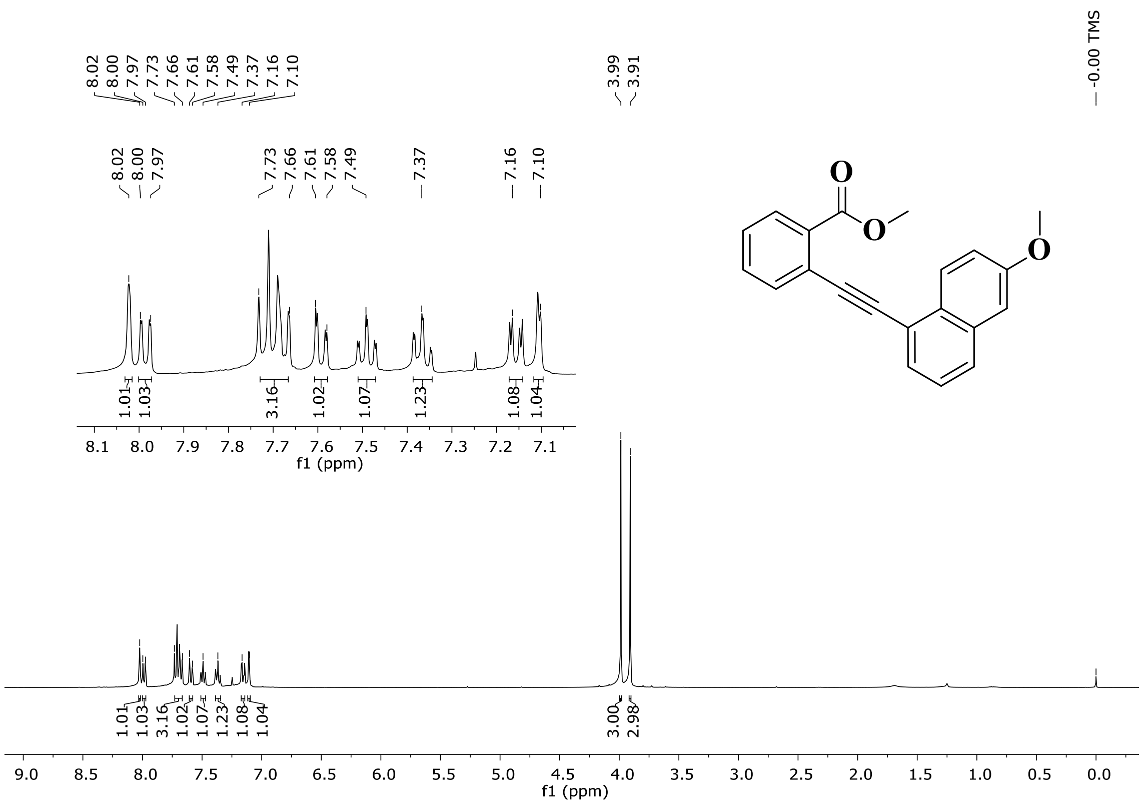

**Figure S11.** $^{1}H$ NMR spectrum (400 MHz) of compound **1m** obtained in $CDCl_3$.

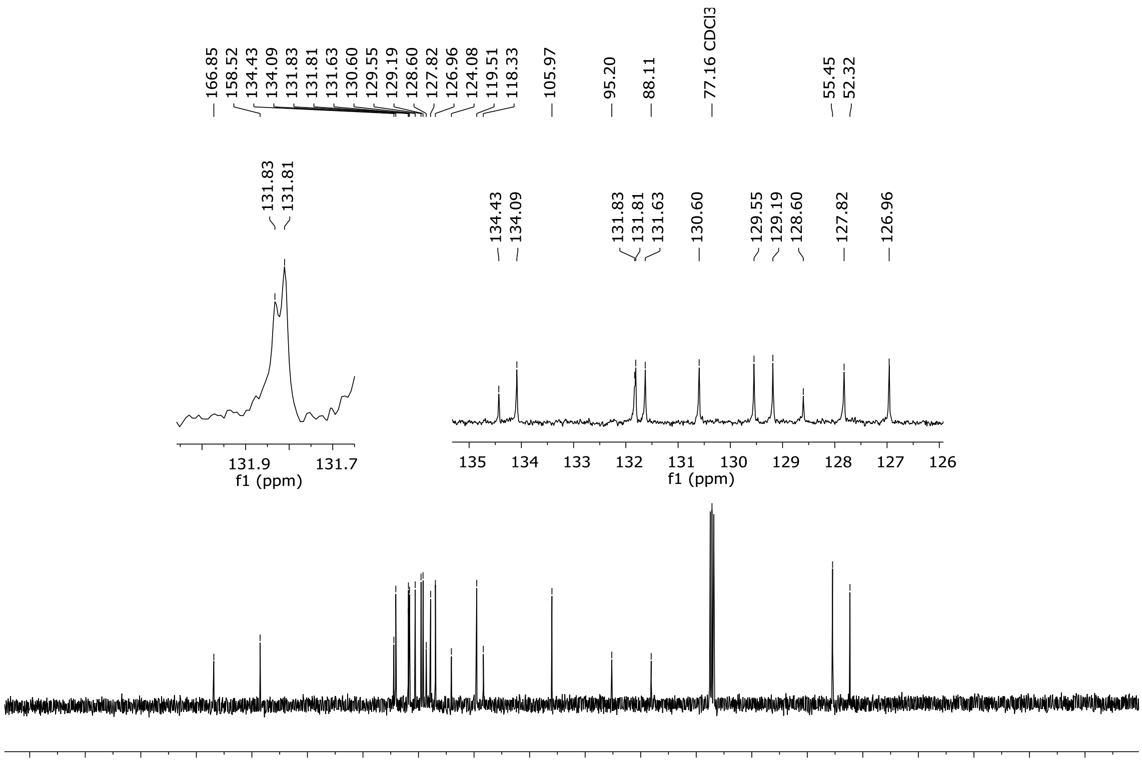

**Figure S12.** $^{13}C$ NMR spectrum (101 MHz) of compound **1m** obtained in $CDCl_3$.

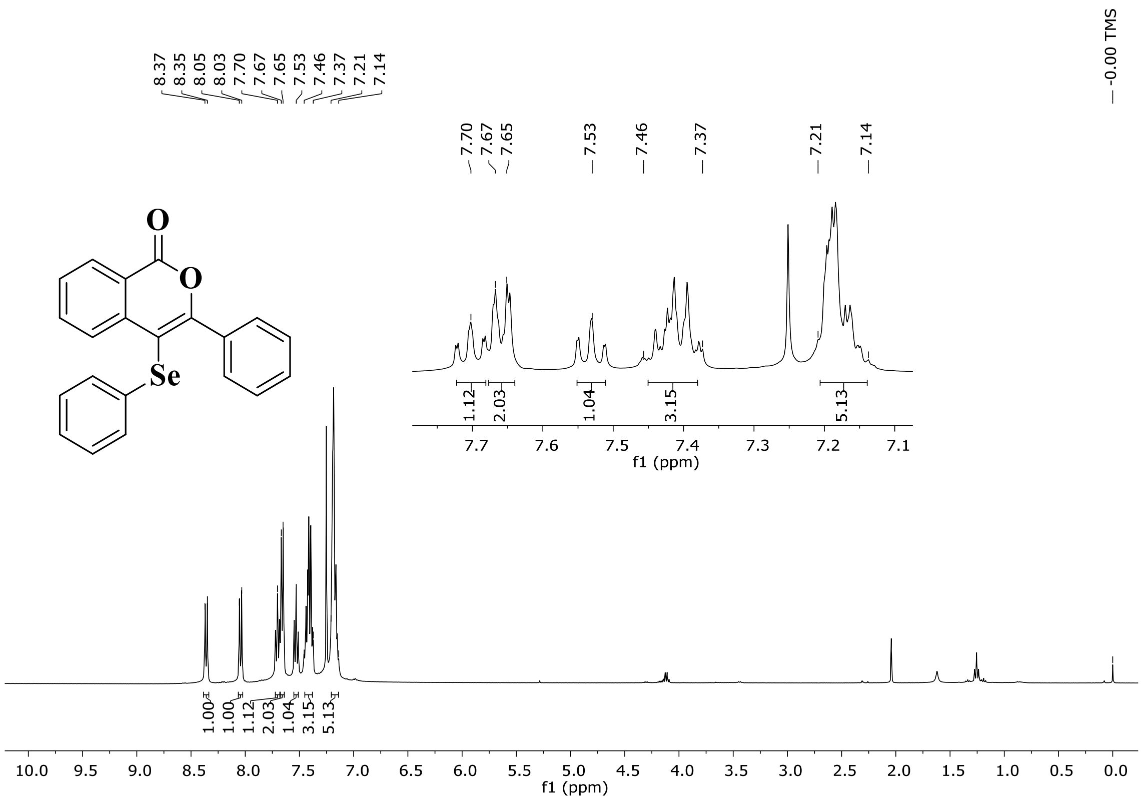

**Figure S13.** $^{1}H$ NMR spectrum (400 MHz) of compound **3a** obtained in $CDCl_3$.

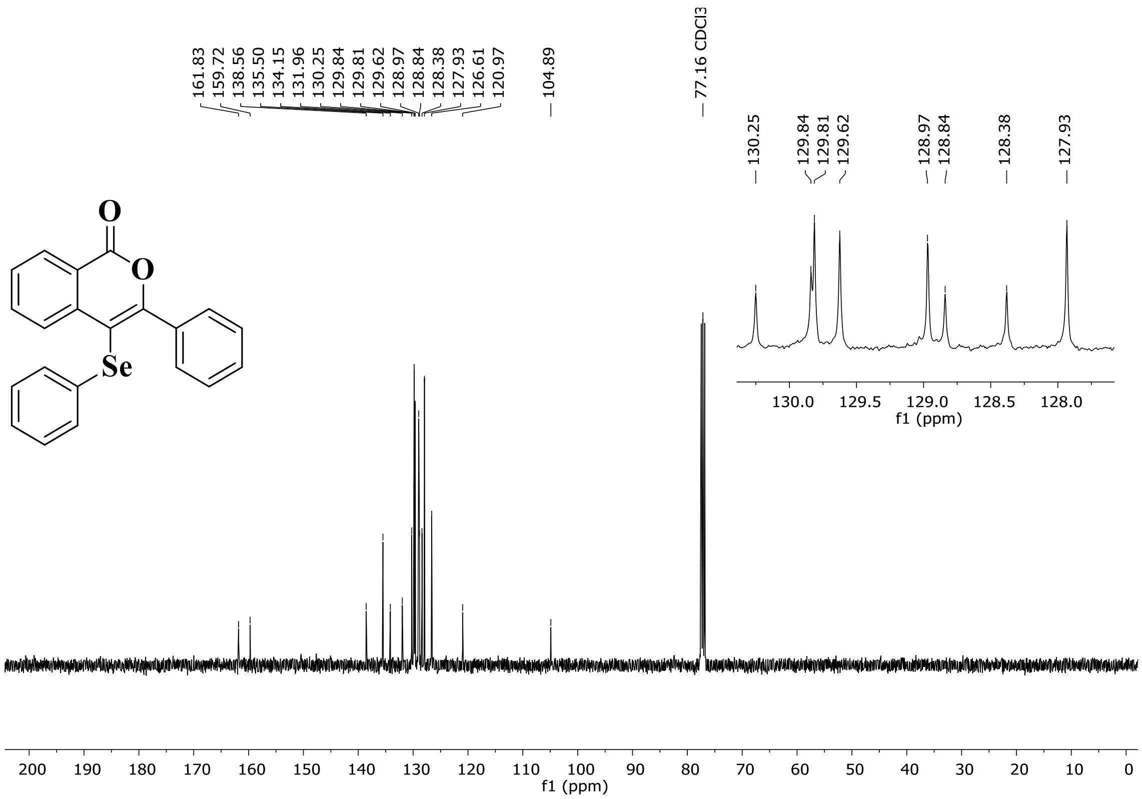

**Figure S14.** $^{13}C$ NMR spectrum (101 MHz) of compound **3a** obtained in $CDCl_3$.

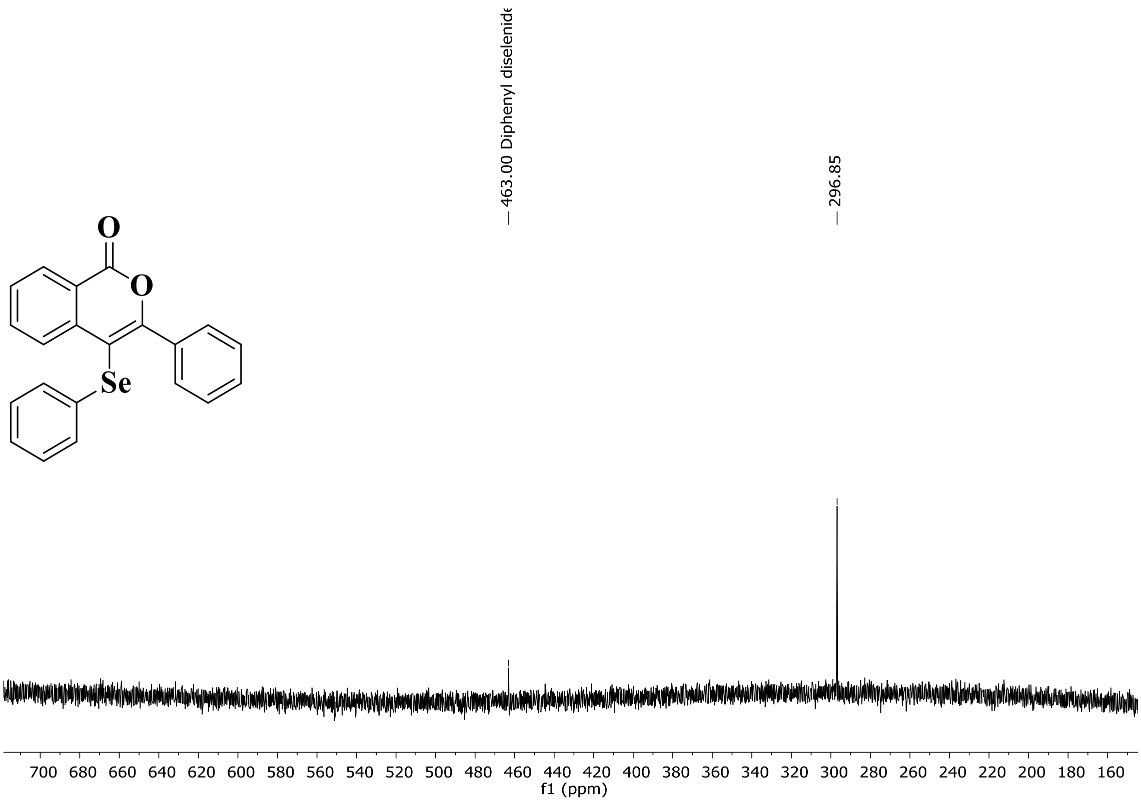


**Figure S15.** $^{77}$Se NMR spectrum (76 MHz) of compound **3a** obtained in $CDCl_3$.

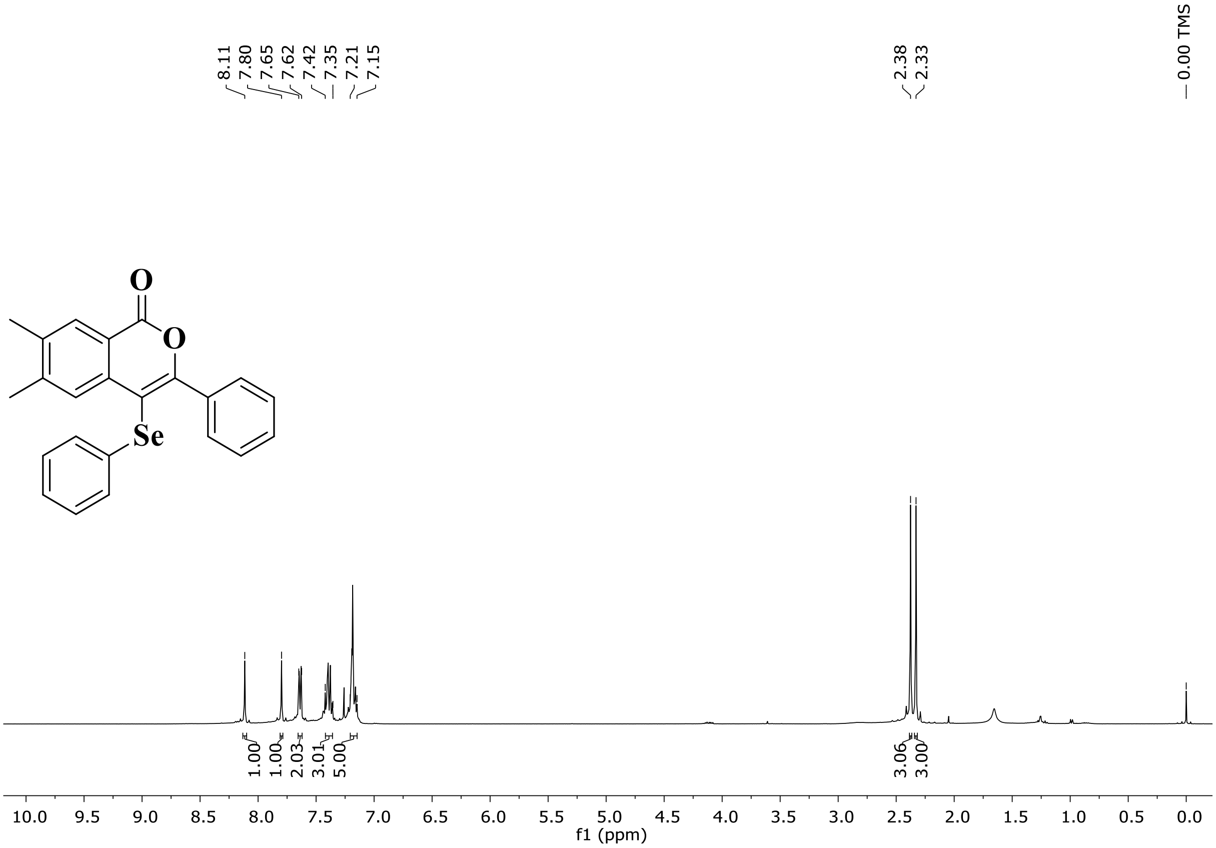


**Figure S16.** $^{1}$H NMR spectrum (400 MHz) of compound **3b** obtained in $CDCl_3$.

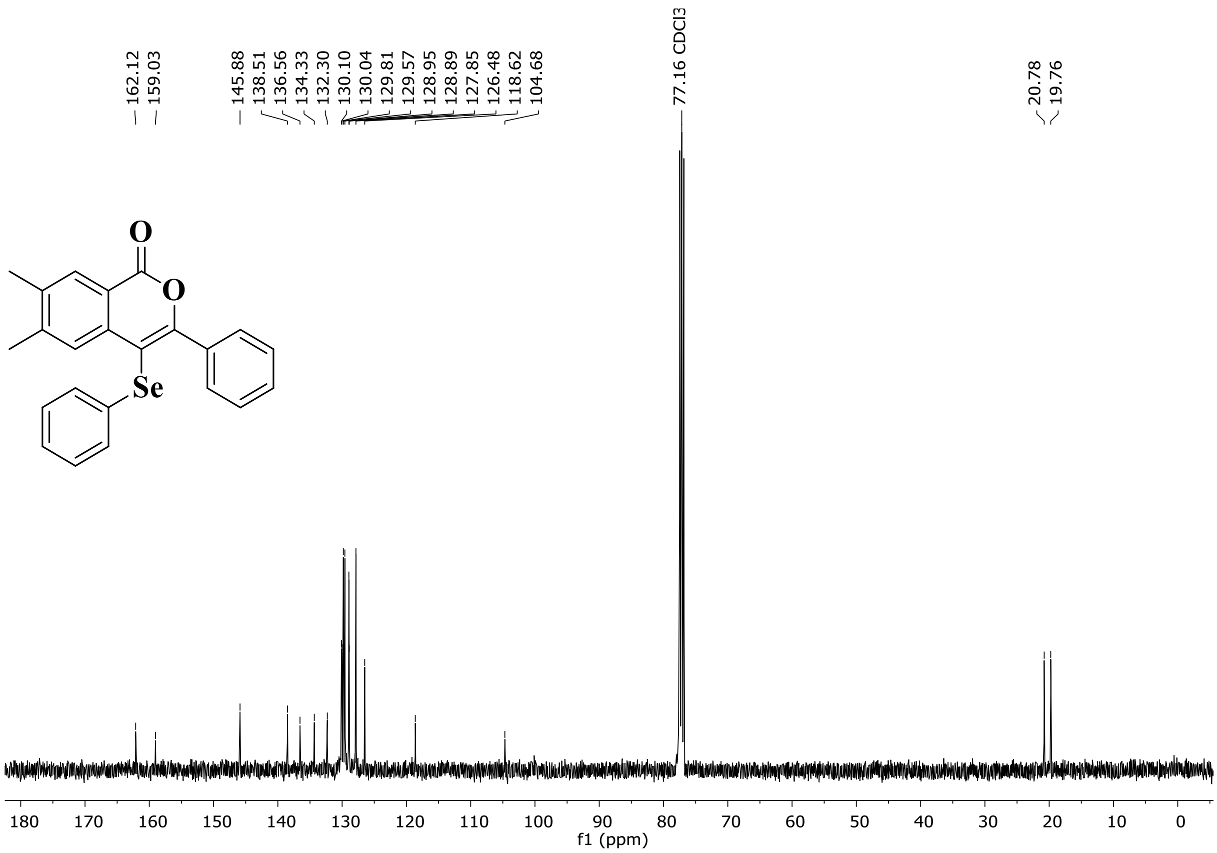


**Figure S17.** $^{13}C$ NMR spectrum (101 MHz) of compound **3b** obtained in $CDCl_3$.

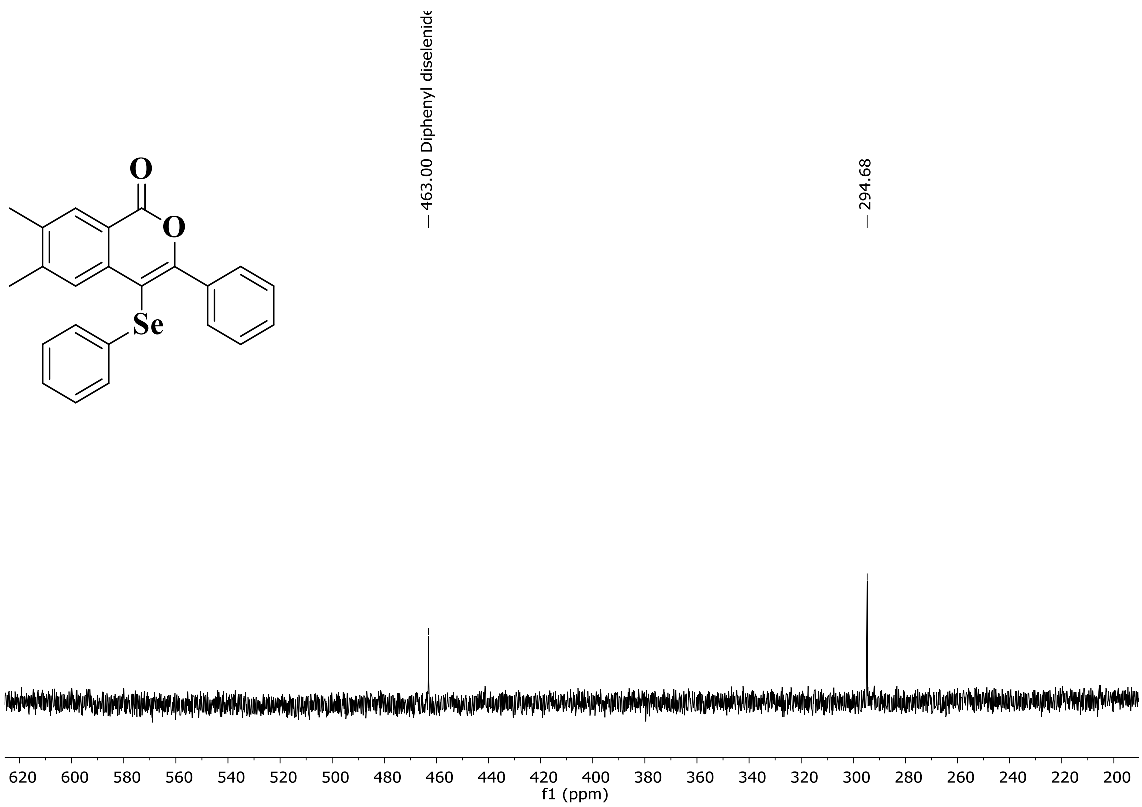


**Figure S18.** $^{77}Se$ NMR spectrum (76 MHz) of compound **3b** obtained in $CDCl_3$.

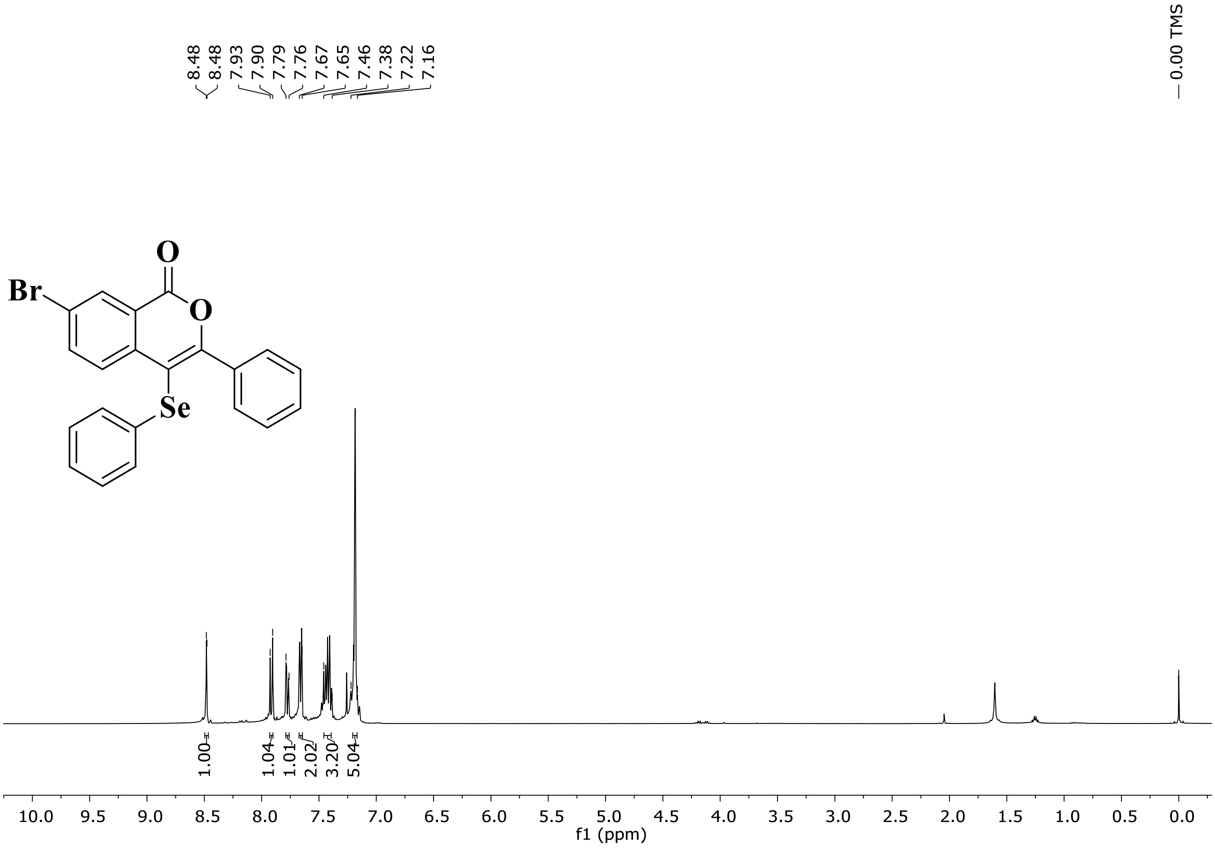


**Figure S19.** $^{1}H$ NMR spectrum (400 MHz) of compound **3c** obtained in $CDCl_3$.

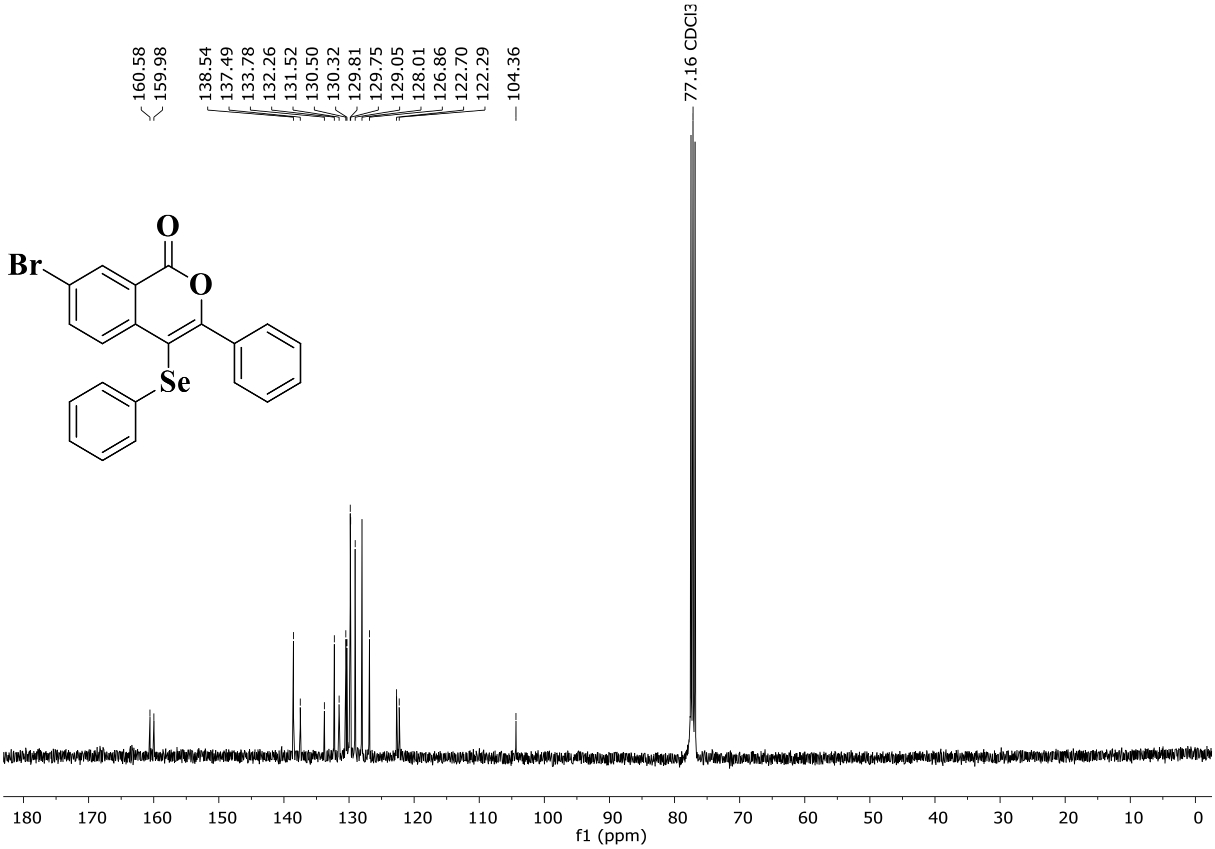


**Figure S20.** $^{13}C$ NMR spectrum (101 MHz) of compound **3c** obtained in $CDCl_3$.

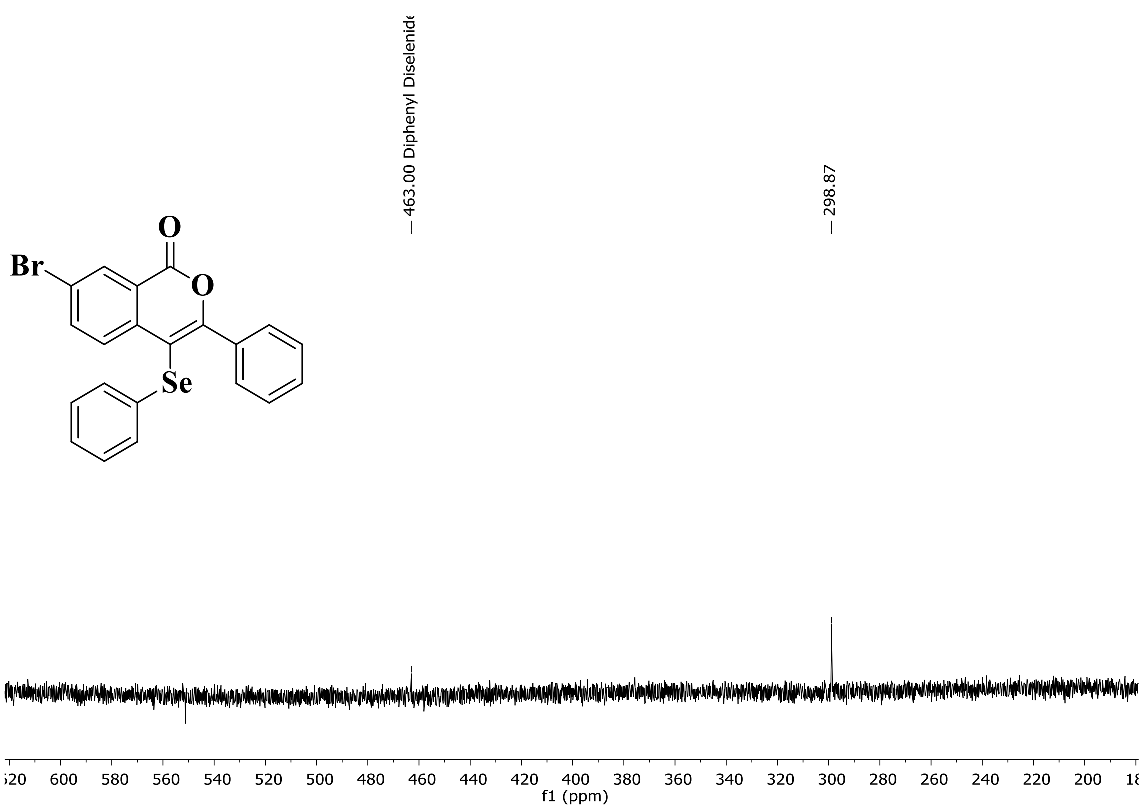


**Figure S21.** $^{77}$Se NMR spectrum (76 MHz) of compound **3c** obtained in $CDCl_3$.

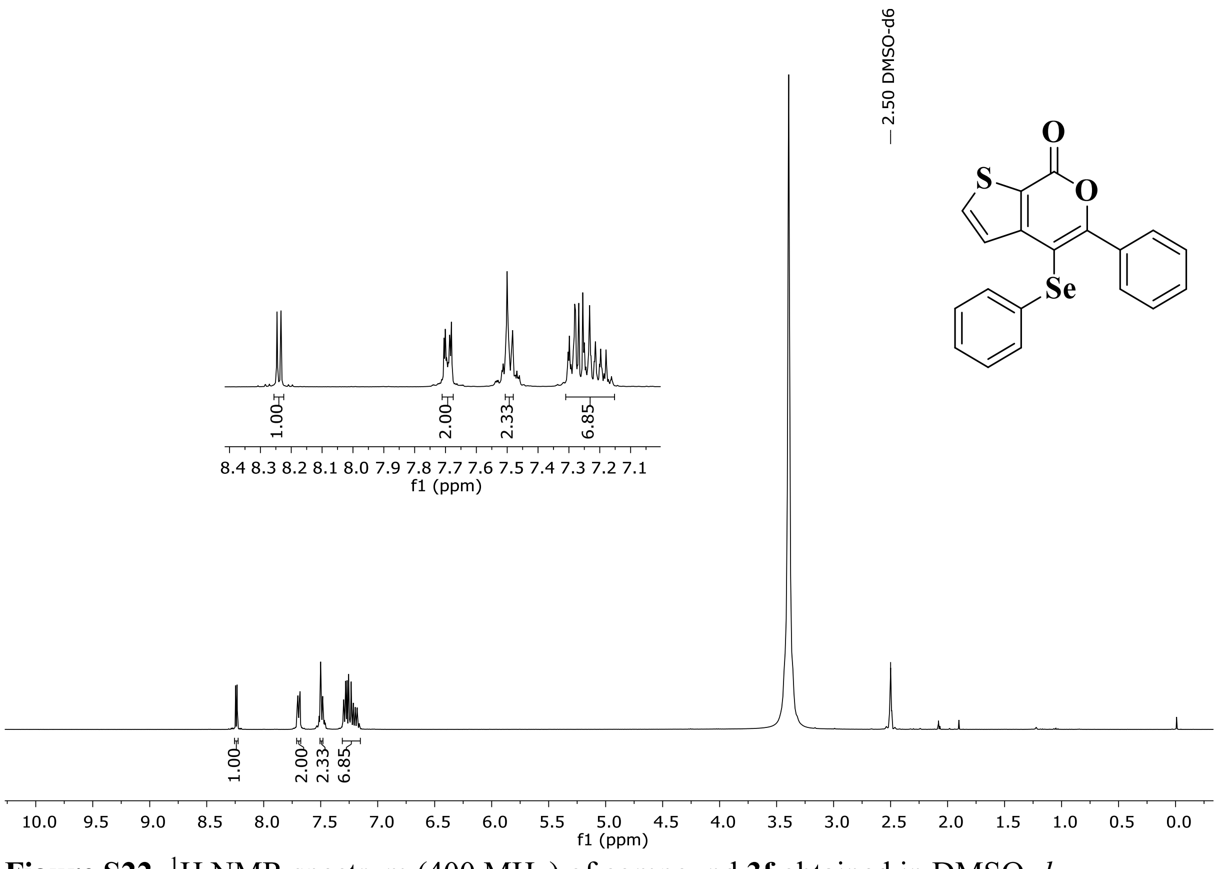


**Figure S22.** $^1$H NMR spectrum (400 MHz) of compound **3f** obtained in DMSO-$d_6$.

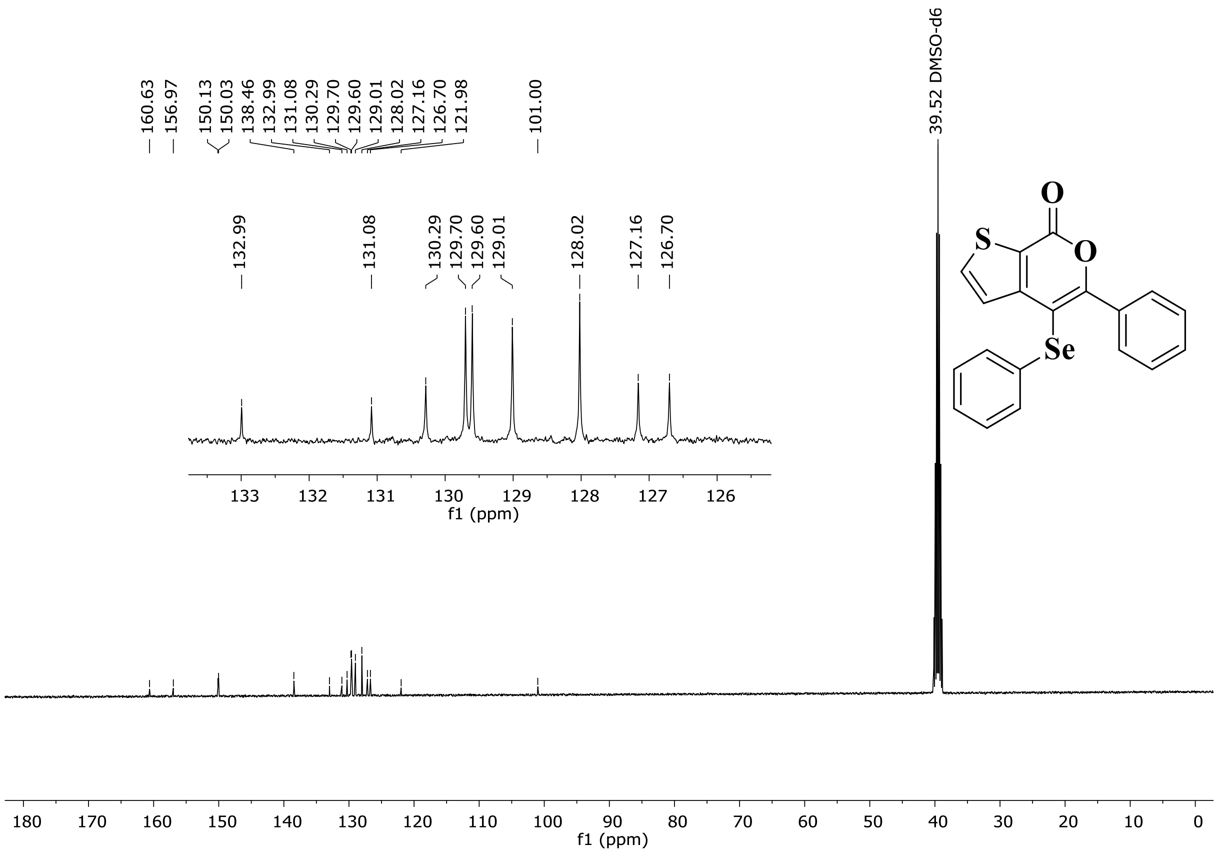


**Figure S23.** $^{13}C$ NMR spectrum (101 MHz) of compound **3f** obtained in DMSO-$d_6$.

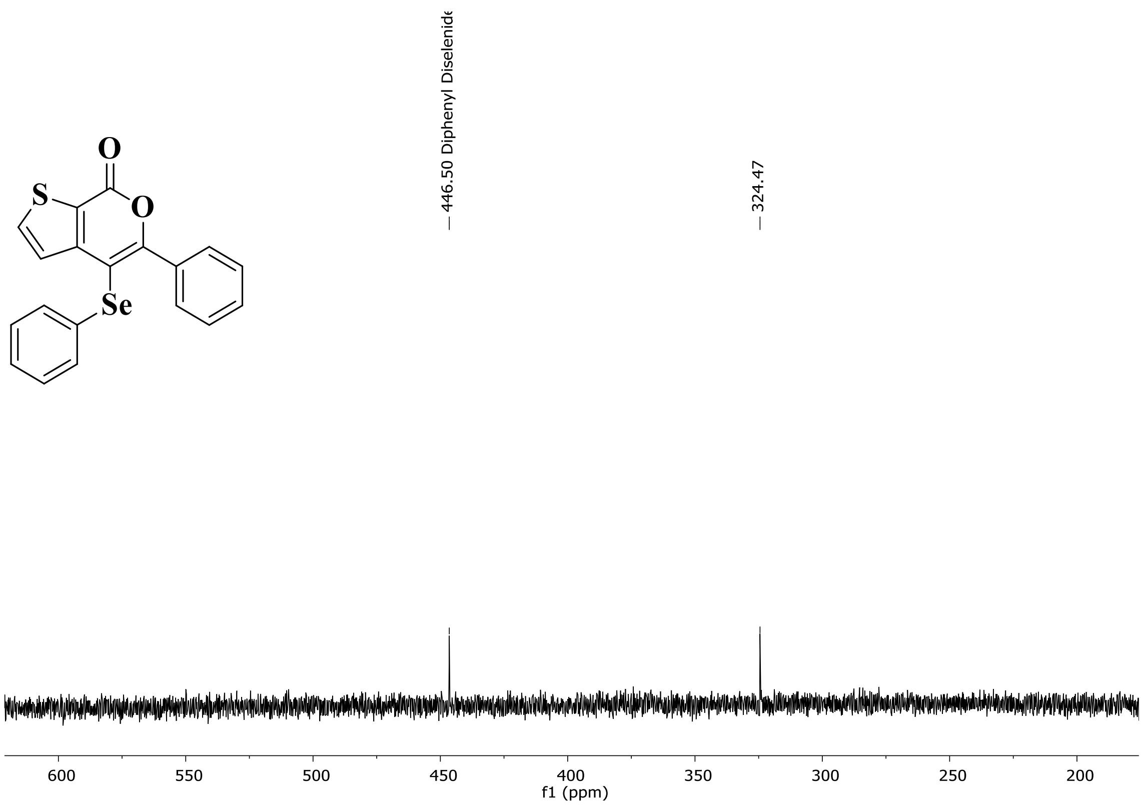


**Figure S24.** $^{77}Se$ NMR spectrum (76 MHz) of compound **3f** obtained in $CDCl_3$.

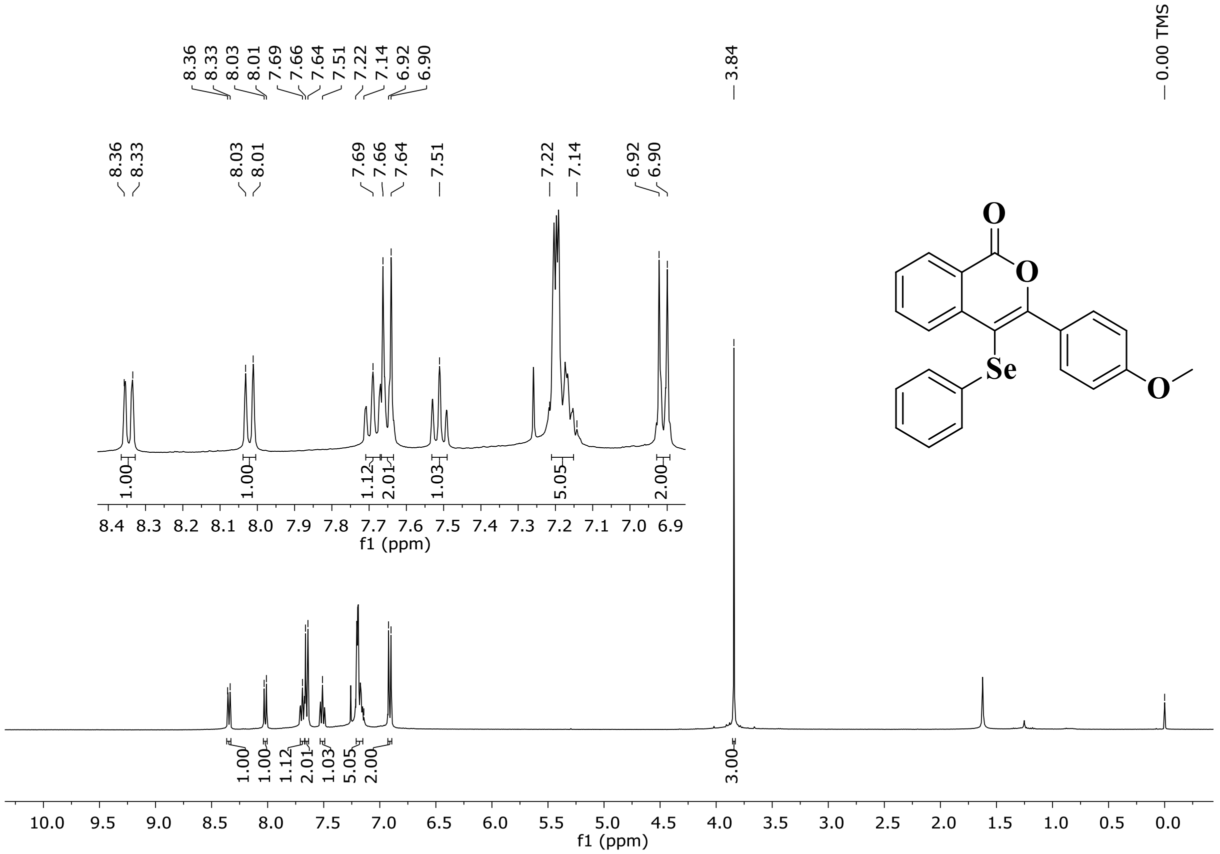

**Figure S25.** $^{1}H$ NMR spectrum (400 MHz) of compound **3g** obtained in $CDCl_3$.

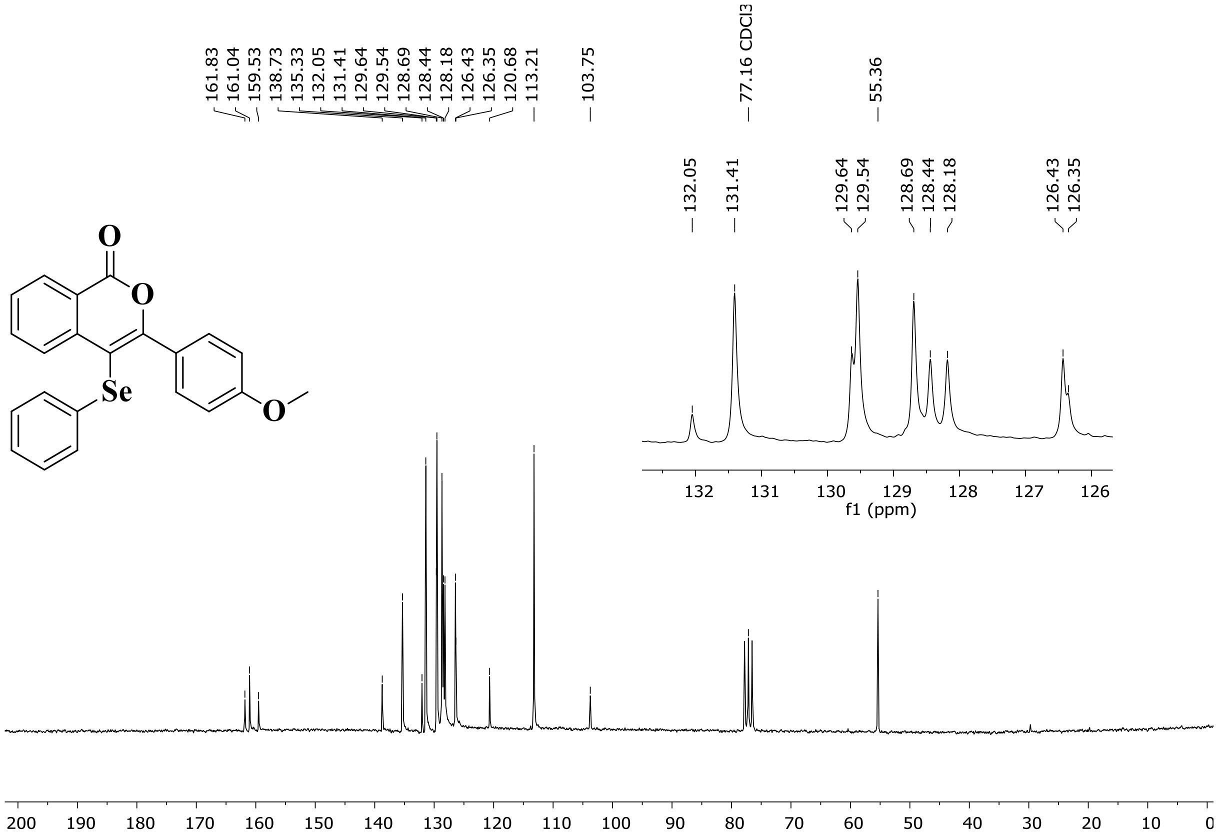

**Figure S26.** $^{13}C$ NMR spectrum (101 MHz) of compound **3g** obtained in $CDCl_3$.

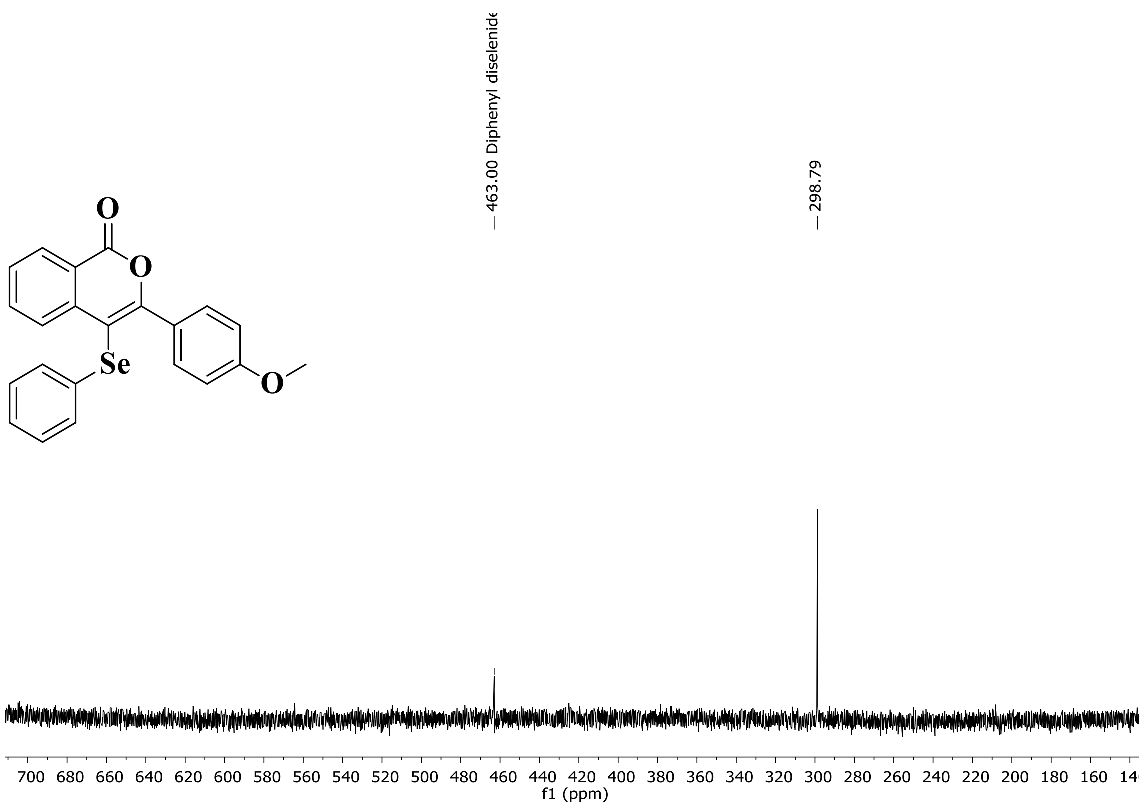

**Figure S27.** $^{77}$Se NMR spectrum (76 MHz) of compound **3g** obtained in $CDCl_3$.

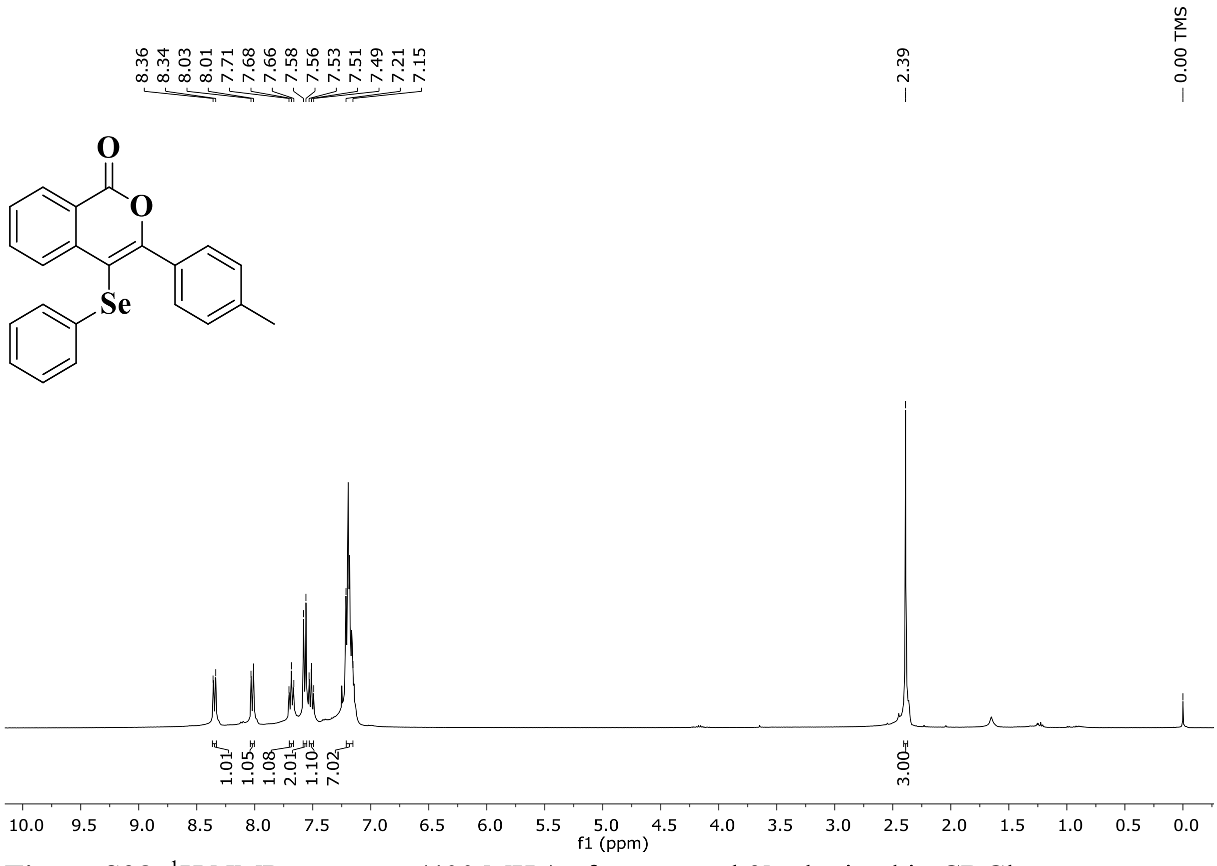

**Figure S28.** $^{1}$H NMR spectrum (400 MHz) of compound **3h** obtained in $CDCl_3$.

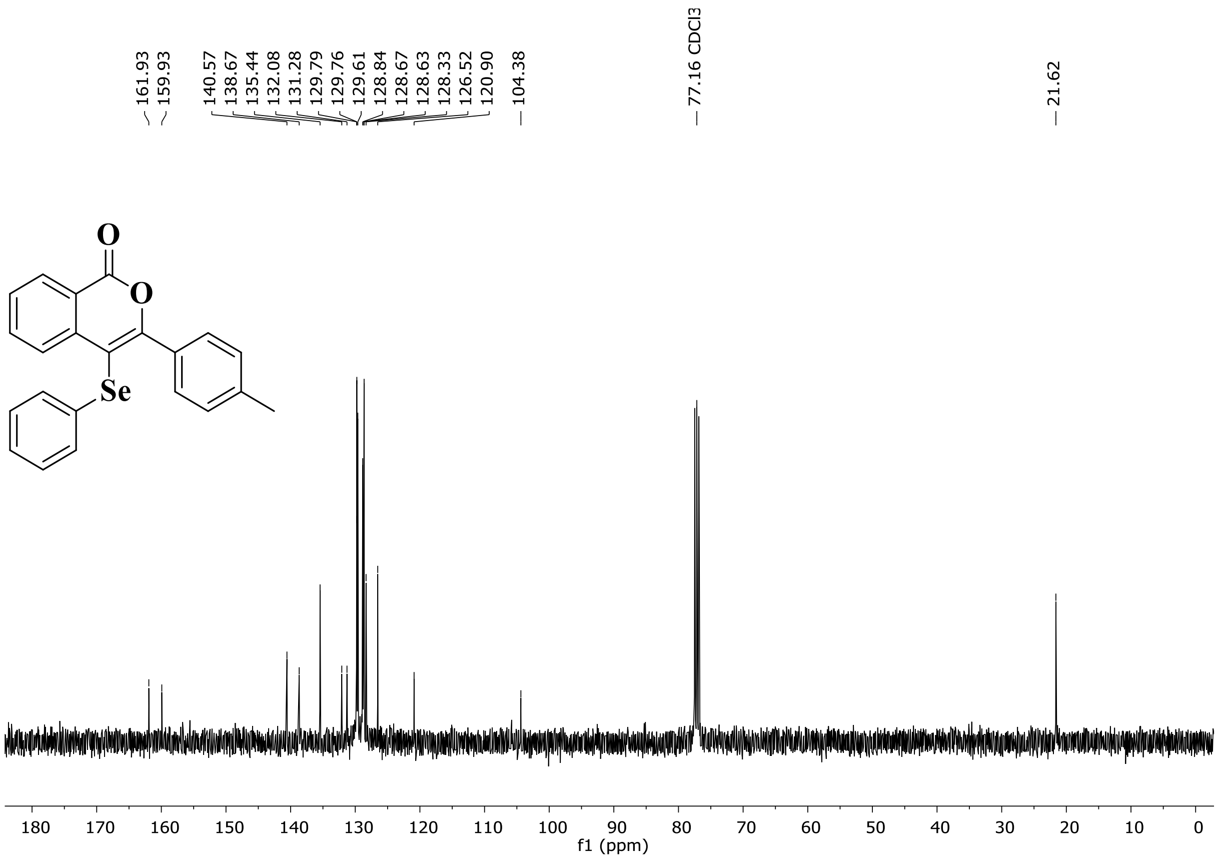


**Figure S29.** $^{13}C$ NMR spectrum (101 MHz) of compound **3h** obtained in $CDCl_3$.

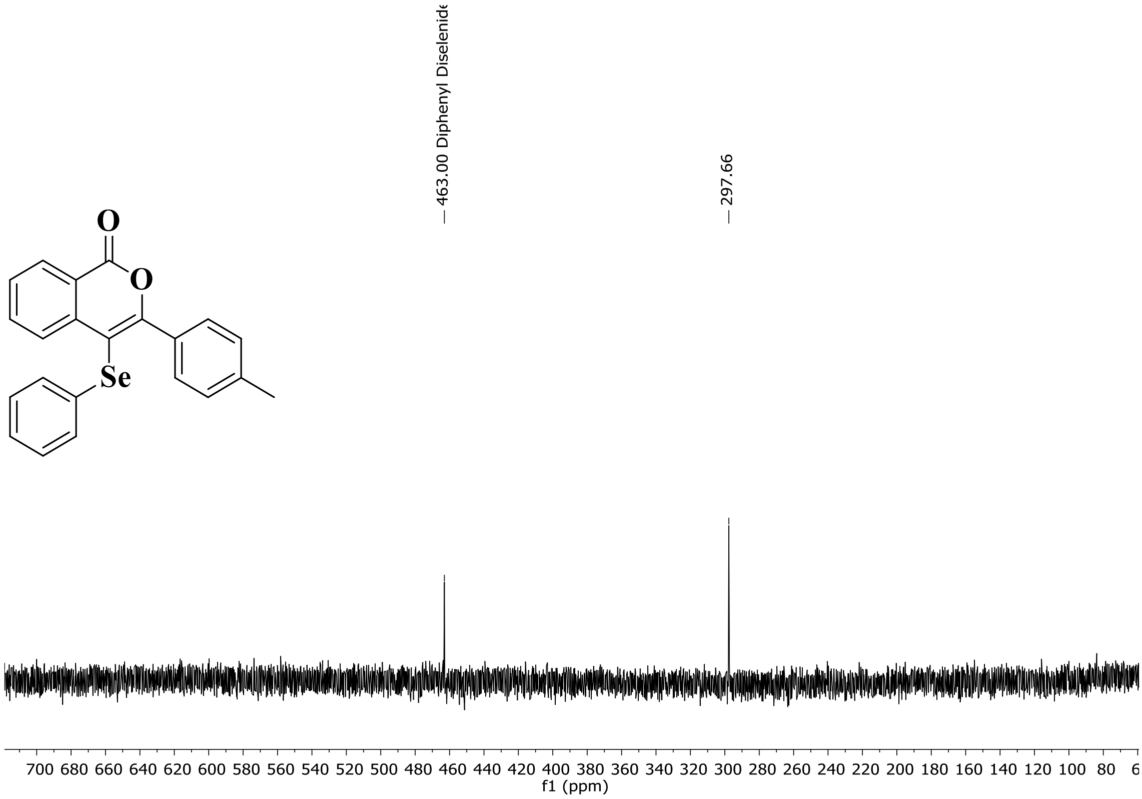


**Figure S30.** $^{77}Se$ NMR spectrum (76 MHz) of compound **3h** obtained in $CDCl_3$.

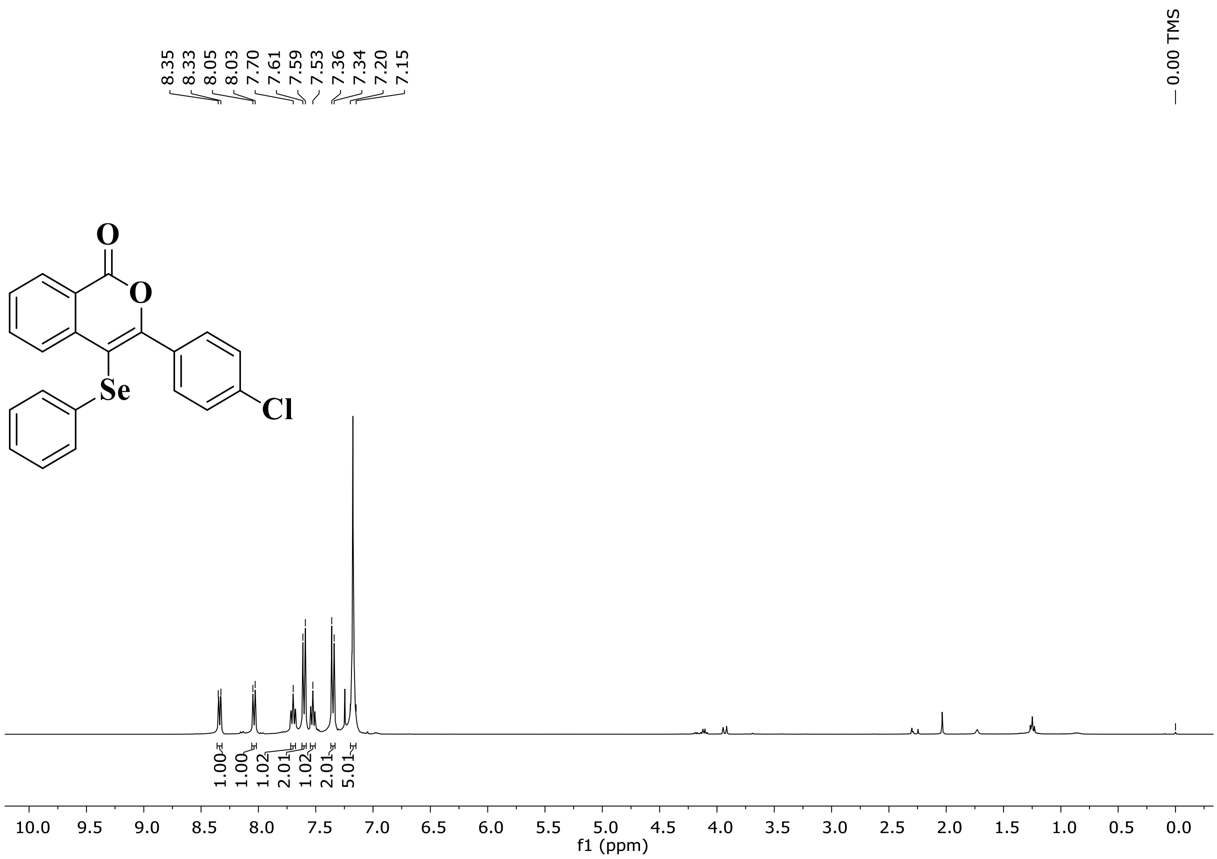

**Figure S31.** $^{1}$H NMR spectrum (400 MHz) of compound **3i** obtained in $CDCl_3$.

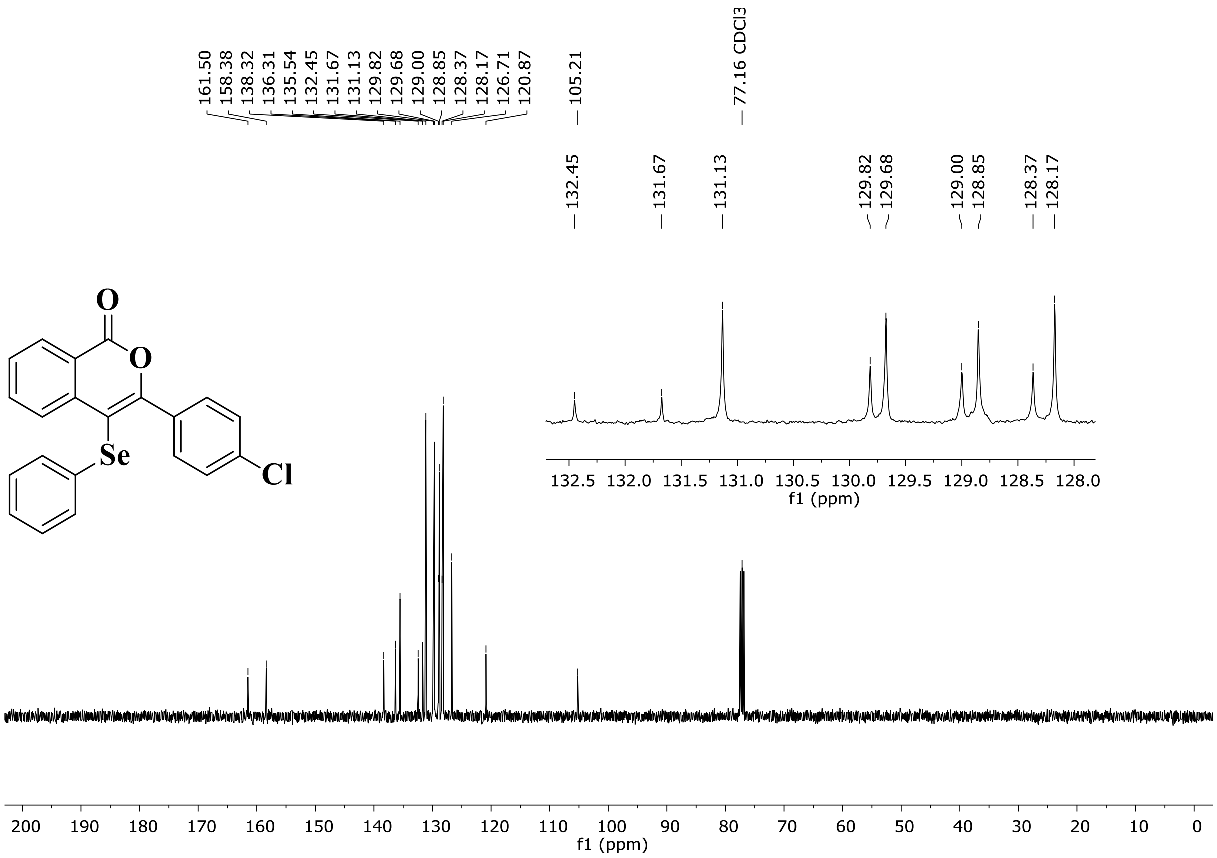

**Figure S32.** $^{13}$C NMR spectrum (101 MHz) of compound **3i** obtained in $CDCl_3$.

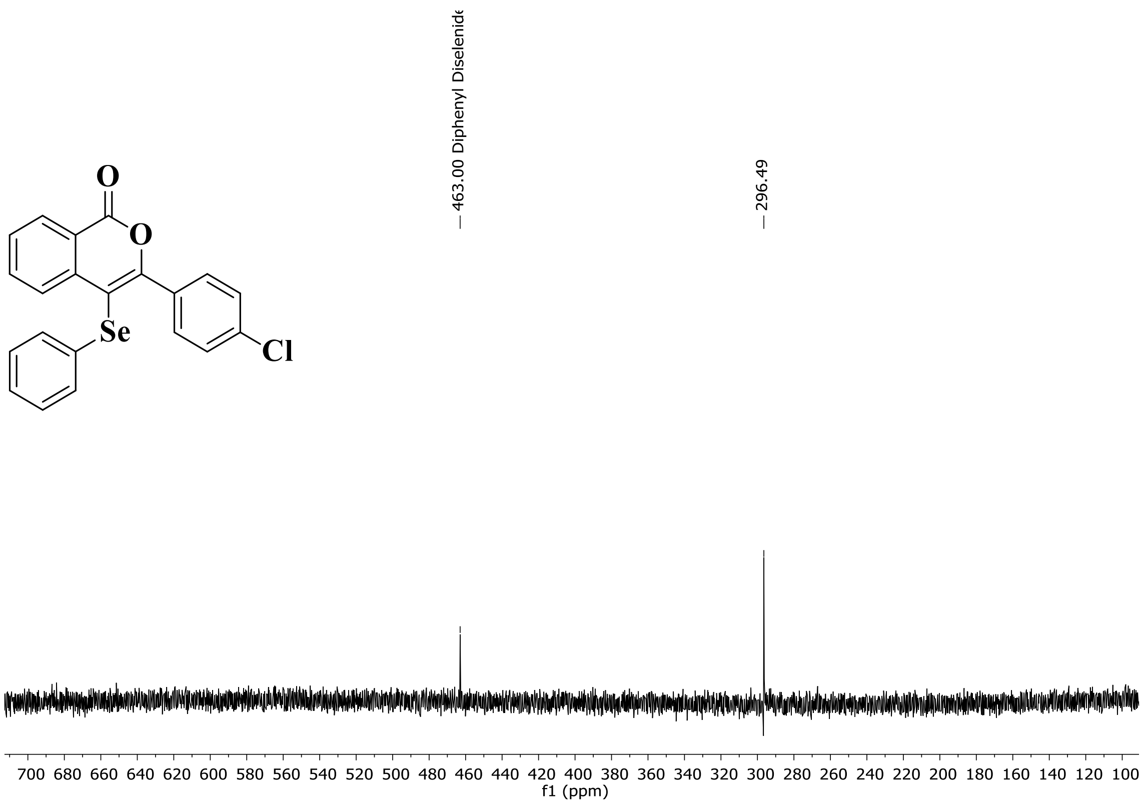


**Figure S33.** $^{77}$Se NMR spectrum (76 MHz) of compound **3i** obtained in $CDCl_3$.

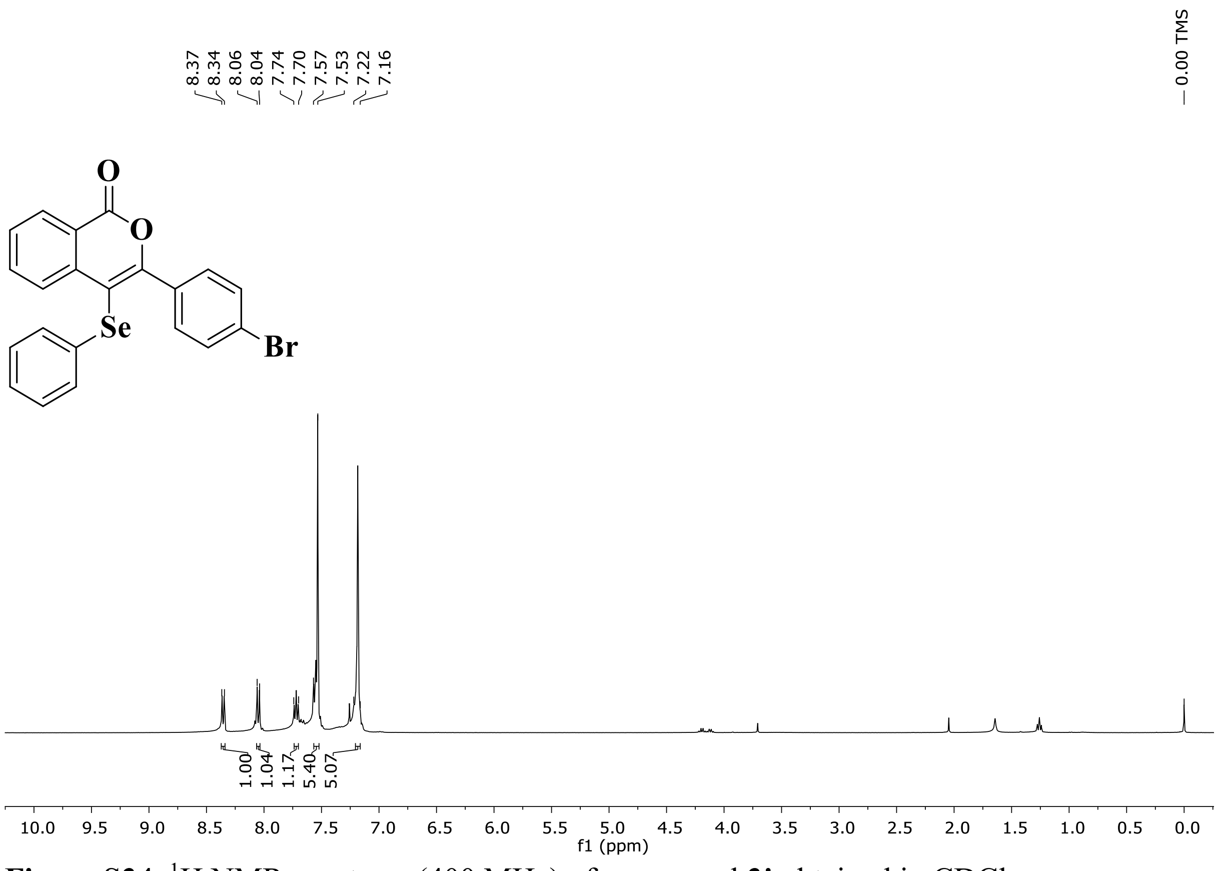


**Figure S34.** $^{1}$H NMR spectrum (400 MHz) of compound **3j** obtained in $CDCl_3$.

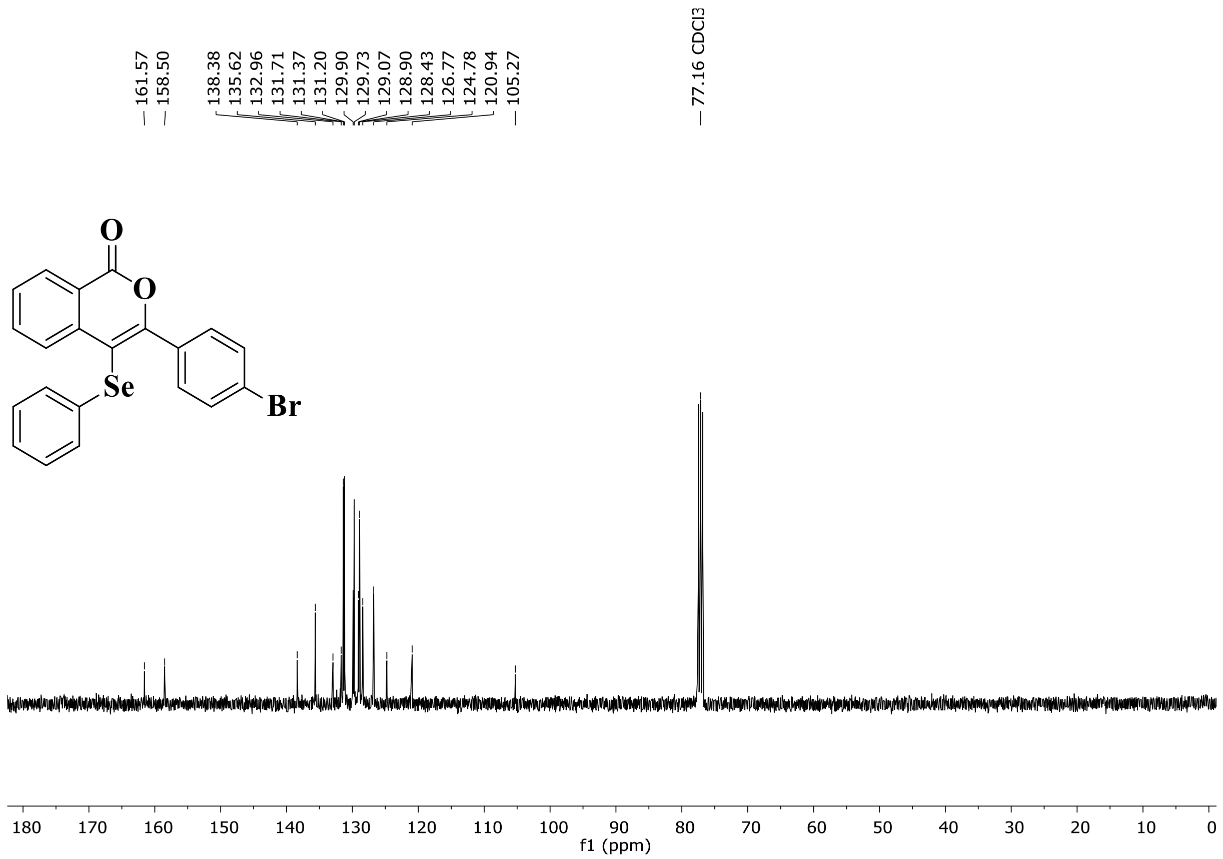


**Figure S35.** $^{13}C$ NMR spectrum (101 MHz) of compound **3j** obtained in $CDCl_3$.

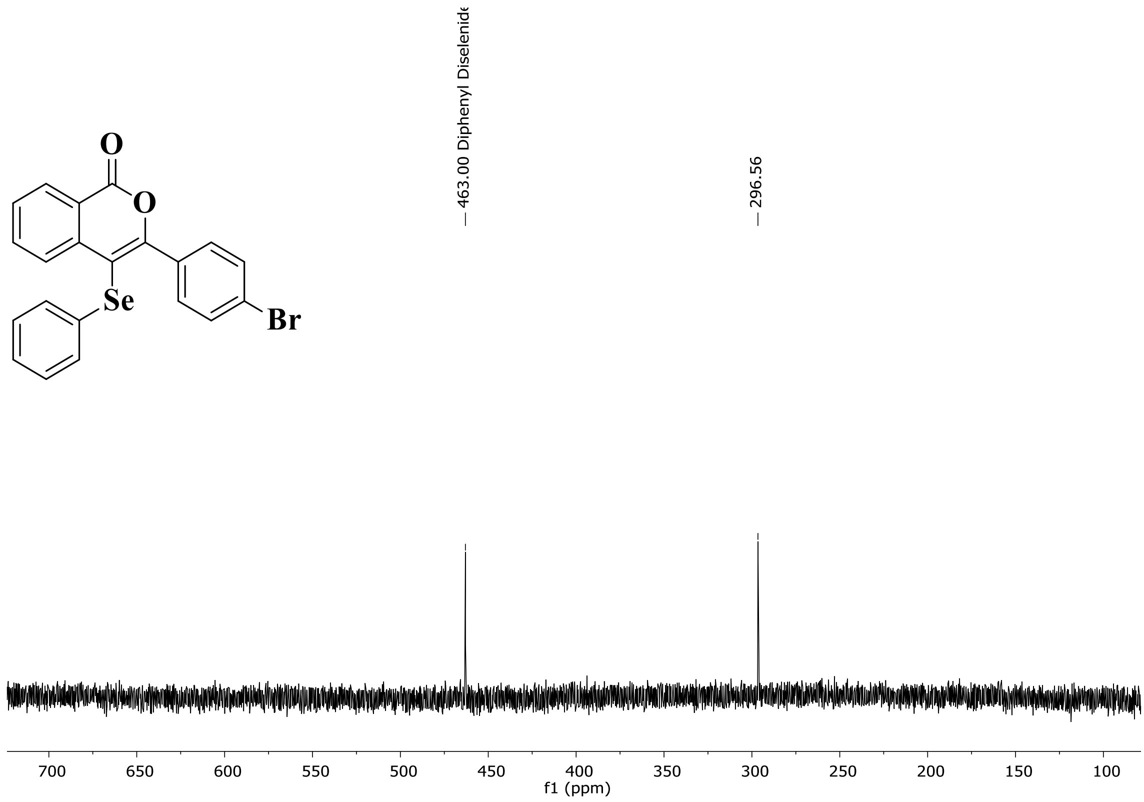


**Figure S36.** $^{77}Se$ NMR spectrum (76 MHz) of compound **3j** obtained in $CDCl_3$.

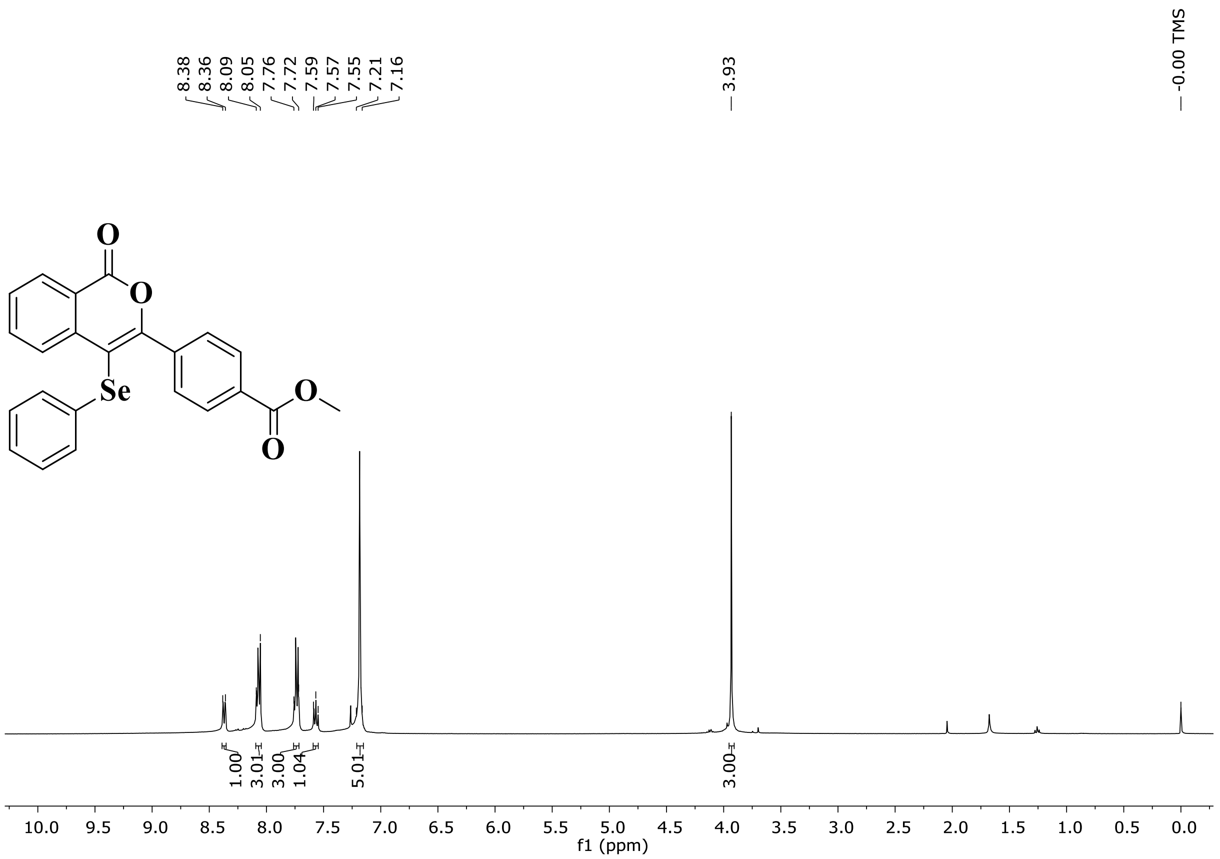

**Figure S37.** $^{1}H$ NMR spectrum (400 MHz) of compound **3k** obtained in $CDCl_3$.

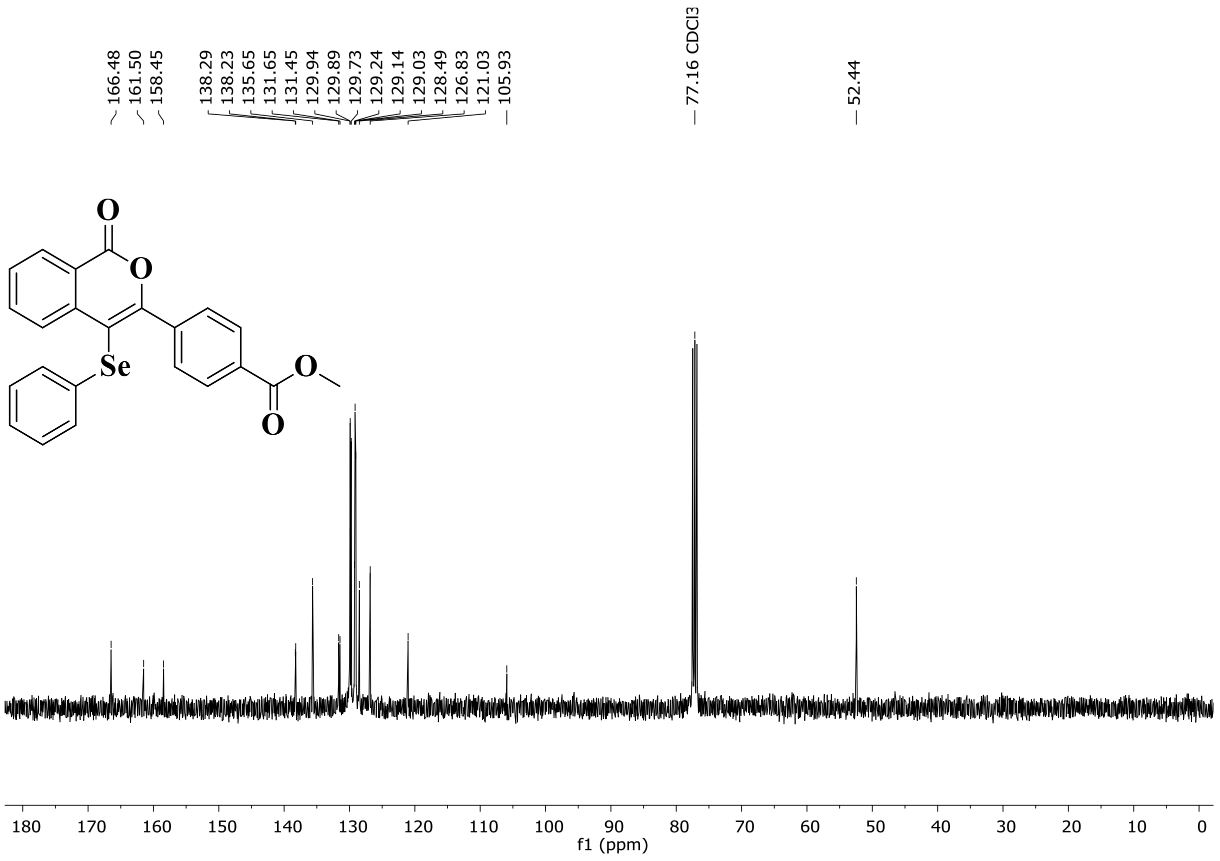

**Figure S38.** $^{13}C$ NMR spectrum (101 MHz) of compound **3k** obtained in $CDCl_3$.

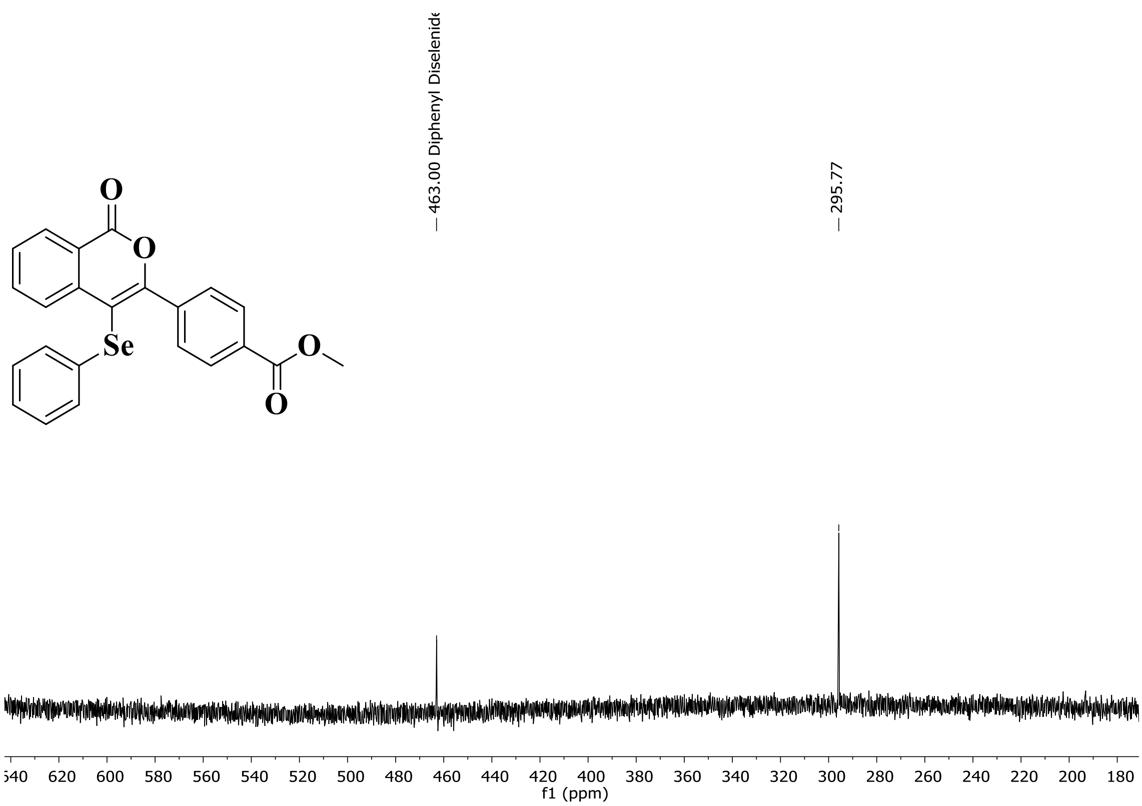


**Figure S39.** $^{77}$Se NMR spectrum (76 MHz) of compound **3k** obtained in $CDCl_3$.

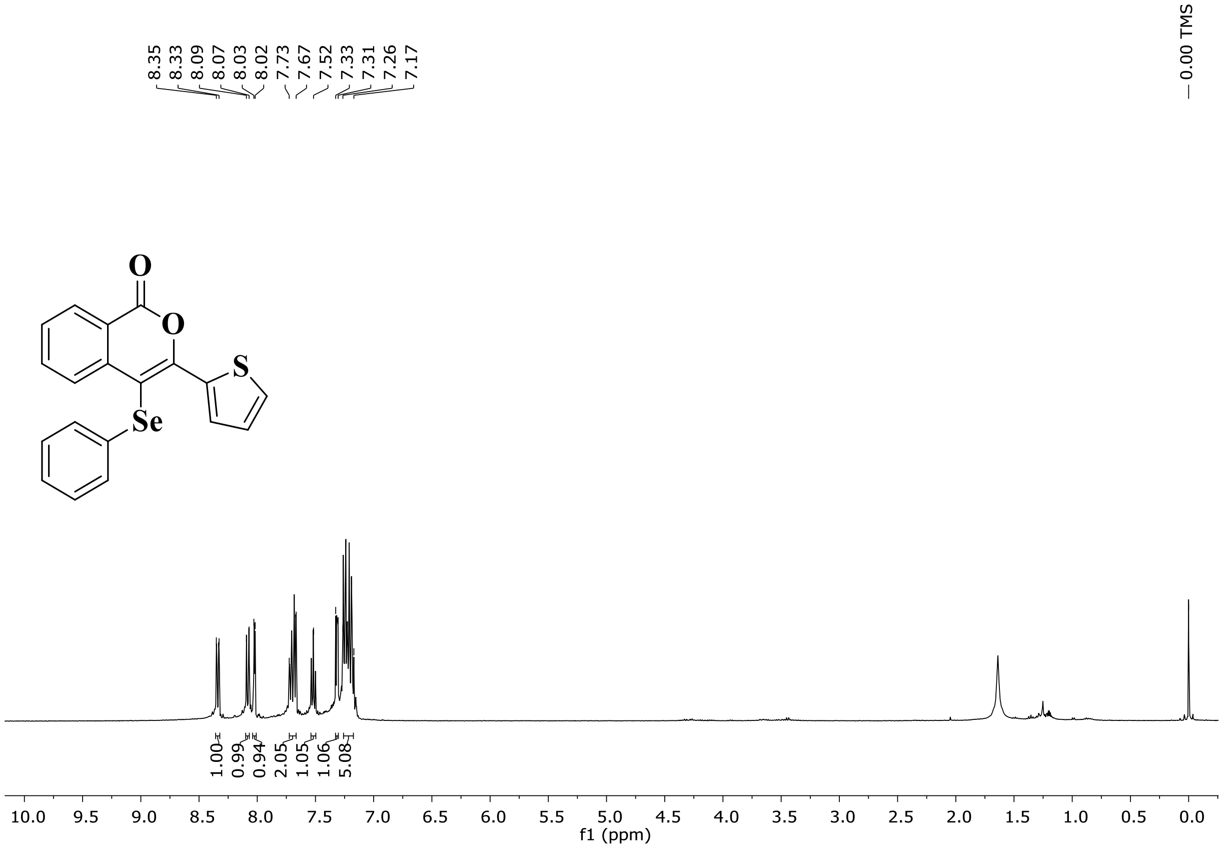


**Figure S40.** $^{1}$H NMR spectrum (400 MHz) of compound **3l** obtained in $CDCl_3$.

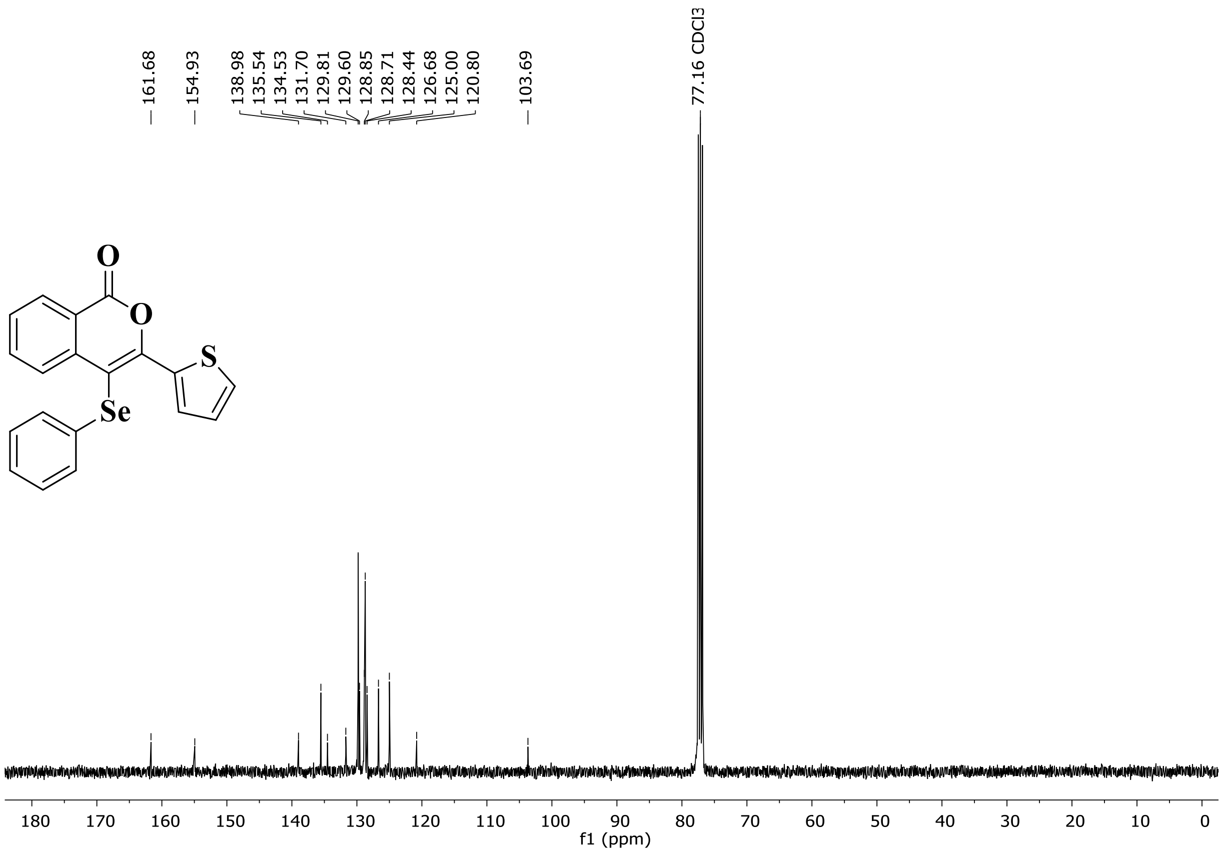


**Figure S41.** $^{13}$C NMR spectrum (101 MHz) of compound **3l** obtained in $CDCl_3$.

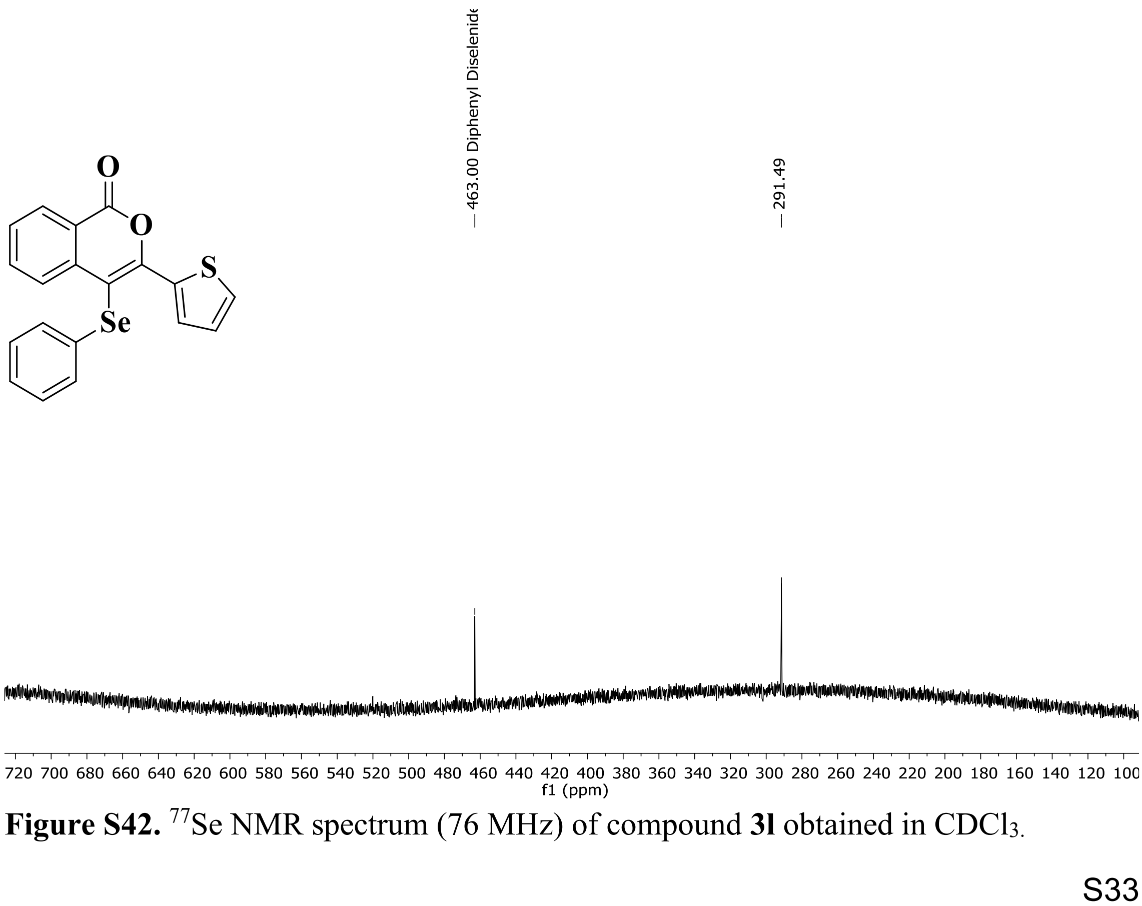


**Figure S42.** $^{77}$Se NMR spectrum (76 MHz) of compound **3l** obtained in $CDCl_3$.

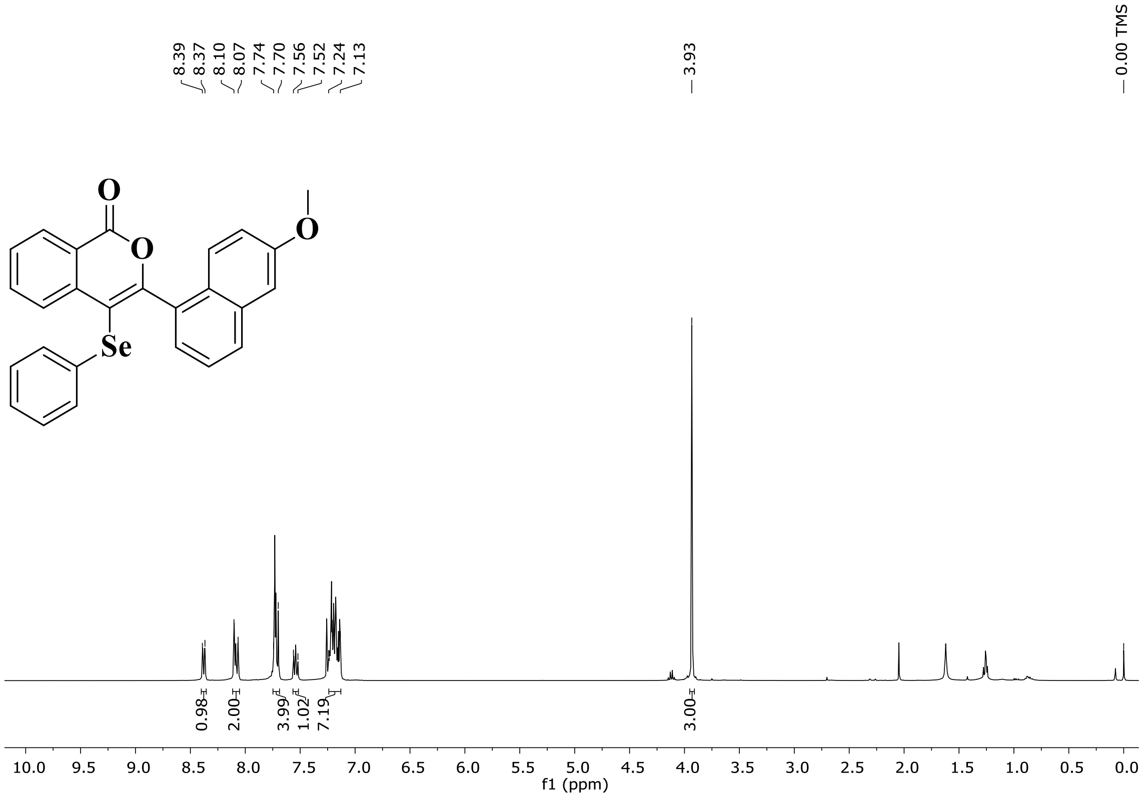


**Figure S43.** $^{1}H$ NMR spectrum (400 MHz) of compound **3m** obtained in $CDCl_3$.

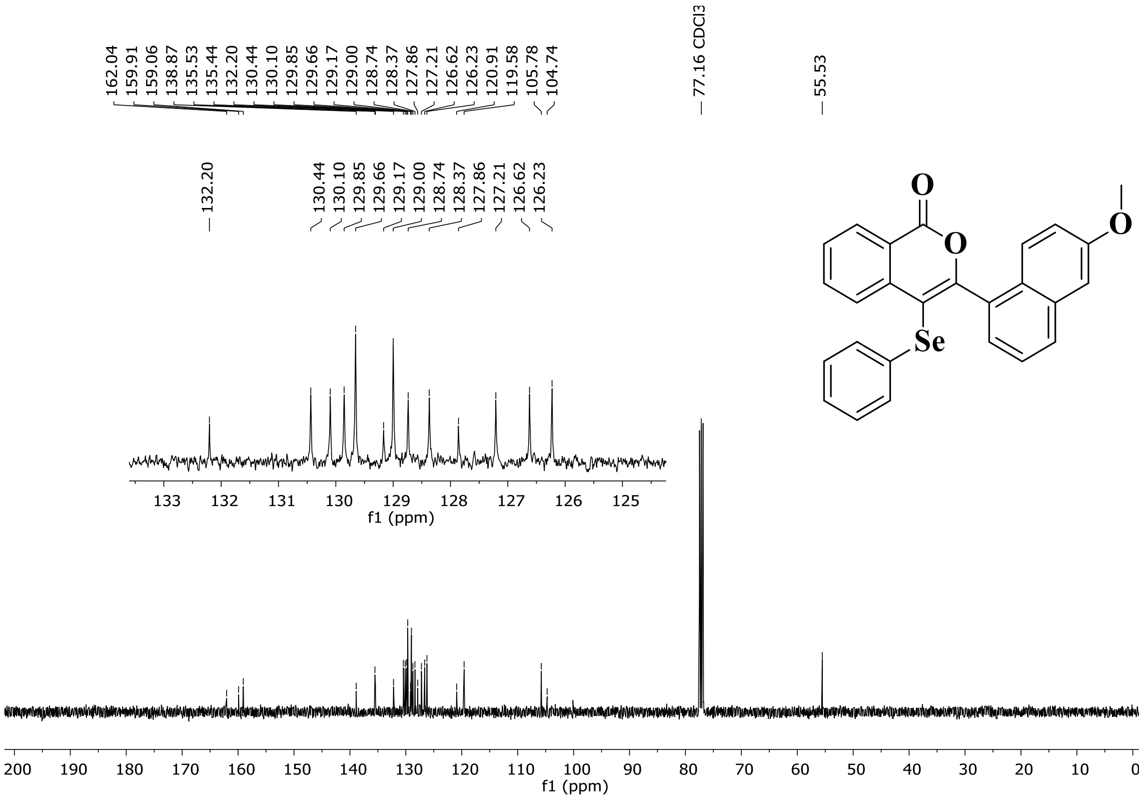


**Figure S44.** $^{13}C$ NMR spectrum (101 MHz) of compound **3m** obtained in $CDCl_3$.

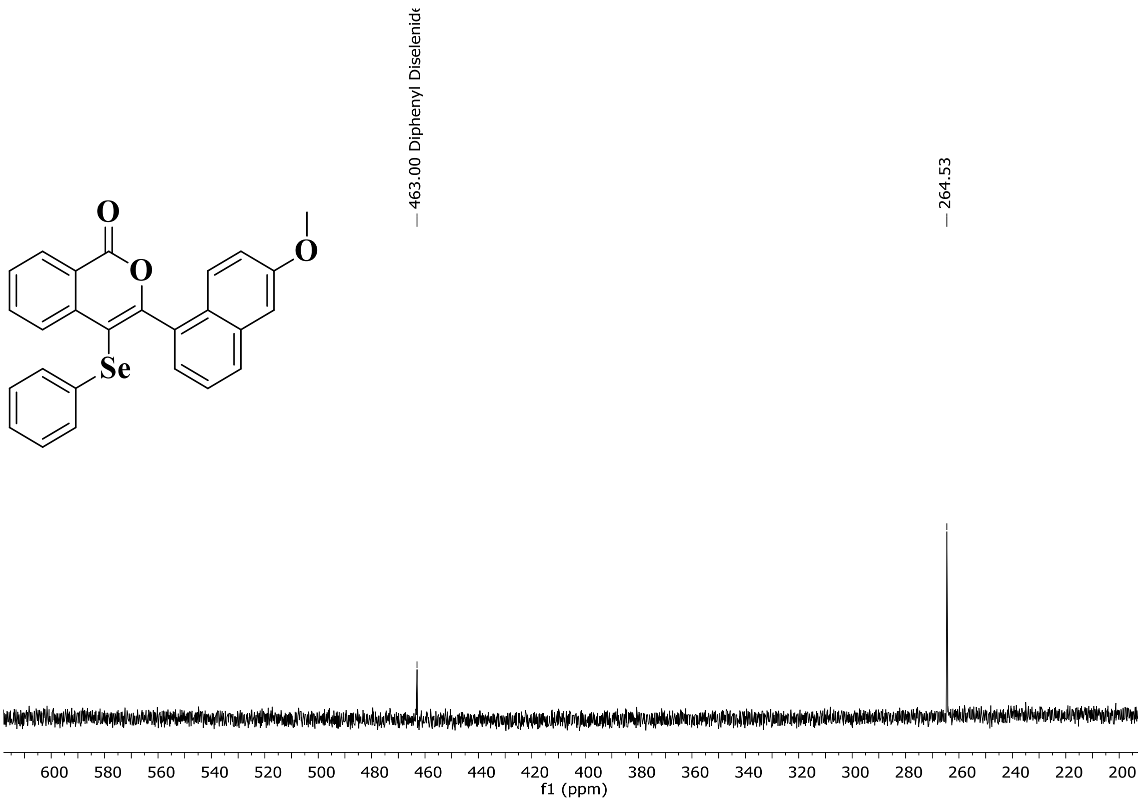


**Figure S45.** $^{77}$Se NMR spectrum (76 MHz) of compound **3m** obtained in $CDCl_3$.

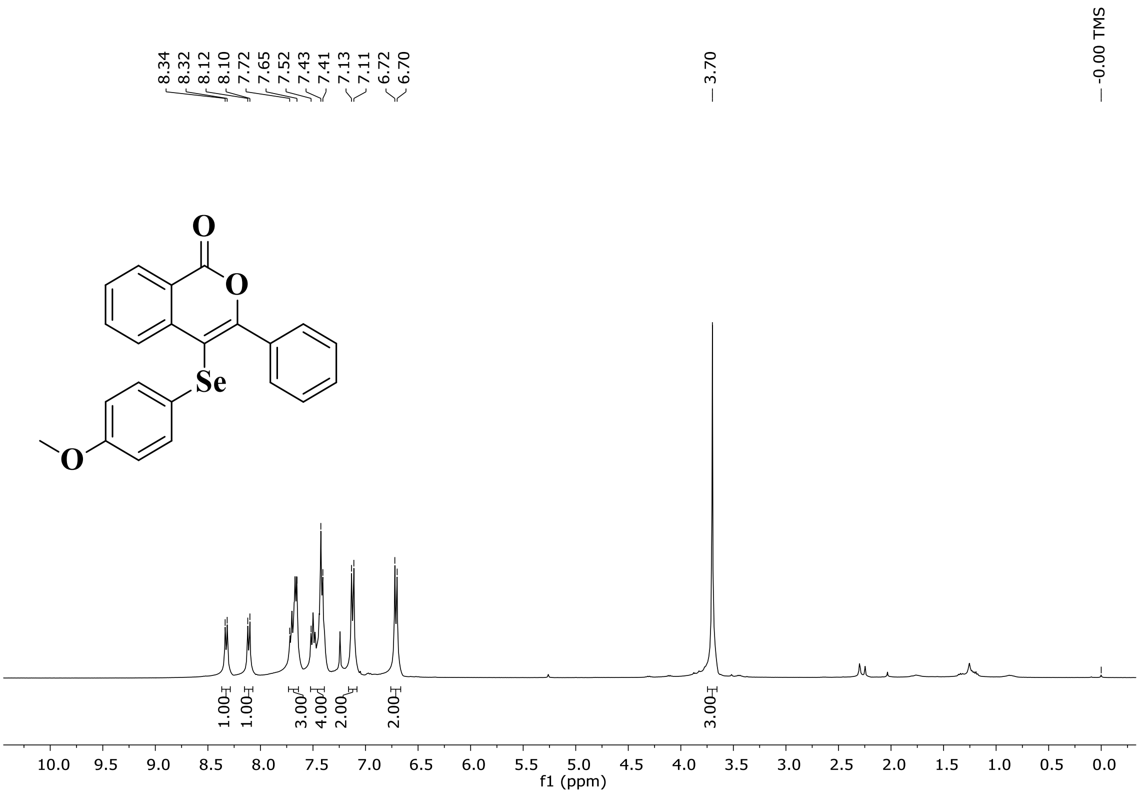


**Figure S46.** $^{1}$H NMR spectrum (400 MHz) of compound **3n** obtained in $CDCl_3$.

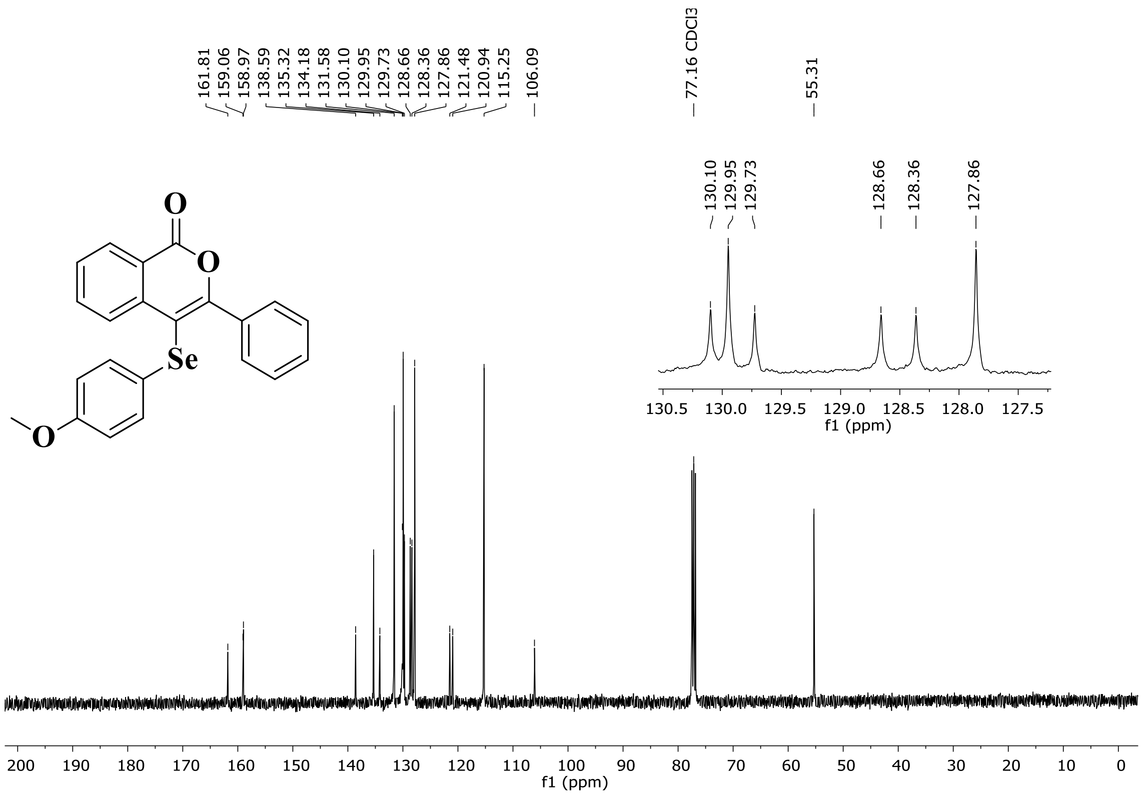


**Figure S47.** $^{13}C$ NMR spectrum (101 MHz) of compound **3n** obtained in $CDCl_3$.

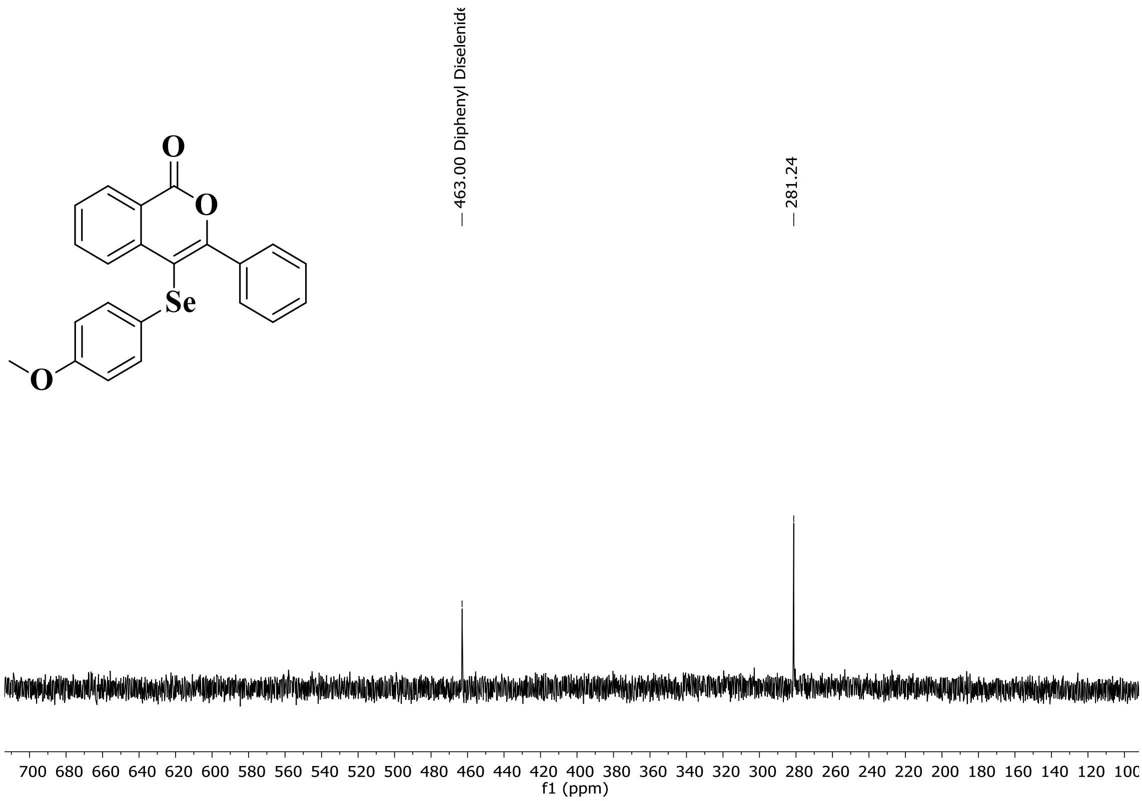


**Figure S48.** $^{77}Se$ NMR spectrum (76 MHz) of compound **3n** obtained in $CDCl_3$.

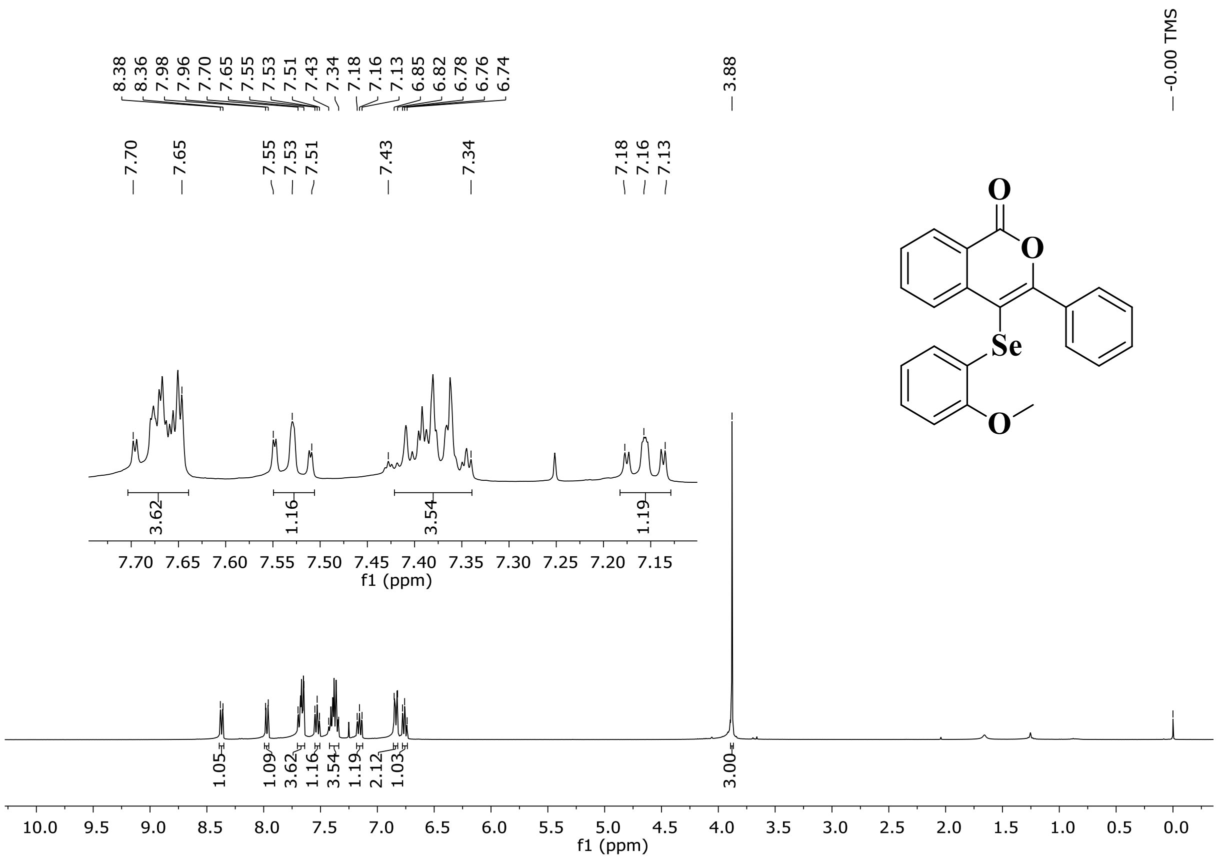

**Figure S49.** $^{1}H$ NMR spectrum (400 MHz) of compound **3o** obtained in $CDCl_3$.

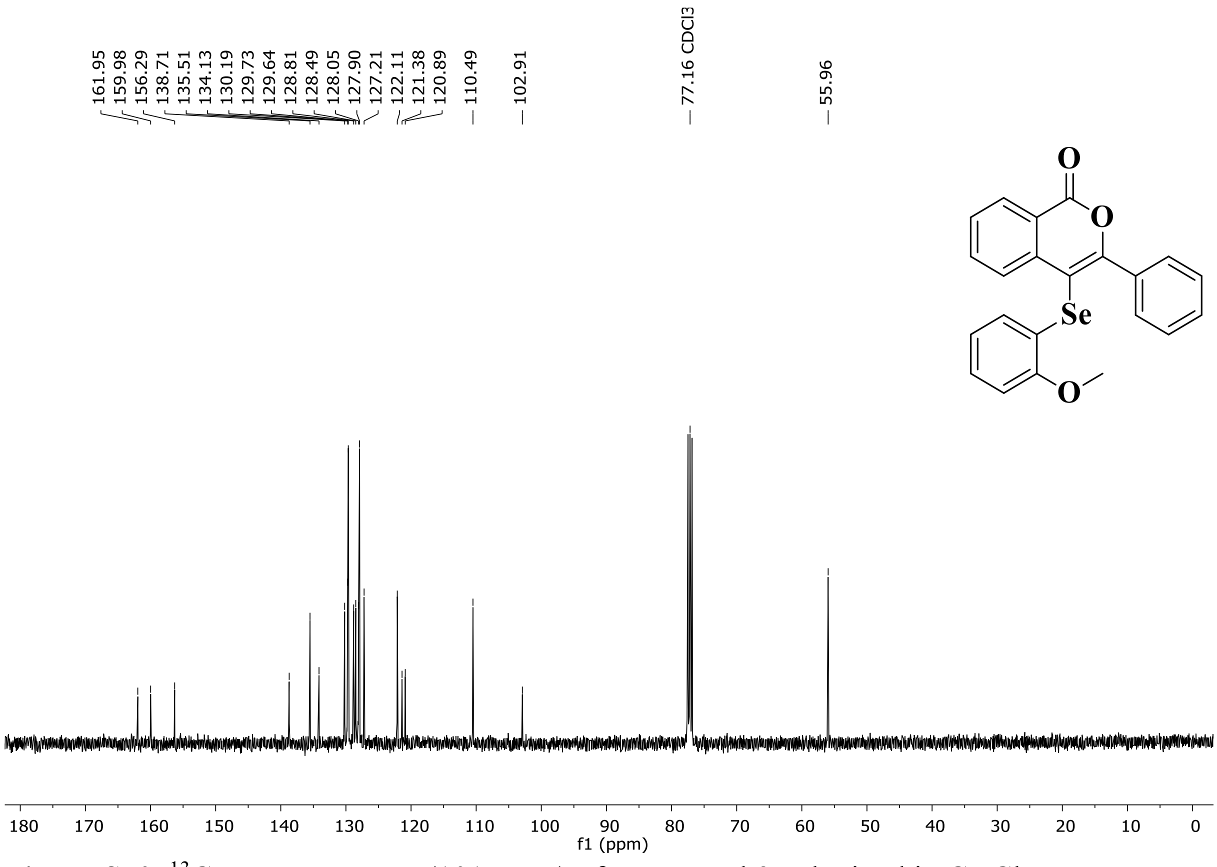

**Figure S50.** $^{13}C$ NMR spectrum (101 MHz) of compound **3o** obtained in $CDCl_3$.

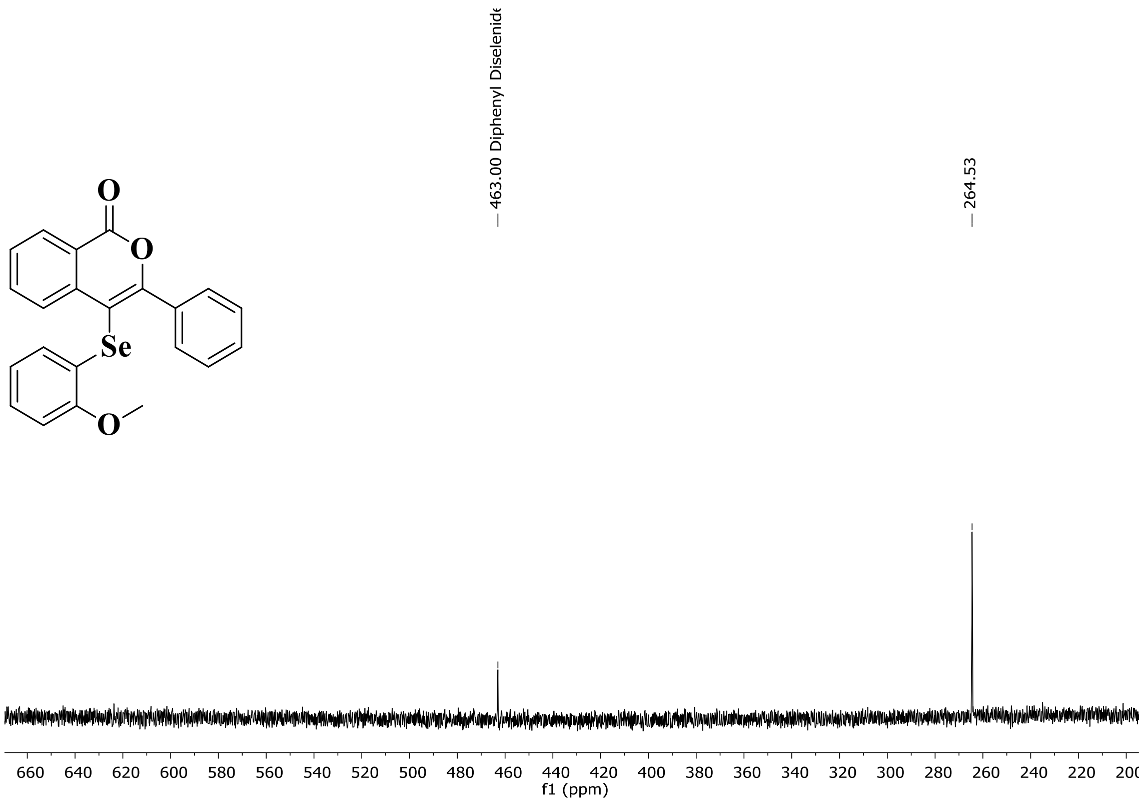


**Figure S51.** $^{77}$Se NMR spectrum (76 MHz) of compound **3o** obtained in $CDCl_3$.

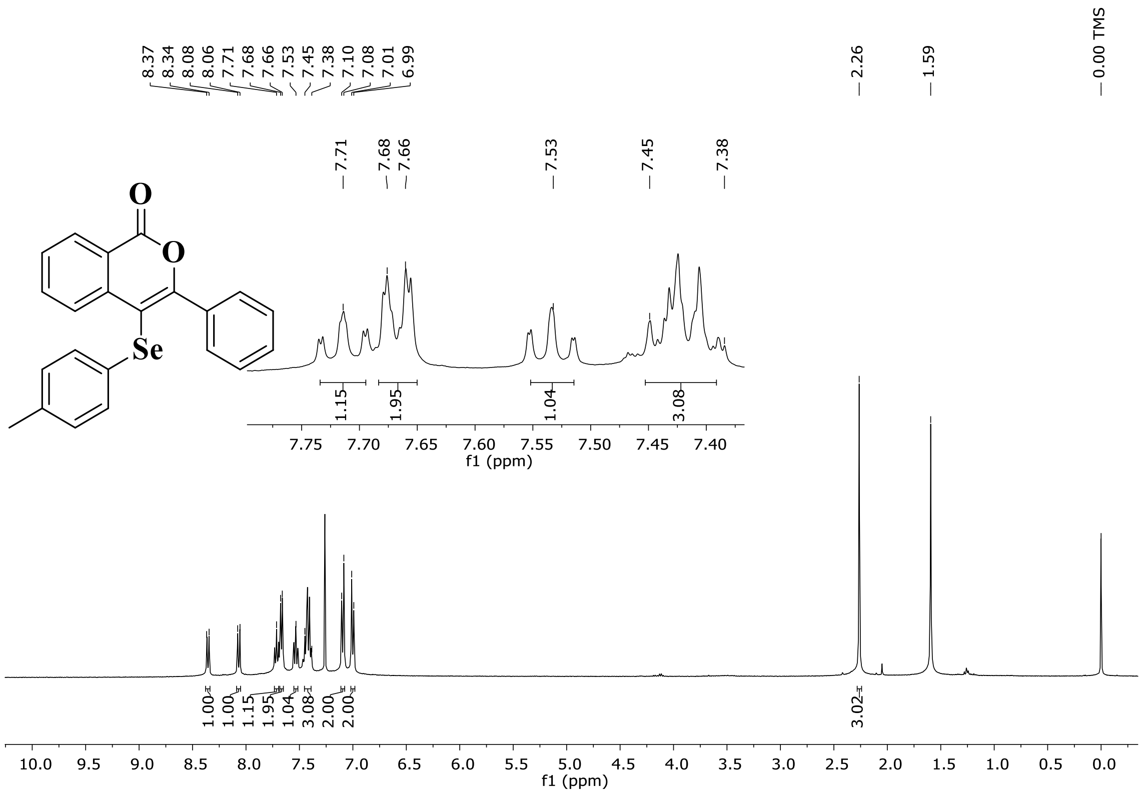


**Figure S52.** $^{1}$H NMR spectrum (400 MHz) of compound **3p** obtained in $CDCl_3$.

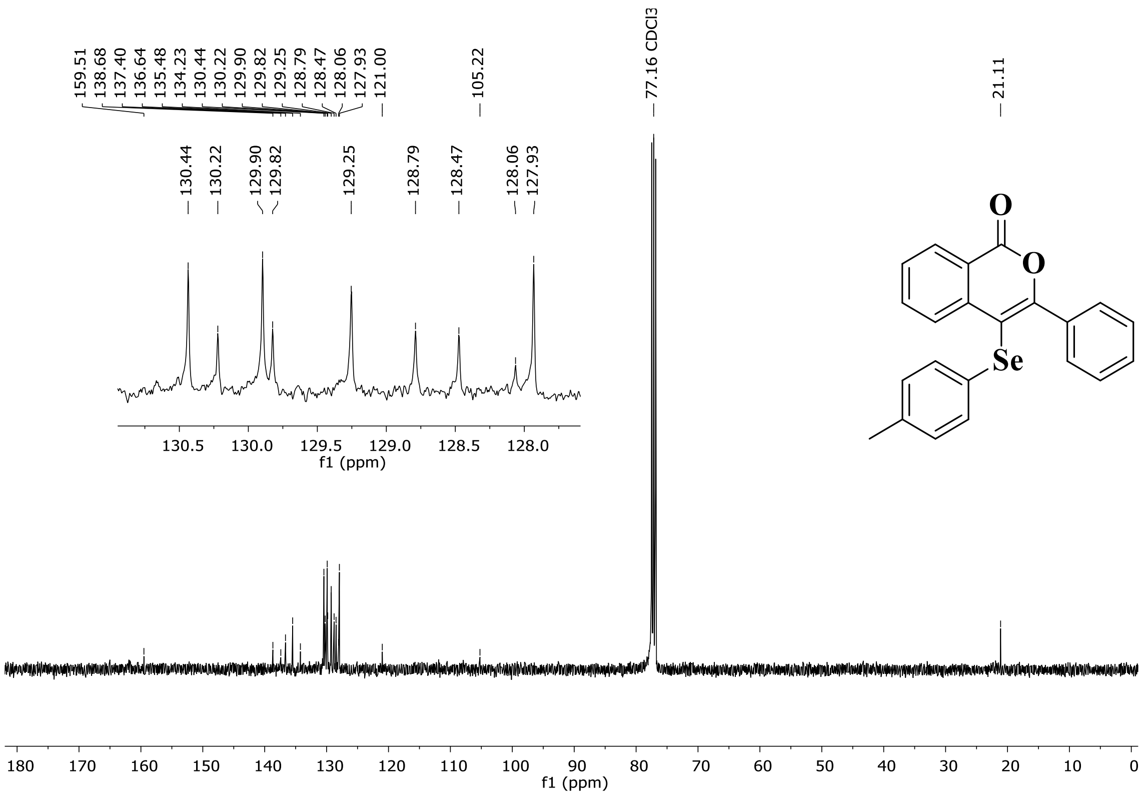


**Figure S53.** $^{13}$C NMR spectrum (101 MHz) of compound **3p** obtained in $CDCl_3$.

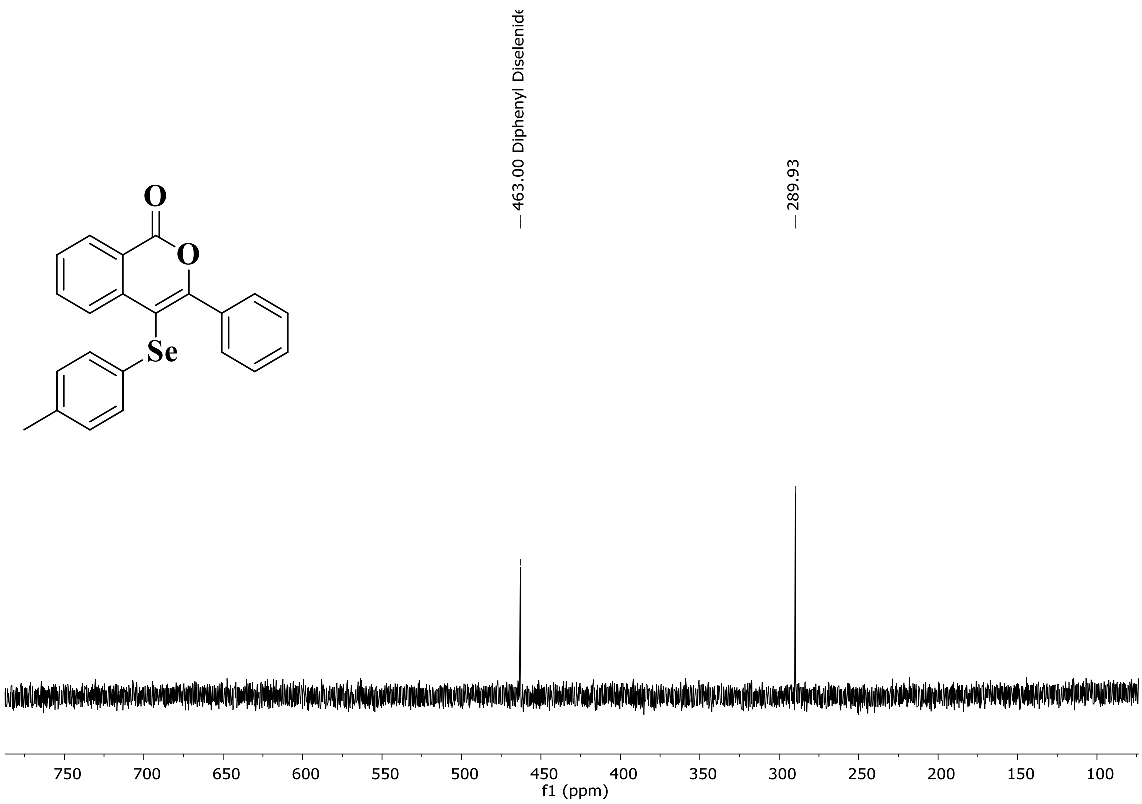


**Figure S54.** $^{77}$Se NMR spectrum (76 MHz) of compound **3p** obtained in $CDCl_3$.

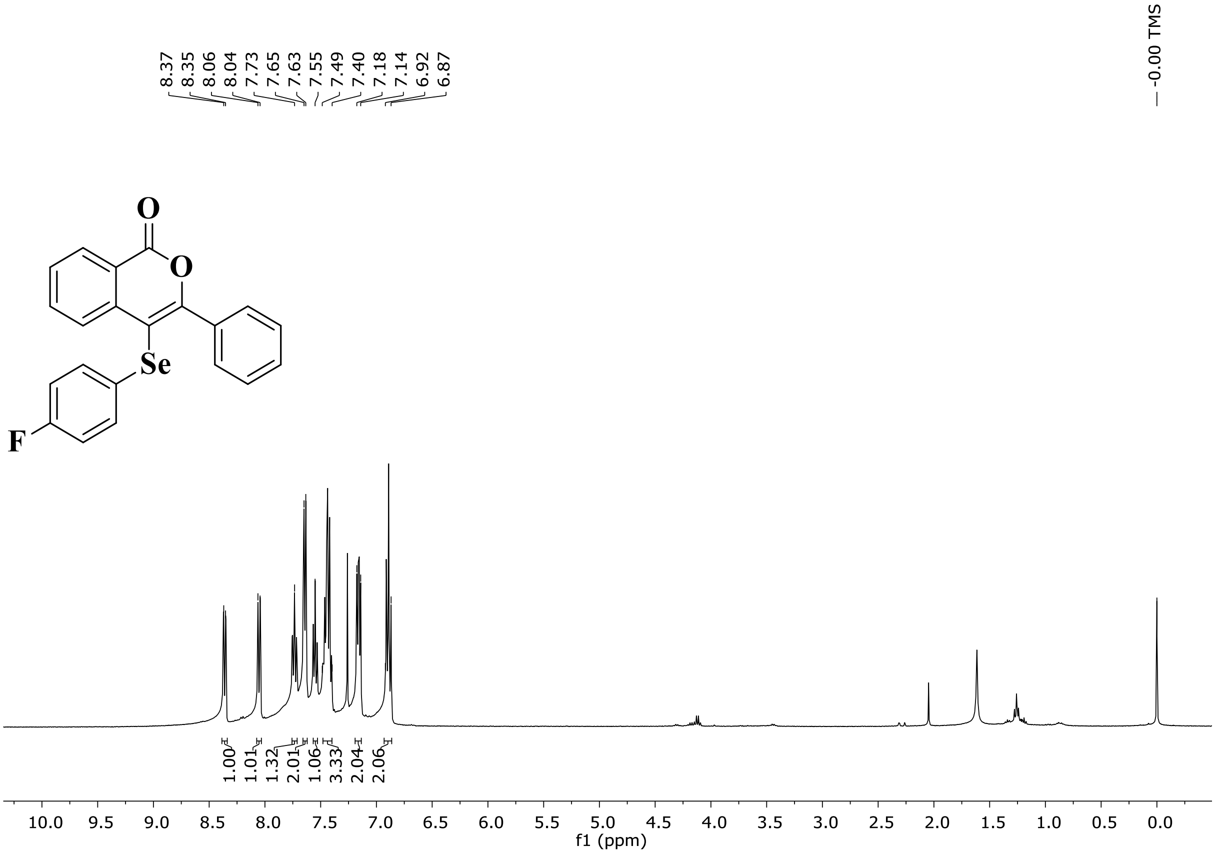


**Figure S55.** $^{1}$H NMR spectrum (400 MHz) of compound **3q** obtained in $CDCl_3$.

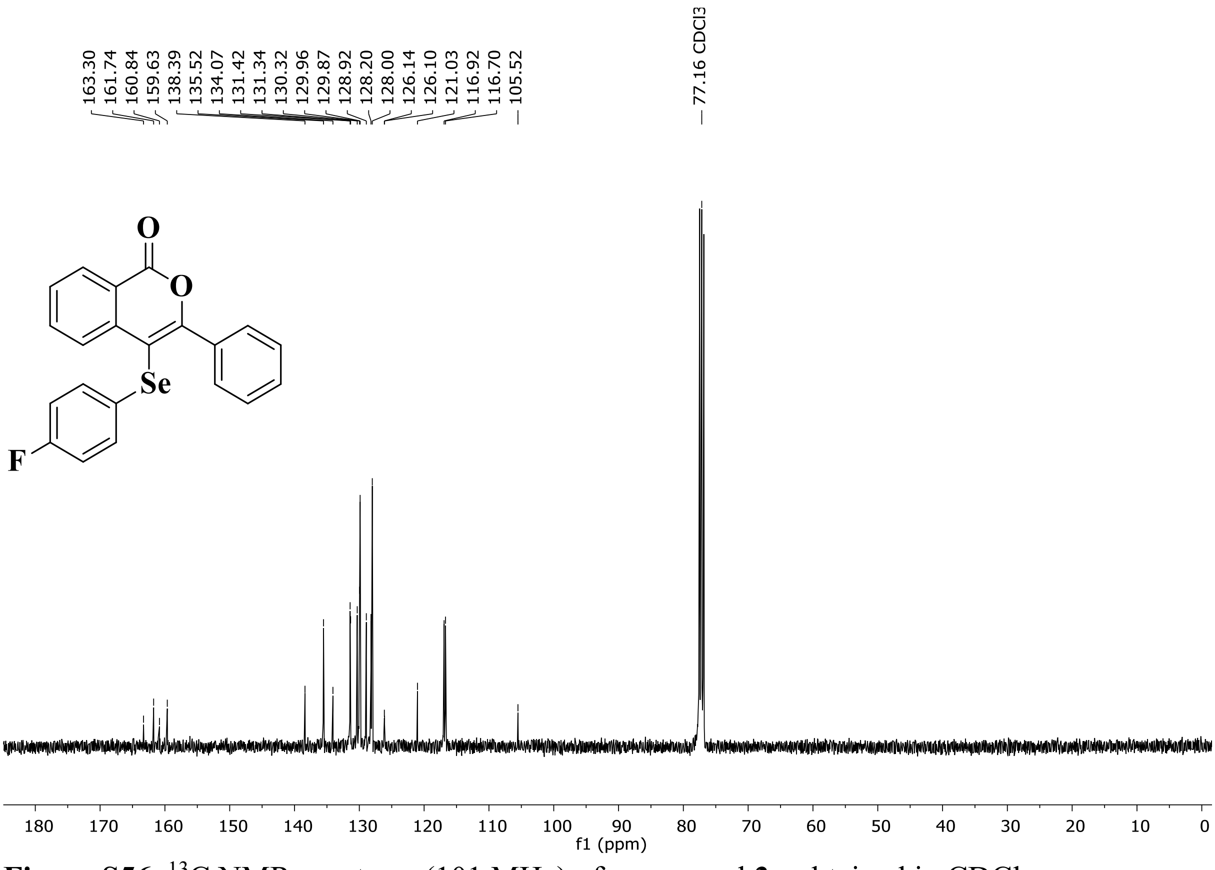


**Figure S56.** $^{13}$C NMR spectrum (101 MHz) of compound **3q** obtained in $CDCl_3$.

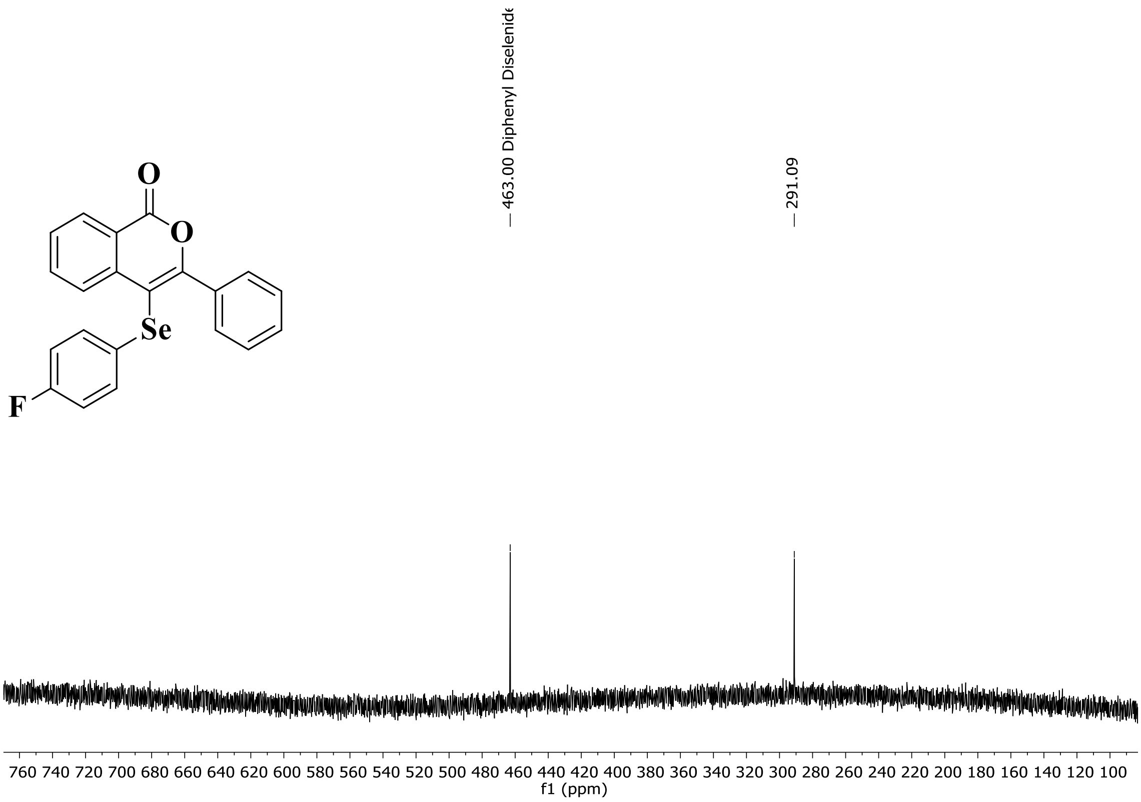


**Figure S57.** $^{77}$Se NMR spectrum (76 MHz) of compound **3q** obtained in $CDCl_3$.

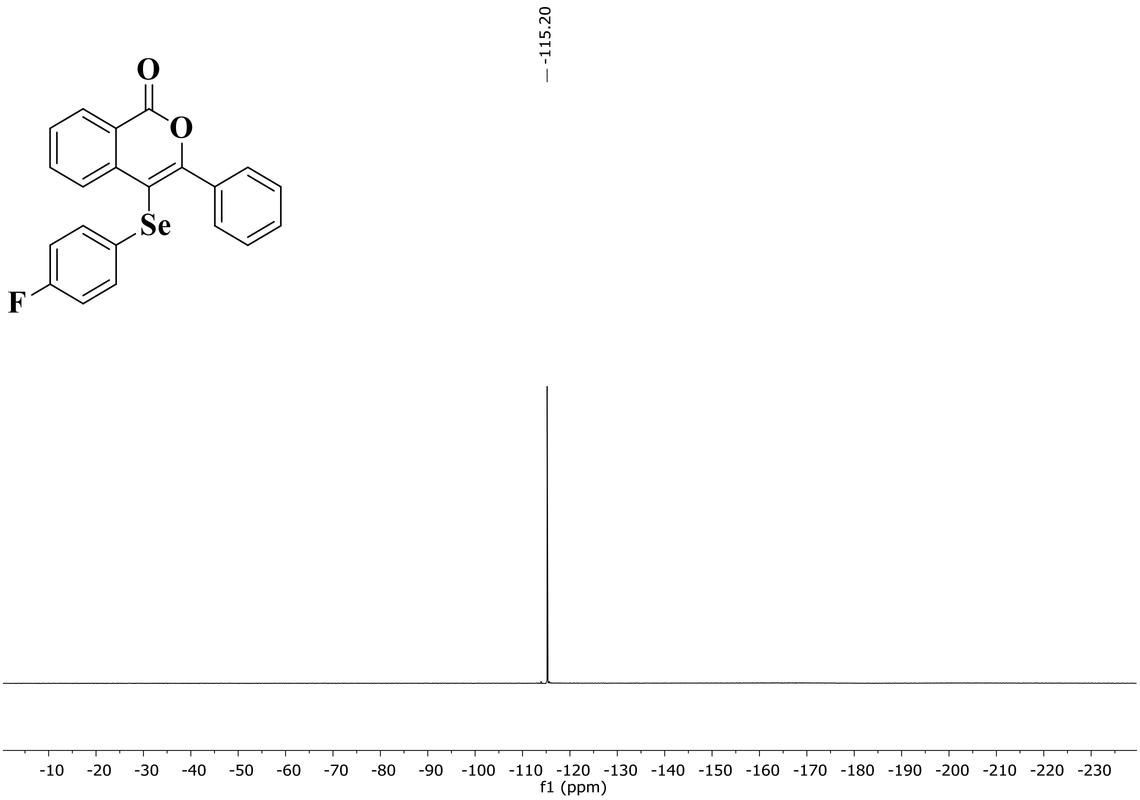


**Figure S58.** $^{19}$F NMR spectrum (376 MHz) of compound **3q** obtained in $CDCl_3$.

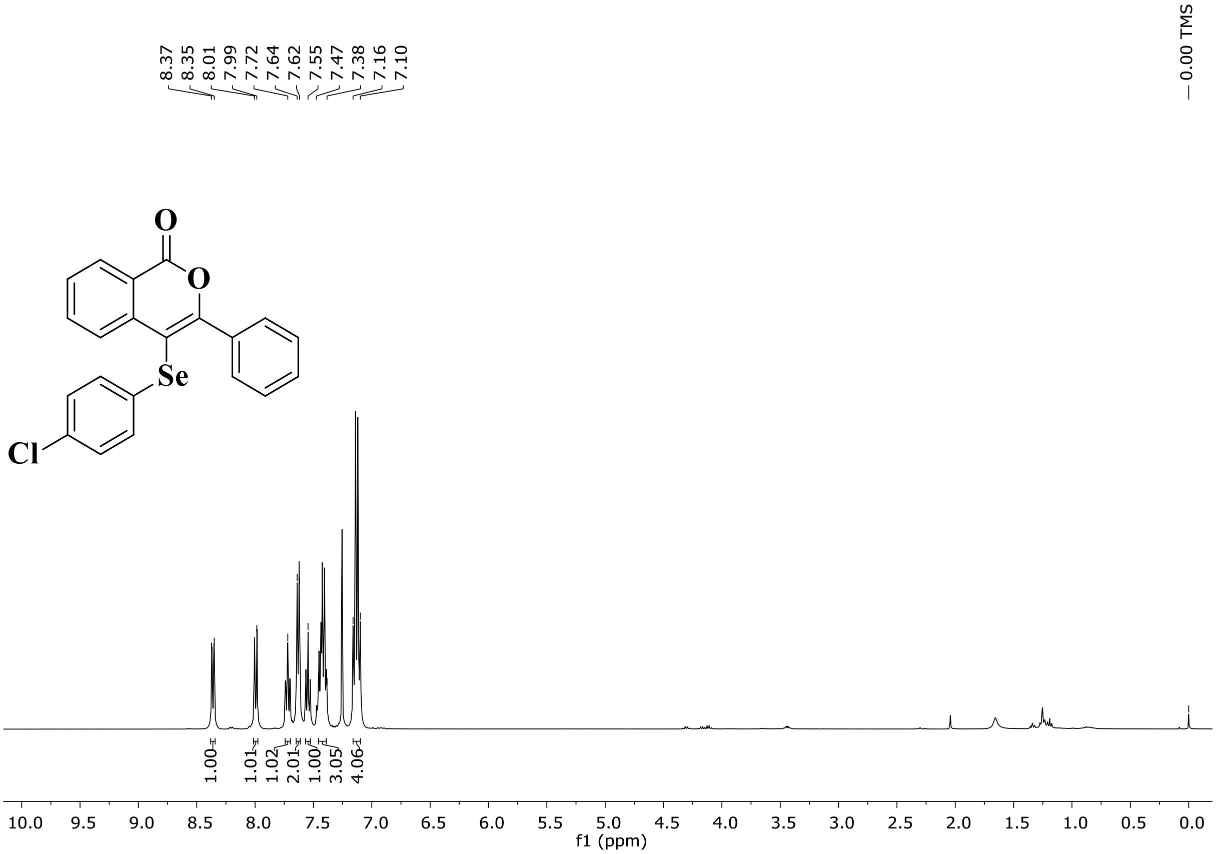


**Figure S59.** $^{1}H$ NMR spectrum (400 MHz) of compound **3r** obtained in $CDCl_3$.

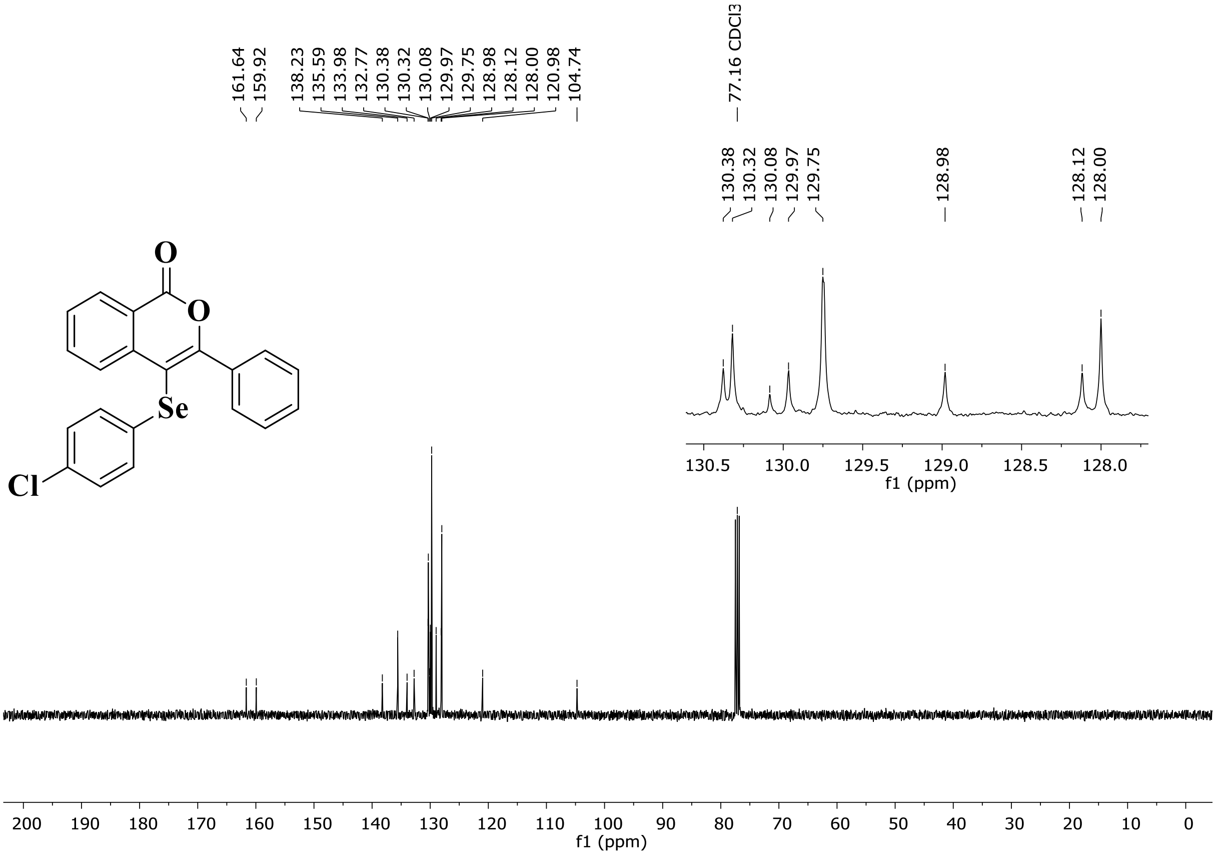


**Figure S60.** $^{13}C$ NMR spectrum (101 MHz) of compound **3r** obtained in $CDCl_3$.

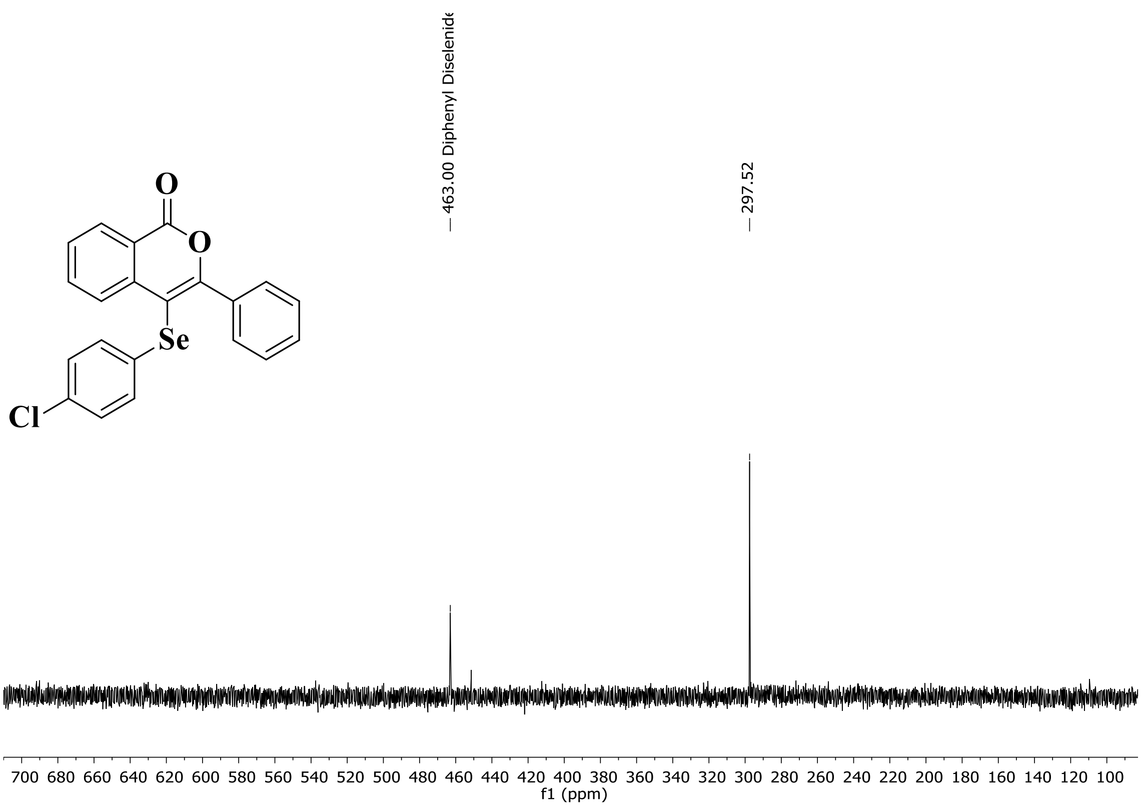


**Figure S61.** $^{77}$Se NMR spectrum (76 MHz) of compound **3r** obtained in $CDCl_3$.

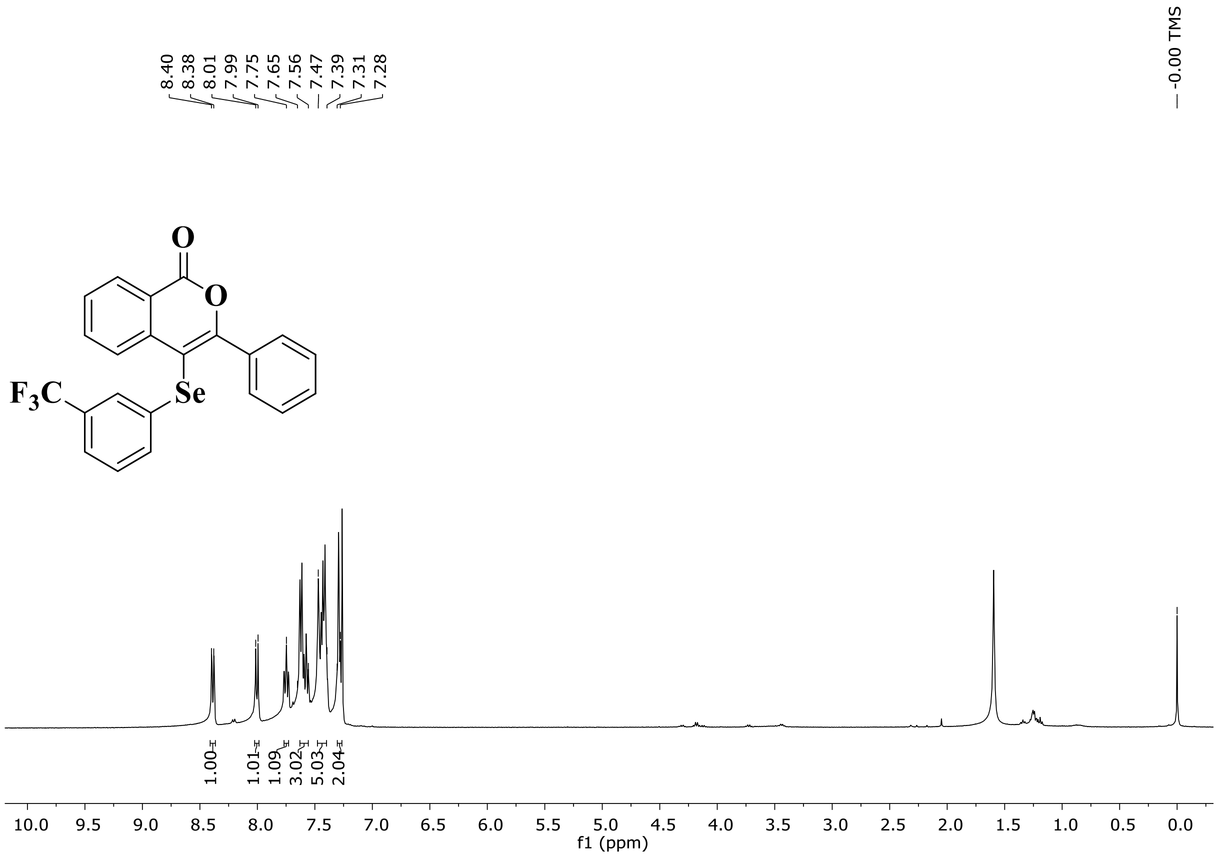


**Figure S62.** $^1$H NMR spectrum (400 MHz) of compound **3s** obtained in $CDCl_3$.

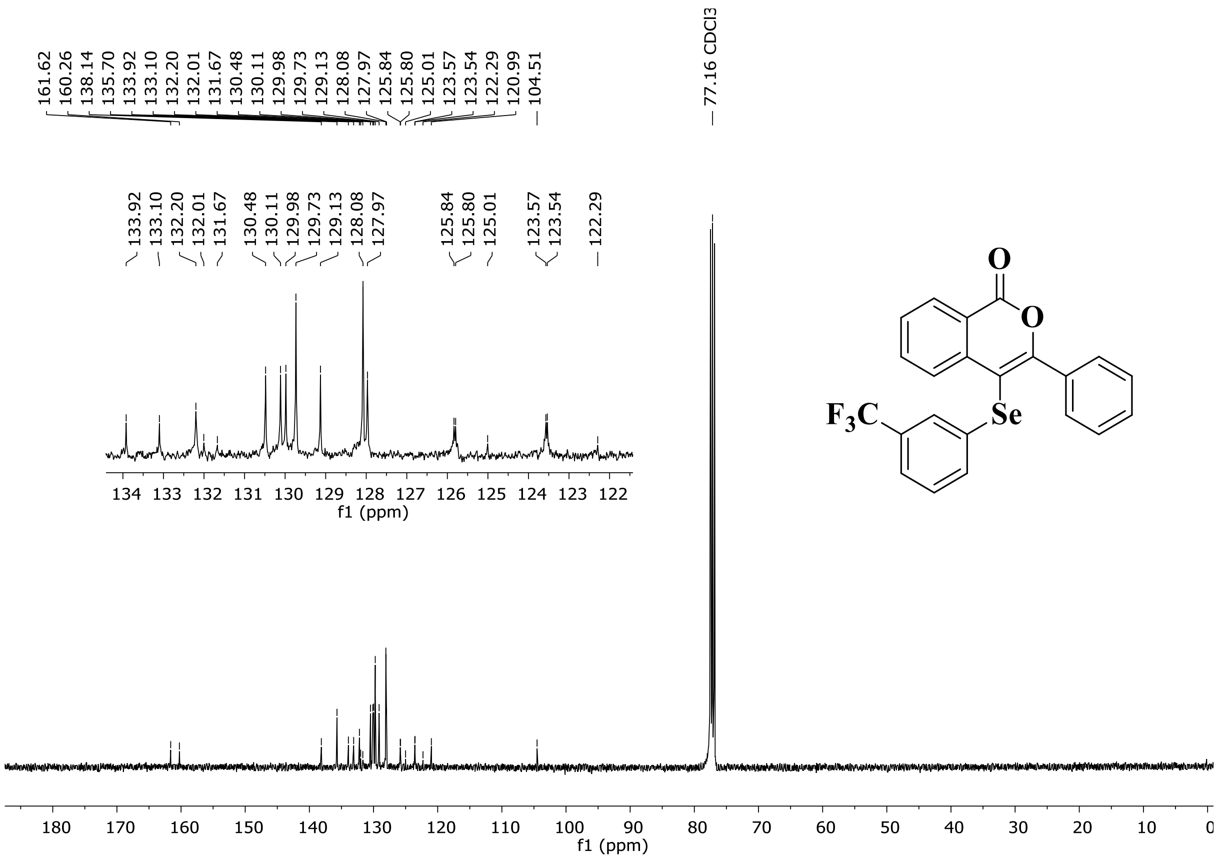


**Figure S63.** $^{13}$C NMR spectrum (101 MHz) of compound **3s** obtained in $CDCl_3$

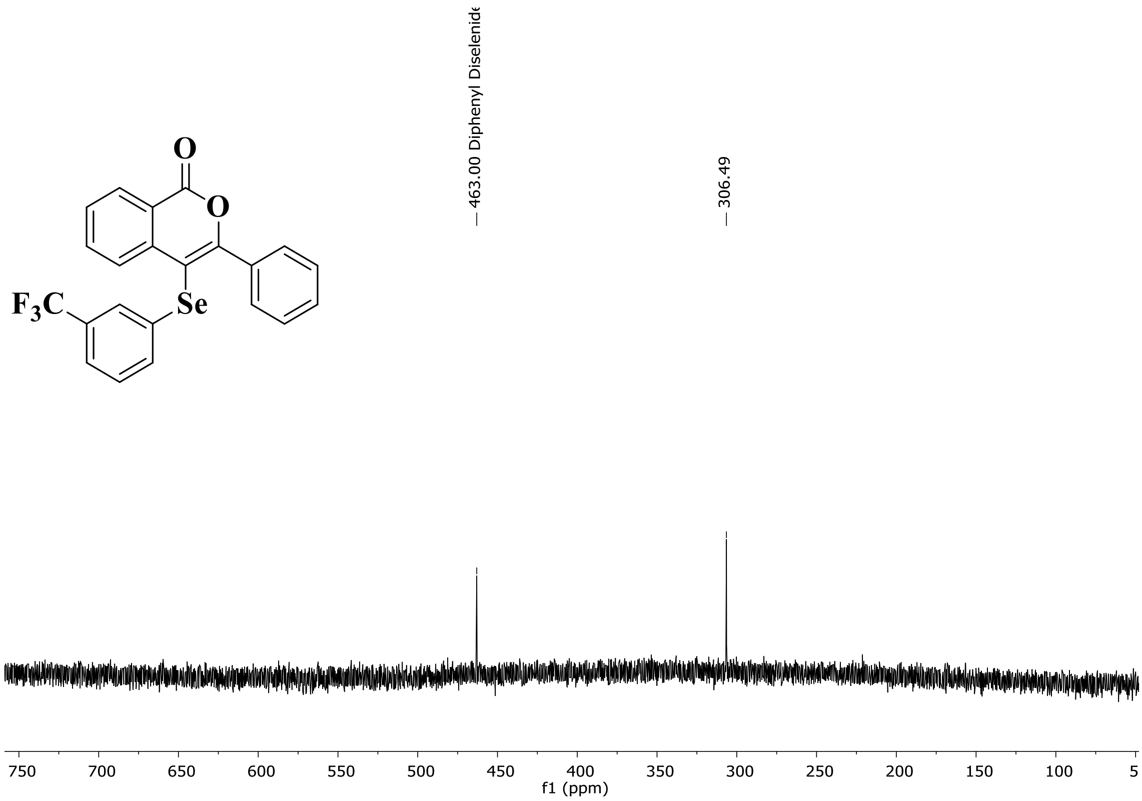


**Figure S64.** $^{77}$Se NMR spectrum (76 MHz) of compound **3s** obtained in $CDCl_3$.

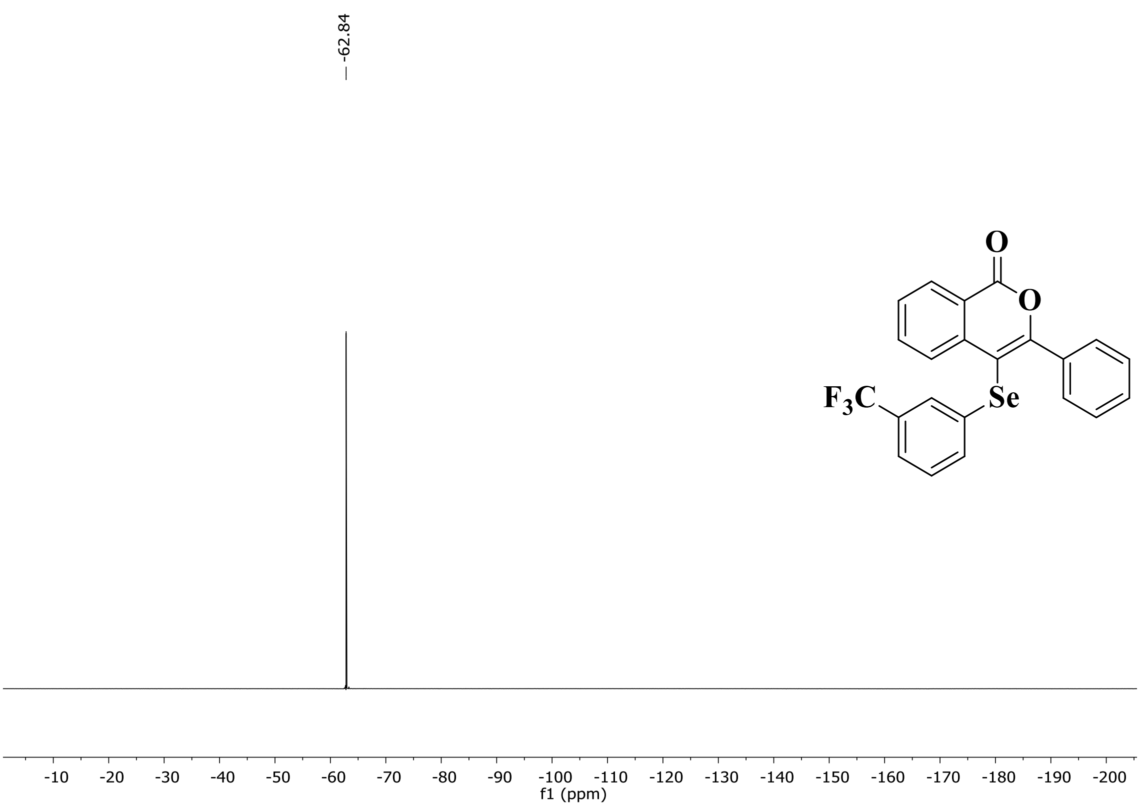


**Figure S65.** $^{19}F$ NMR spectrum (376 MHz) of compound **3s** obtained in $CDCl_3$.

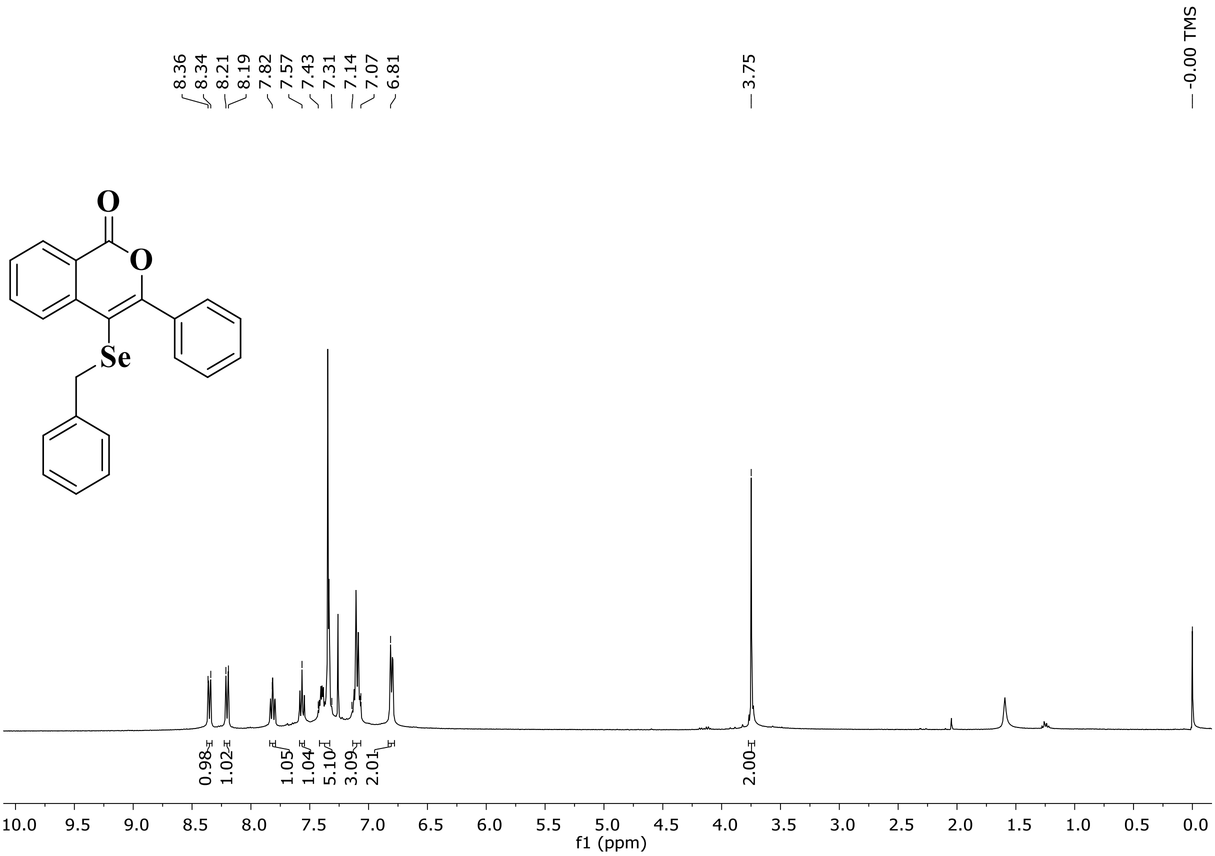


**Figure S66.** $^{1}H$ NMR spectrum (400 MHz) of compound **3t** obtained in $CDCl_3$.

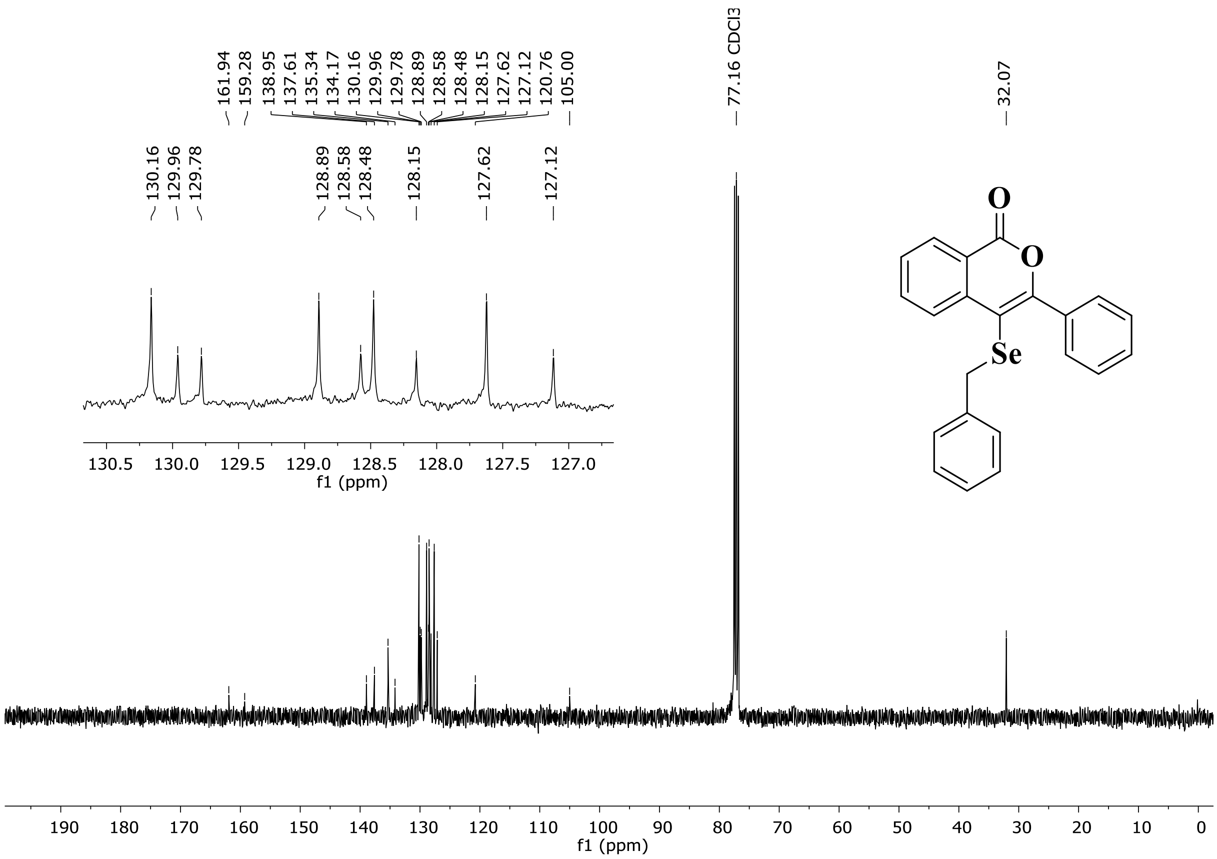


**Figure S67.** $^{13}C$ NMR spectrum (101 MHz) of compound **3t** obtained in $CDCl_3$

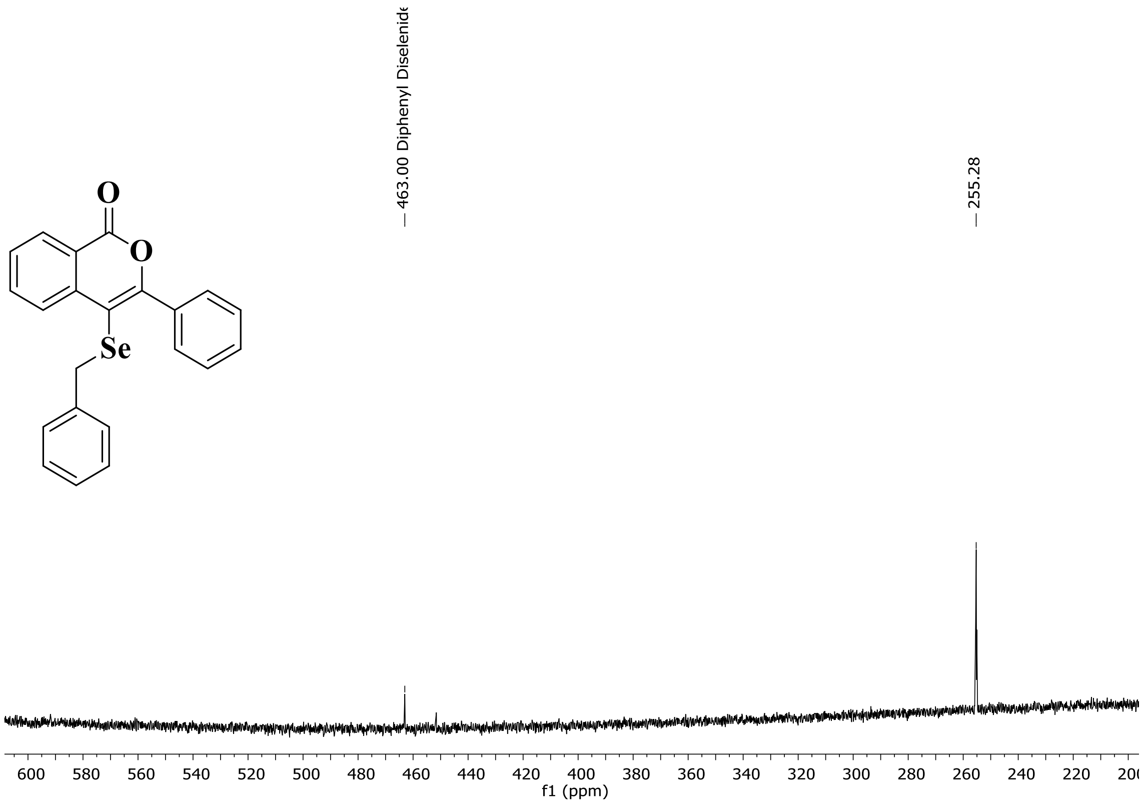


**Figure S68.** $^{77}Se$ NMR spectrum (76 MHz) of compound **3t** obtained in $CDCl_3$.

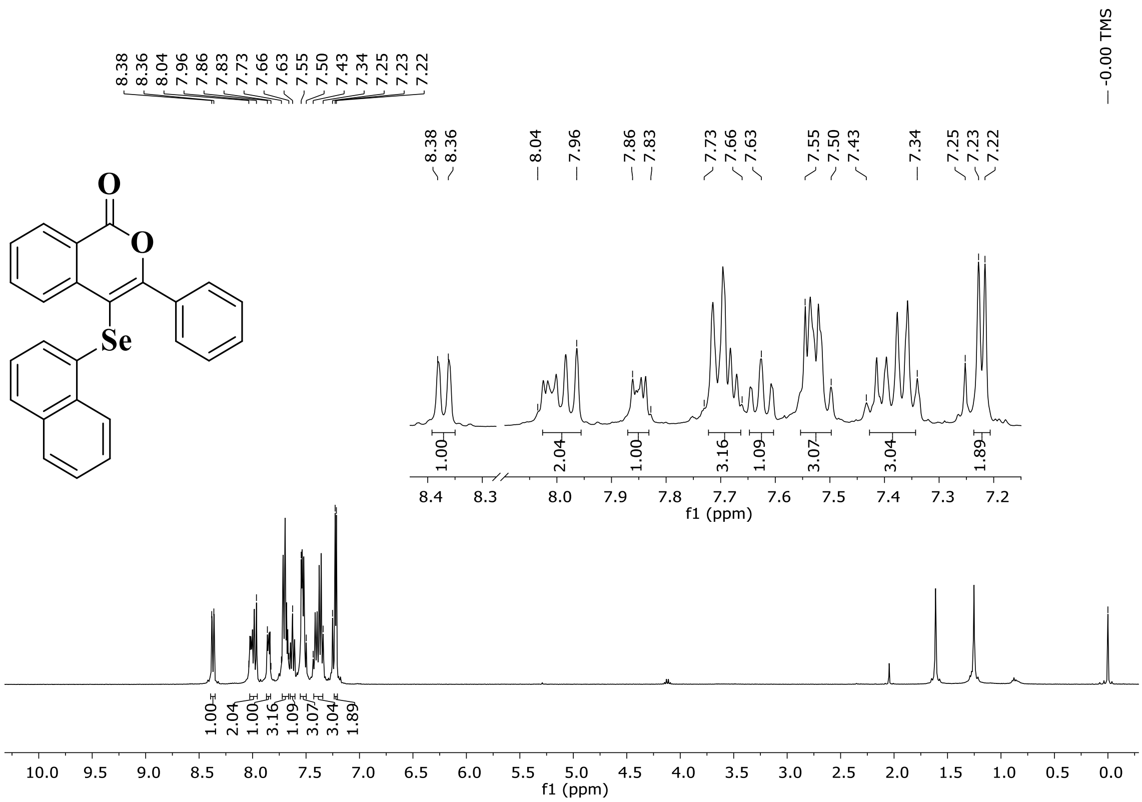

**Figure S69.** $^{1}$H NMR spectrum (400 MHz) of compound **3u** obtained in $CDCl_3$.

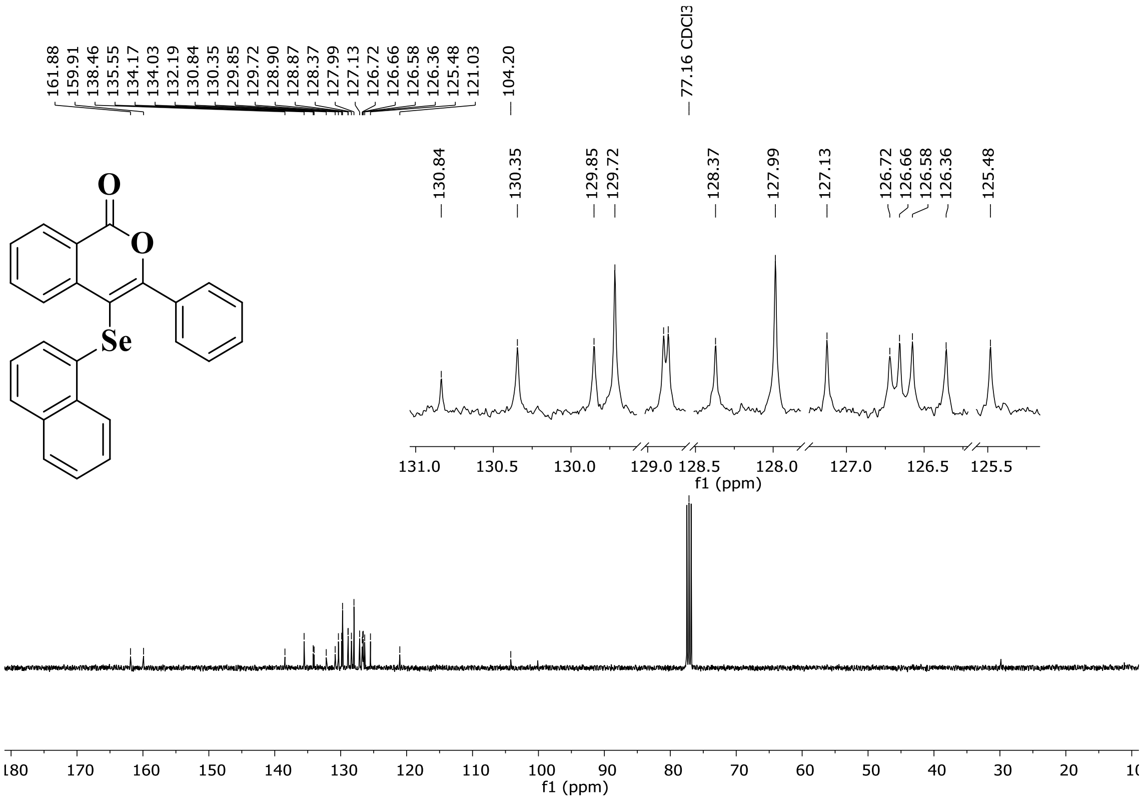

**Figure S70.** $^{13}$C NMR spectrum (101 MHz) of compound **3u** obtained in $CDCl_3$.

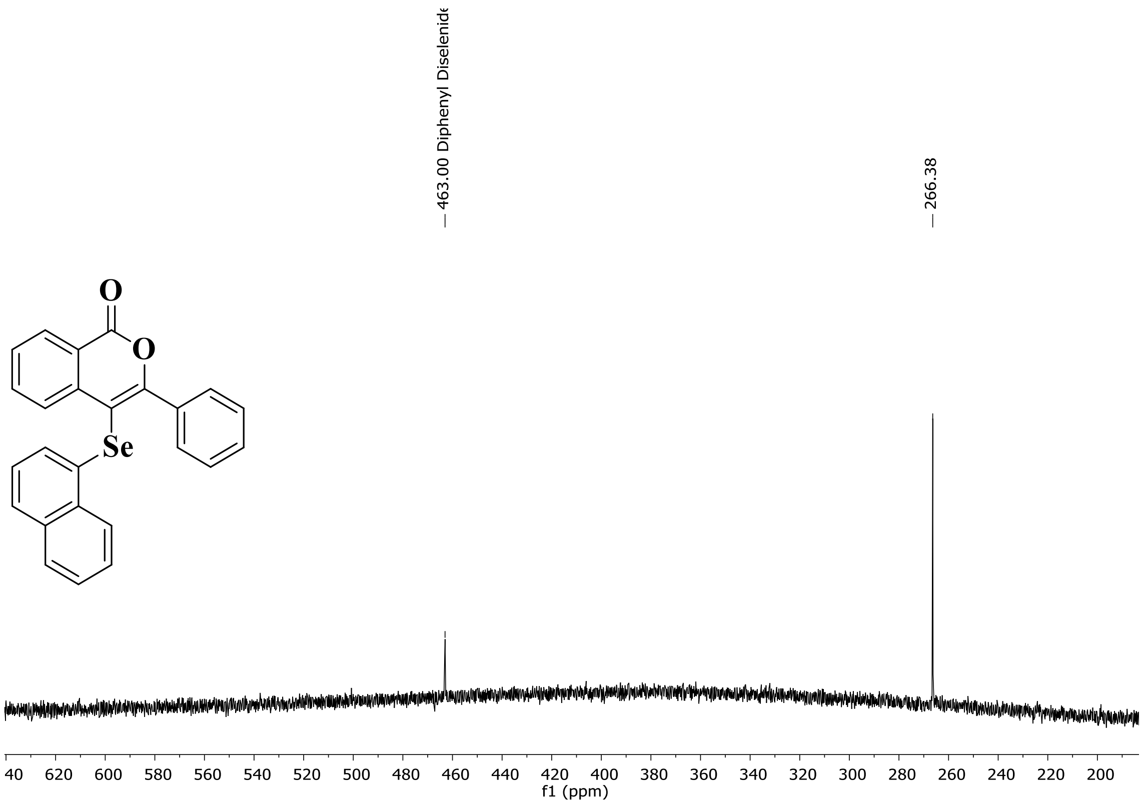


**Figure S71.** $^{77}$Se NMR spectrum (76 MHz) of compound **3u** obtained in $CDCl_3$.

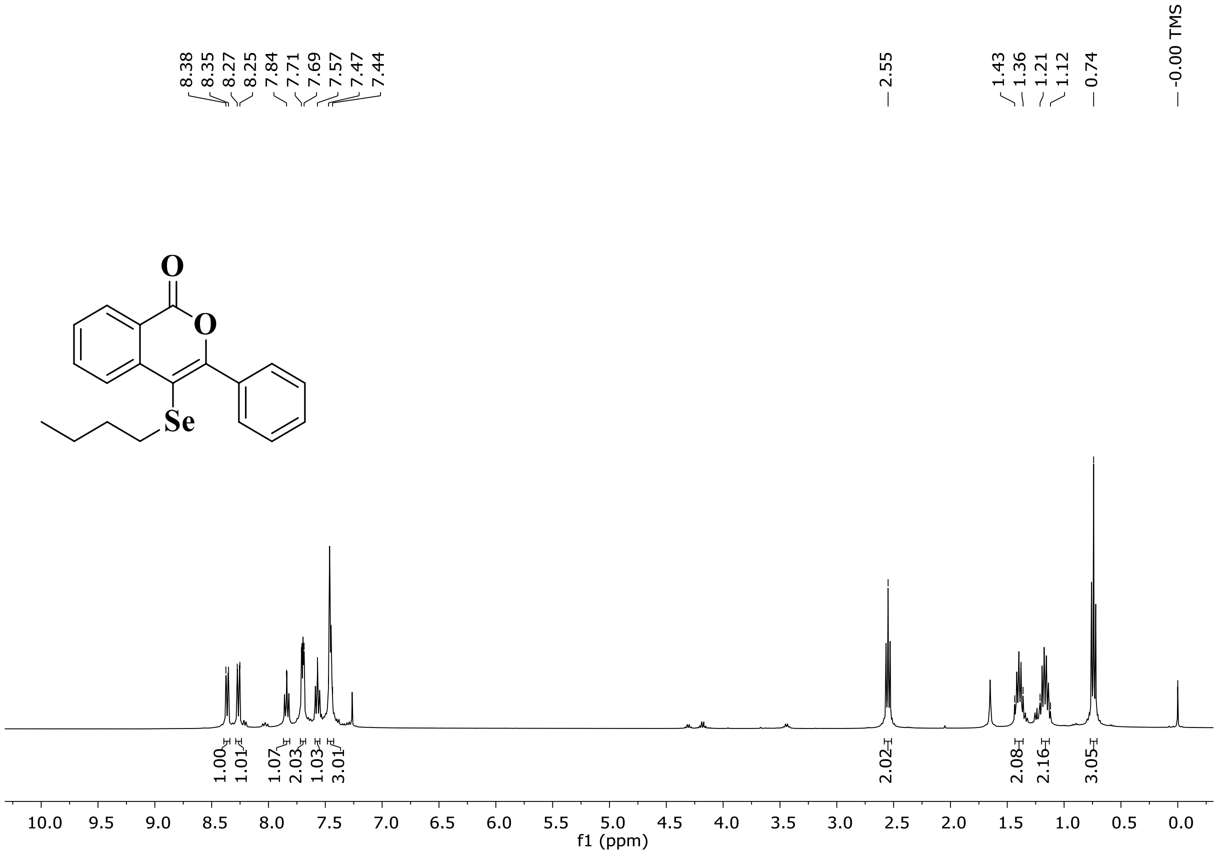


**Figure S72.** $^{1}$H NMR spectrum (400 MHz) of compound **3v** obtained in $CDCl_3$.

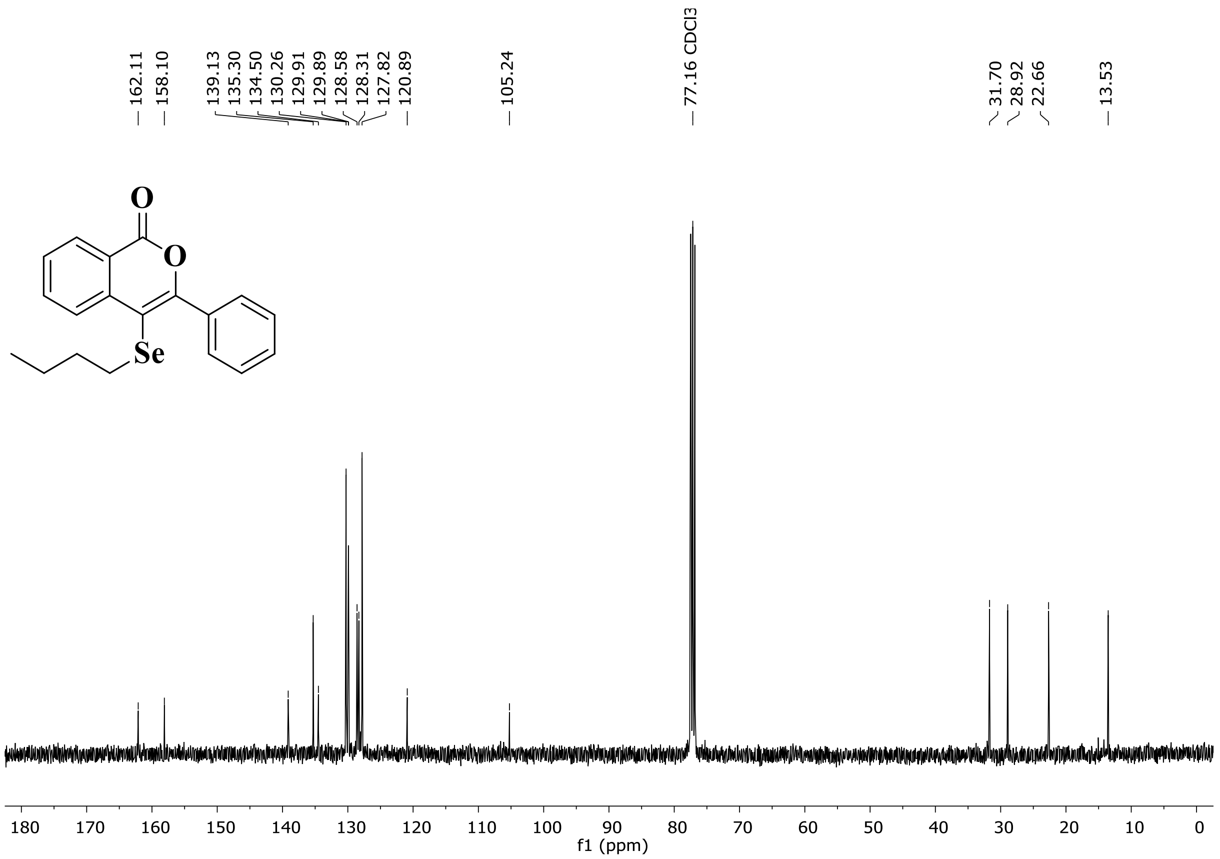


**Figure S73.** $^{13}$C NMR spectrum (101 MHz) of compound **3v** obtained in $CDCl_3$.

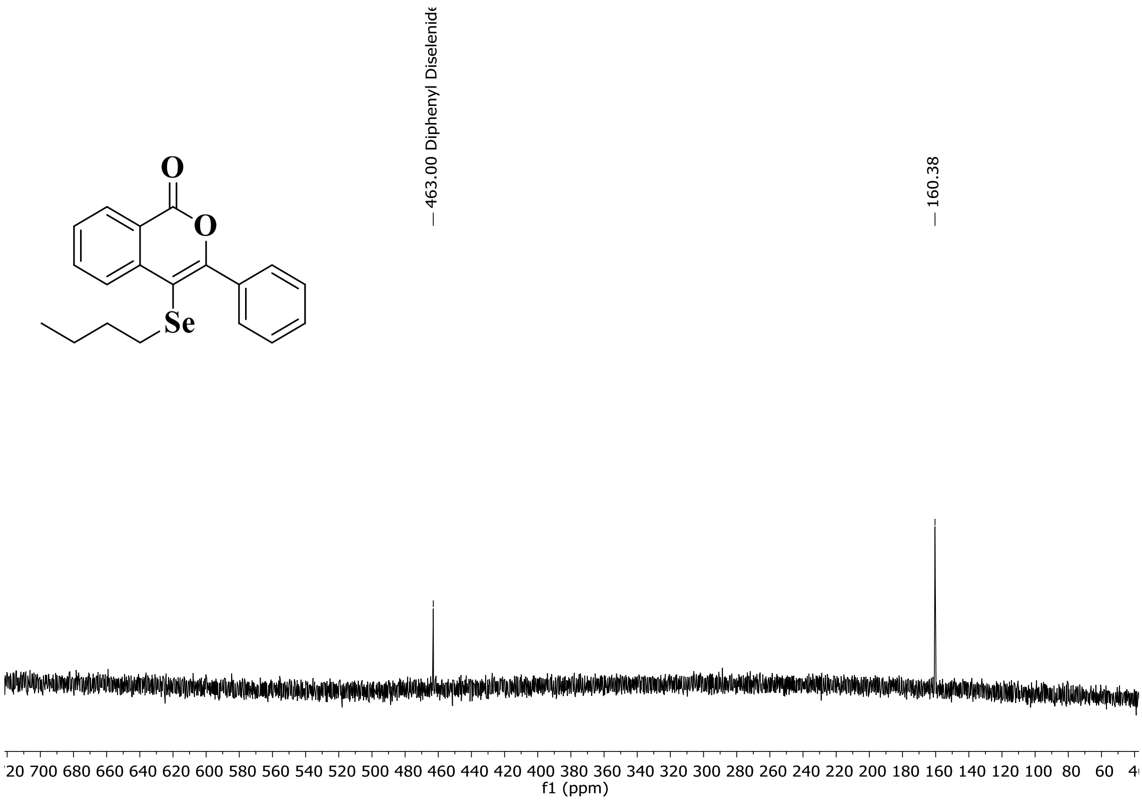


**Figure S74.** $^{77}$Se NMR spectrum (76 MHz) of compound **3v** obtained in $CDCl_3$.

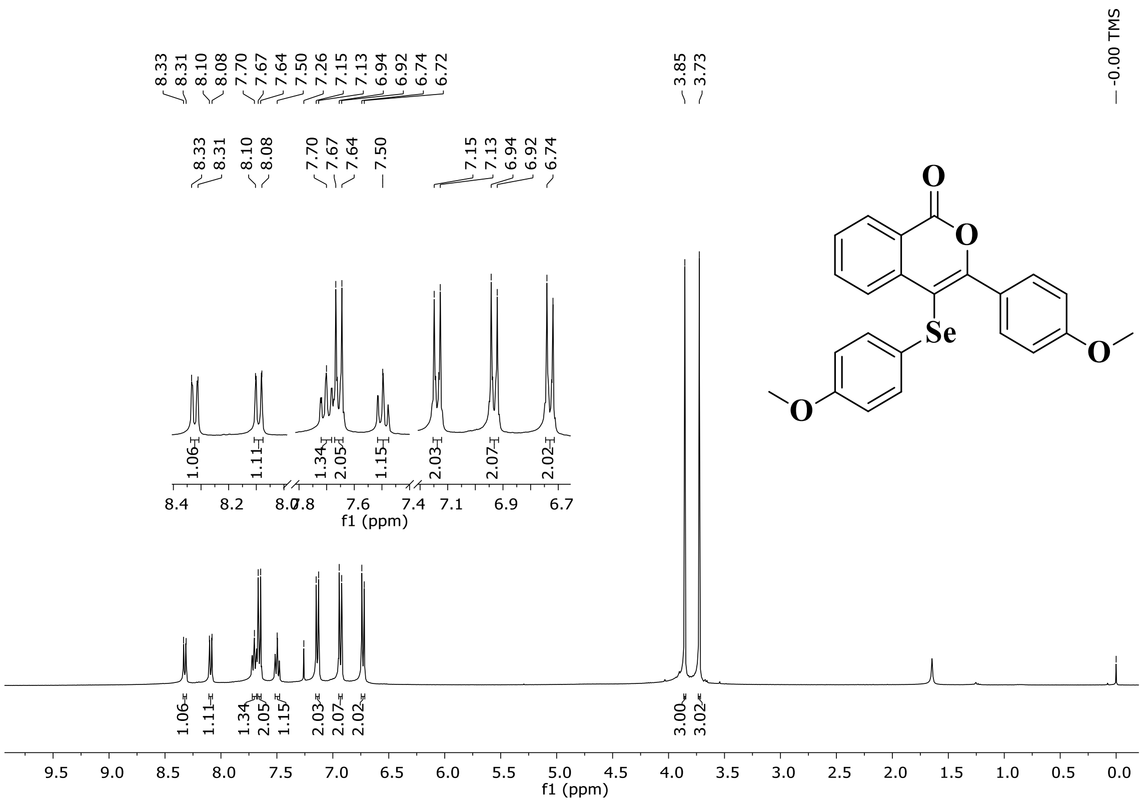

**Figure S75.** $^{1}H$ NMR spectrum (400 MHz) of compound **3w** obtained in $CDCl_3$.

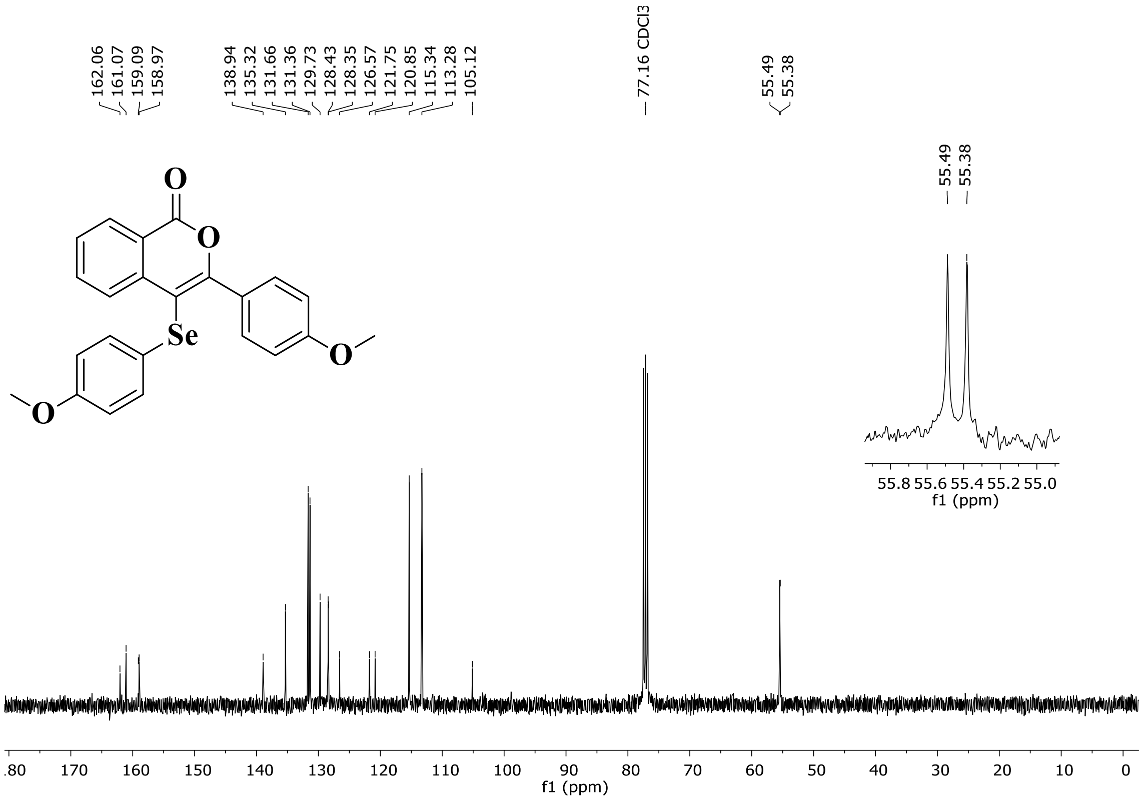

**Figure S76.** $^{13}C$ NMR spectrum (101 MHz) of compound **3w** obtained in $CDCl_3$.

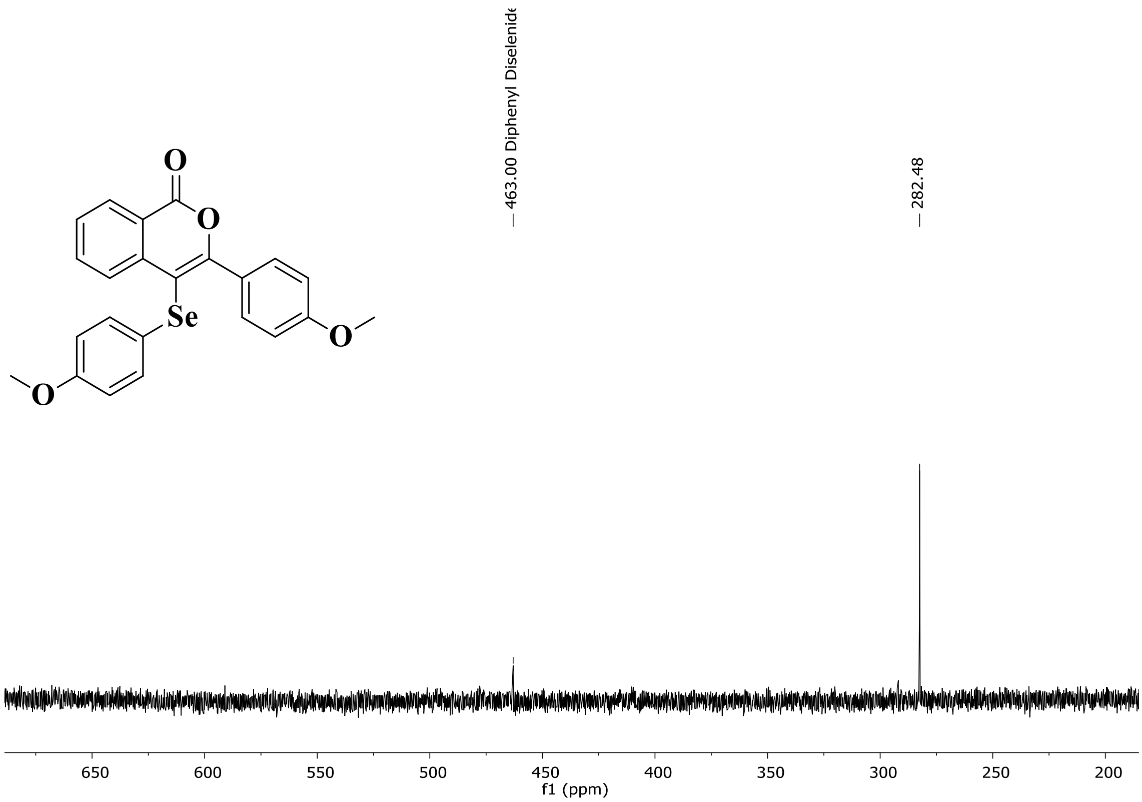


**Figure S77.** $^{77}$Se NMR spectrum (76 MHz) of compound **3w** obtained in $CDCl_3$.

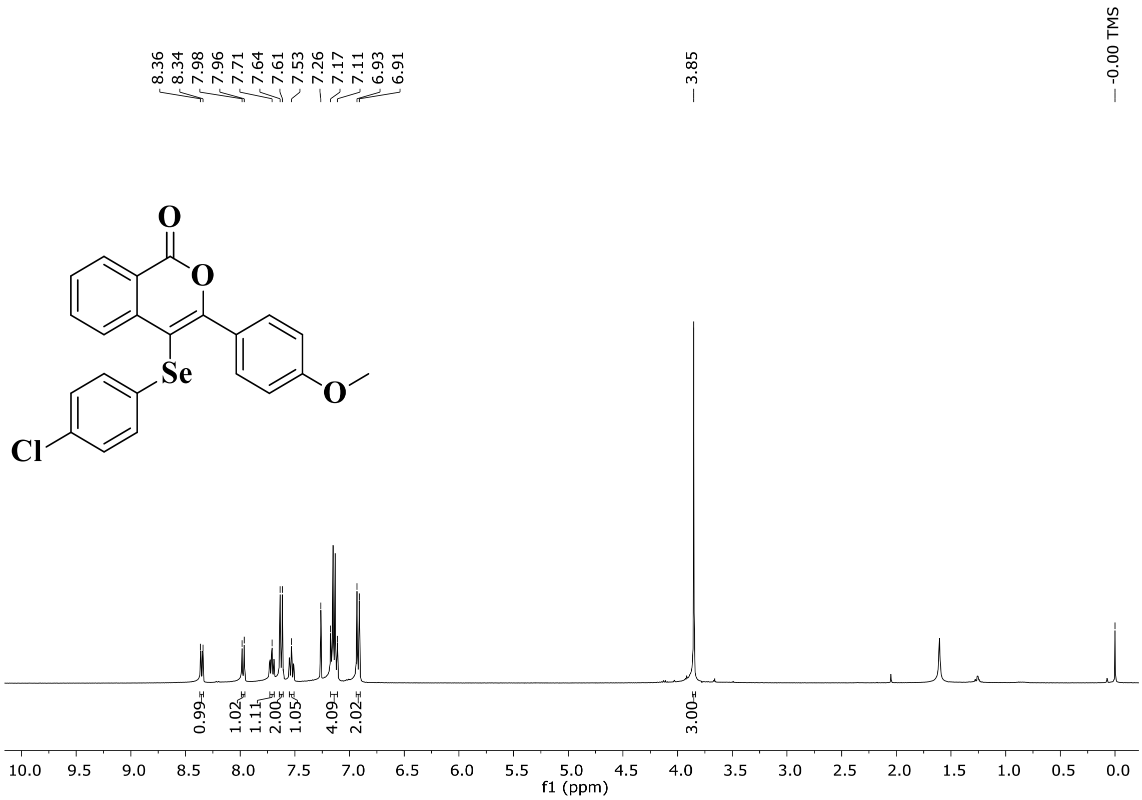


**Figure S78.** $^{1}$H NMR spectrum (400 MHz) of compound **3x** obtained in $CDCl_3$.

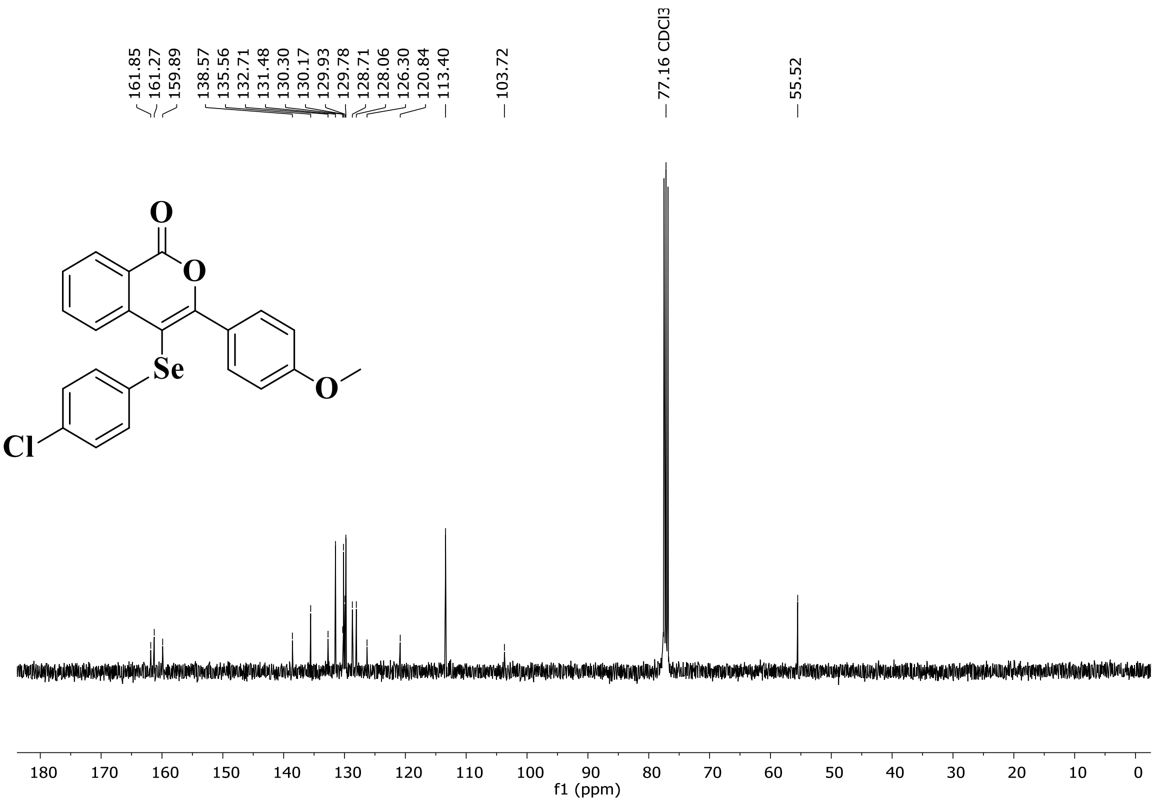


**Figure S79.** $^{13}$C NMR spectrum (101 MHz) of compound **3x** obtained in $CDCl_3$.

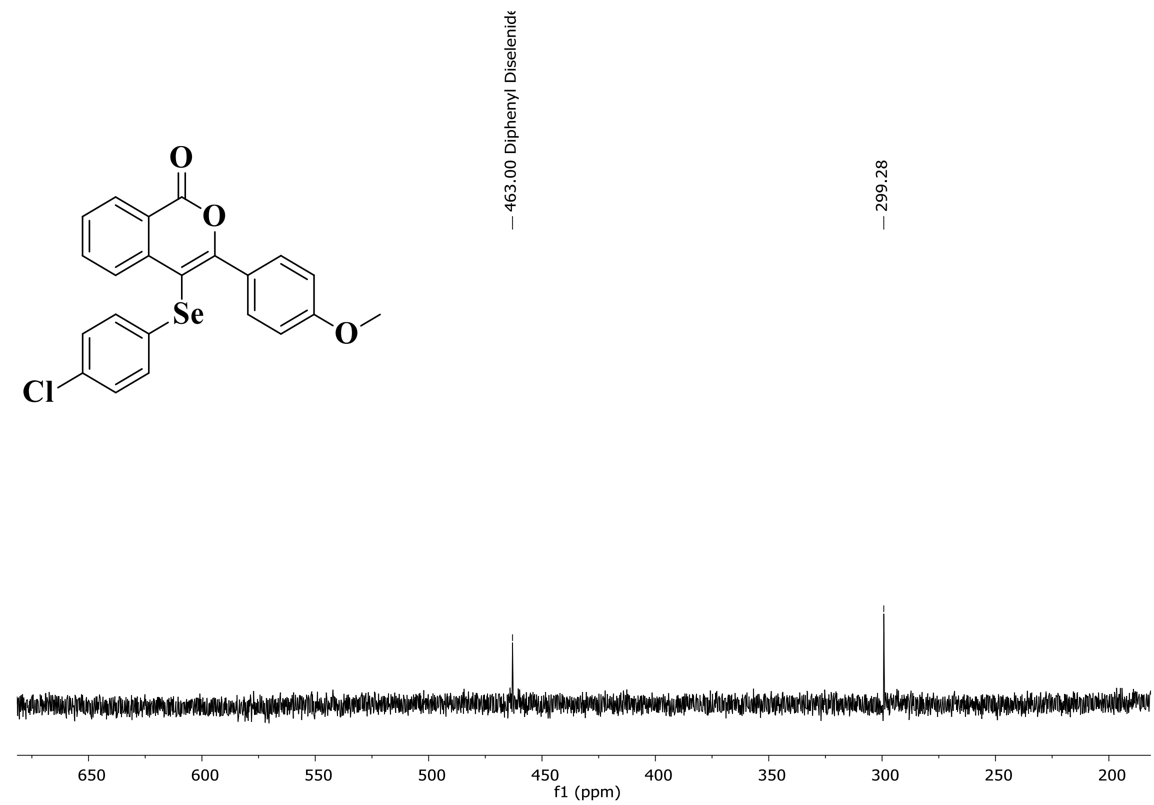


**Figure S80.** $^{77}$Se NMR spectrum (76 MHz) of compound **3x** obtained in $CDCl_3$.

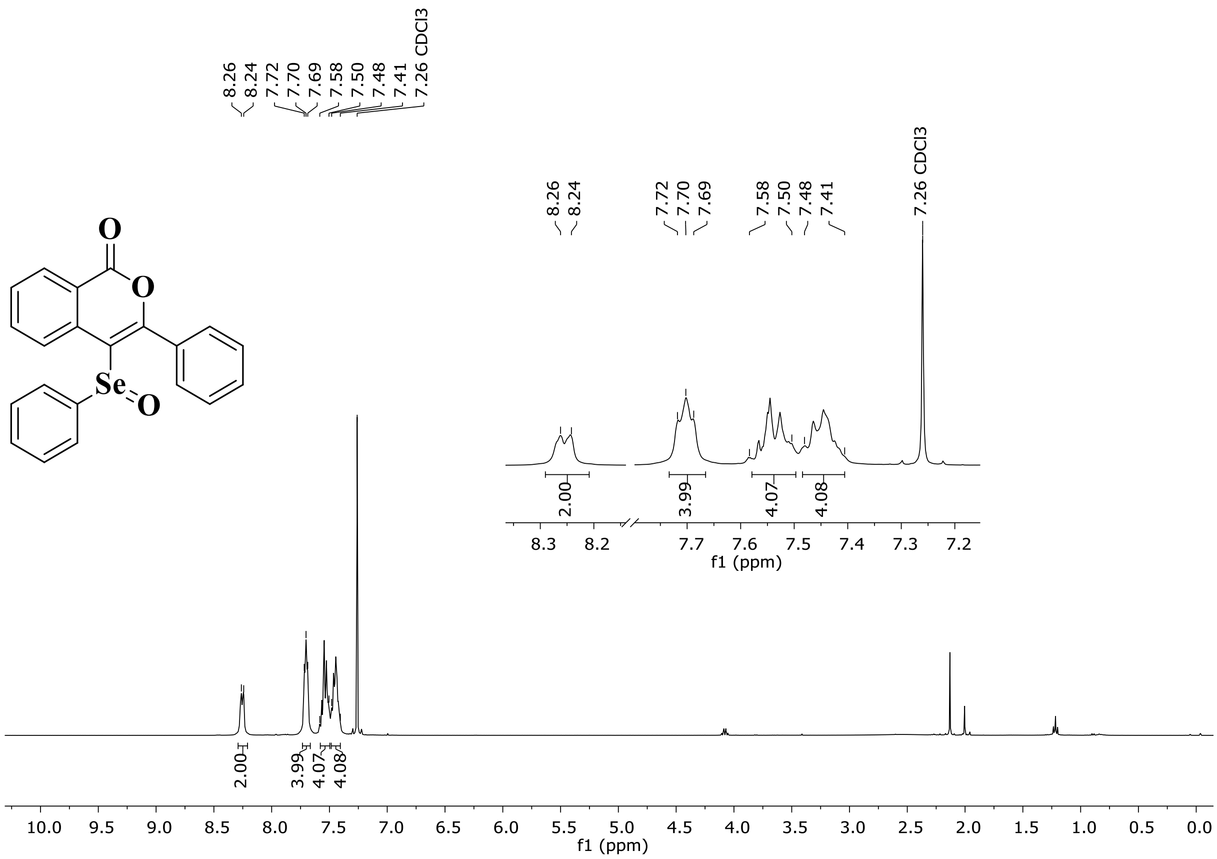

**Figure S81.** $^{1}H$ NMR spectrum (400 MHz) of compound **4a** obtained in $CDCl_3$.

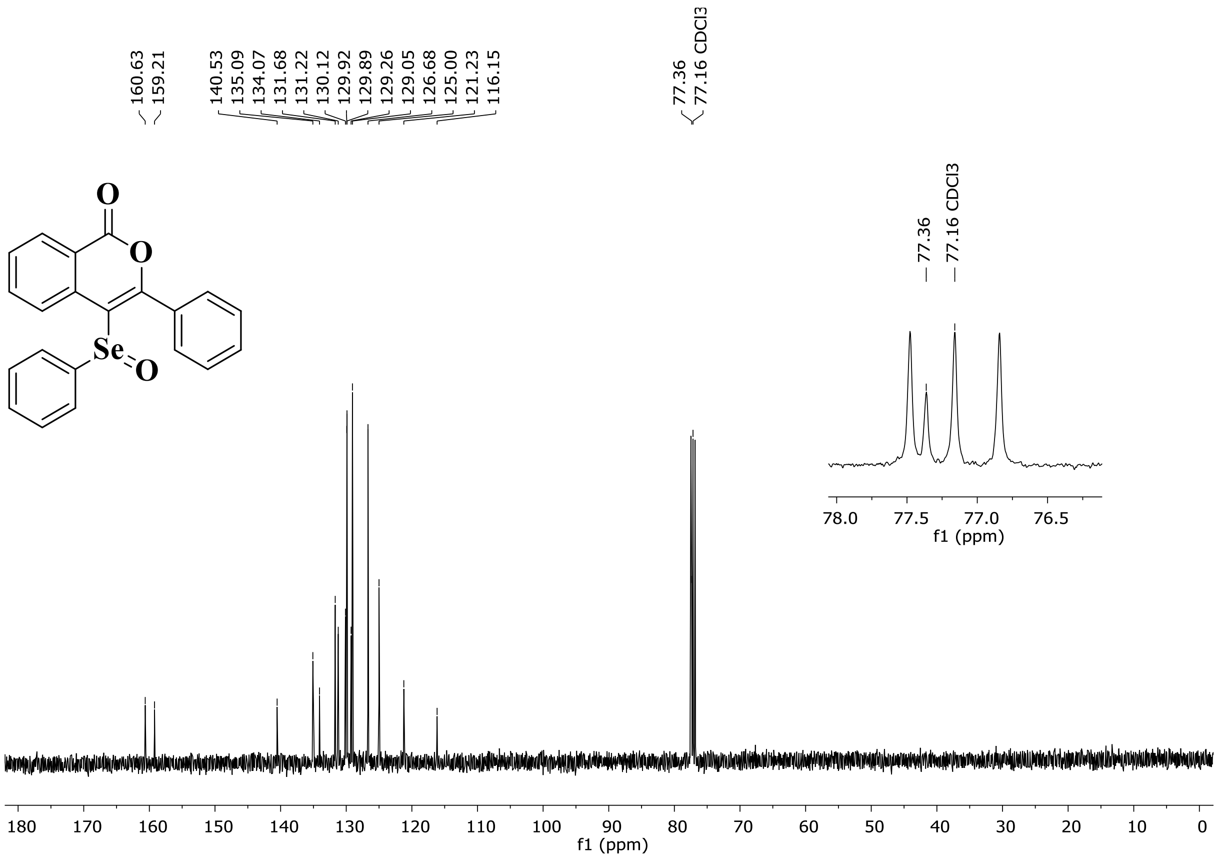

**Figure S82.** $^{13}C$ NMR spectrum (101 MHz) of compound **4a** obtained in $CDCl_3$.

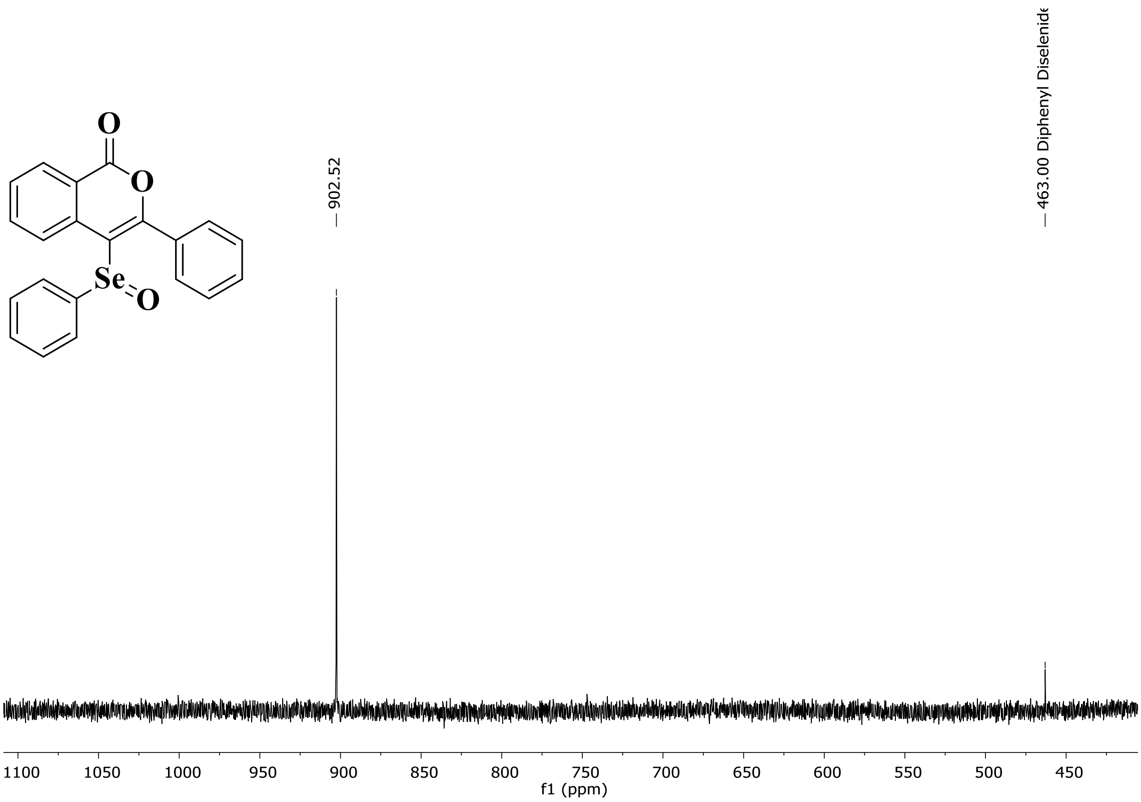


**Figure S83.** $^{77}$Se NMR spectrum (76 MHz) of compound **4a** obtained in $CDCl_3$.

## References

(1) Huang, Y.; Zhang, X.; Dong, X. Q.; Zhang, X. Iridium-Catalyzed Cycloisomerization of Alkynoic Acids: Synthesis of Unsaturated Lactones. *Adv. Synth. Catal.* **2020**, *362* (4), 782–788. https://doi.org/10.1002/adsc.201901322.

(2) Yao, T.; Larock, R. C. Synthesis of Isocoumarins and α-Pyrones via Electrophilic Cyclization. *J. Org. Chem.* **2003**, *68* (15), 5936–5942. https://doi.org/10.1021/jo034308v.

(3) Goulart, H. A.; Neto, J. S. S.; Barcellos, A. M.; Silva, K. B.; De Moraes, M. C.; Jacob, R. G.; Lenardão, E. J.; Barcellos, T.; Perin, G. Synthesis of 4-Selanyl- And 4-Tellanyl-1 H-Isochromen-1-Ones Promoted by Diorganyl Dichalcogenides and Oxone. *J. Org. Chem.* **2021**, *86* (20), 14016–14027. https://doi.org/10.1021/acs.joc.1c00271.

(4) Jin, G.-Q.; Gao, W.-X.; Zhou, Y.-B.; Liu, M.-C.; Wu, H.-Y. Synthesis of Selenated Isochromenones by AgNO 3 -Catalyzed Three-Component Reaction of Alkynylaryl Esters, Selenium Powder and ArB(OH) 2. *RSC Adv.* **2020**, *10* (51), 30439–30442. https://doi.org/10.1039/D0RA06016J.

(5) Lin, X.; Fang, Z.; Zeng, C.; Zhu, C.; Pang, X.; Liu, C.; He, W.; Duan, J.; Qin, N.; Guo, K. Continuous Electrochemical Synthesis of Iso-Coumarin Derivatives from o -(1-Alkynyl) Benzoates under Metal- and Oxidant-Free. *Chem. – A Eur. J.* **2020**, *26* (60), 13738–13742. https://doi.org/10.1002/chem.202001766.
